# High-throughput phenotype-to-genotype testing of meningococcal carriage and disease isolates detects genetic determinants of disease-relevant phenotypic traits

**DOI:** 10.1128/mbio.03059-24

**Published:** 2024-10-30

**Authors:** Robeena Farzand, Mercy W. Kimani, Evangelos Mourkas, Abdullahi Jama, Jack L. Clark, Megan De Ste Croix, William M. Monteith, Jay Lucidarme, Neil J. Oldfield, David P. J. Turner, Ray Borrow, Luisa Martinez-Pomares, Samuel K. Sheppard, Christopher D. Bayliss

**Affiliations:** 1 Department of Genetics and Genome Biology, University of Leicester, Leicester, United Kingdom; 2 Department of Biology, University of Oxford, Oxford, United Kingdom; 3 Zoonosis Science Center, Department of Medical Sciences, Uppsala University, Uppsala, Sweden; 4 The Milner Centre of Evolution, Department of Life Sciences, University of Bath, Bath, United Kingdom; 5 Meningococcal Reference Unit, UK Health Security Agency, Manchester, United Kingdom; 6 School of Life Sciences, University of Nottingham, Nottingham, United Kingdom; Fondazione Biotecnopolo di Siena, Siena, Italy

**Keywords:** GWAS, phase variation, *Neisseria meningitidis*, genotype-to-phenotype, pathogenesis

## Abstract

Genome-wide association studies (GWAS) with binary or single phenotype data have successfully identified disease-associated genotypes and determinants of antimicrobial resistance. We describe a novel phenotype-to-genotype approach for a major bacterial pathogen that involves simultaneously testing for associations among multiple disease-related phenotypes and linkages between phenotypic variation and genetic determinants. High-throughput assays quantified variation among 163 *Neisseria meningitidis* serogroup W ST-11 clonal complex isolates for 11 phenotypic traits. A comparison of carriage and two disease subgroups detected significant differences between groups for eight phenotypic traits. Candidate genotypic testing indicated that indels in *csw*, a capsular biosynthesis gene, were associated with reduced survival in antibody-depleted heat-inactivated serum. GWAS testing detected 341 significant genetic variants (3 single-nucleotide polymorphisms and 338 unitigs) across all traits except serum bactericidal antibody-depleted assays. Growth traits were associated with variants of capsular biosynthesis genes, carbonic anhydrase, and an iron-uptake system while adhesion-linked variation was in *pilC2*, *marR,* and *mutS*. Multiple phase variation states or combinatorial phasotypes were associated with significant differences in multiple phenotypes. Controlling for group effects through regression and recursive random forest approaches detected group-independent effects for *nalP* with biofilm formation and *fetA* with a growth trait. Through random forest testing, nine phenotypes were weakly predictive of MenW:cc11 sub-lineage, original or 2013, for disease isolates while three characteristics separated carriage and disease isolates with >80% accuracy. This study demonstrates the power of combining high-throughput phenotypic testing of pathogenically relevant isolate collections with genomics for identifying genetic determinants of specific disease-relevant phenotypes and the pathobiology of microbial pathogens.

**IMPORTANCE:**

Next-generation sequencing technologies have led to the creation of extensive microbial genome sequence databases for several bacterial pathogens. Mining of these databases is now imperative for unlocking the maximum benefits of these resources. We describe a high-throughput methodology for detecting associations between phenotypic variation in multiple disease-relevant traits and a range of genetic determinants for *Neisseria meningitidis*, a major causative agent of meningitis and septicemia. Phenotypic variation in 11 disease-related traits was determined for 163 isolates of the hypervirulent ST-11 lineage and linked to specific single-nucleotide polymorphisms, short sequence variants, and phase variation states. Application of machine learning algorithms to our data outputs identified combinatorial phenotypic traits and genetic variants predictive of a disease association. This approach overcomes the limitations of generic meta-data, such as disease versus carriage, and provides an avenue to explore the multi-faceted nature of bacterial disease, carriage, and transmissibility traits.

## INTRODUCTION

Low-cost whole-genome sequencing has revolutionized infectious disease epidemiology leading to major successes in the identification of vaccine antigens, antibiotic resistance genes, and determinants of disease (1). Mining this genomic data through genome-wide association studies (GWAS) is a powerful approach that has ascertained genetic determinants for phenotypes such as virulence, antibiotic resistance, food chain survival, and growth characteristics (2–5, 6). By combining GWAS and other genetic analyses with high-throughput phenotyping, we demonstrate the applicability of these methodologies to identifying genetic determinants of variation in multiple disease-associated phenotypes and assessing how genetic variation shapes behaviors of natural bacterial populations.

Multiple bacterial pathogens cause life-threatening invasive diseases by translocating from the upper respiratory tract into other sites. *Neisseria meningitidis* (the meningococcus) is a frequent colonizer of the nasopharynx with asymptomatic carriage rates of ~25% in 18- to 25-year-olds (7). Meningococci can translocate across epithelial and endothelial barriers into the bloodstream/cerebrospinal fluid where rapid replication leads to septicemia and/or meningitis (8). Invasive meningococcal disease (IMD) is most prevalent in infants after the waning of protective maternal antibodies, due to limited protective immunity, with a second peak in early adulthood associated with high carriage levels (9). Disease potential is linked to general ill-defined traits (e.g., systemic spread and transmissibility) and to experimentally defined traits including adhesion to host cells, resistance to serum bactericidal antibody activity, iron acquisition, and immune cell activation (8). Capsular group and genetically determined variability in these traits are thought to underpin differences between meningococcal lineages for causing IMD with several lineages, including the ST-11 complex (cc11), being classified as hypervirulent (8). From 2010, globally significant increases in IMD cases occurred due to the emergence and worldwide spread of a serogroup W (MenW) cc 11 lineage (10, 11). The emergence of this lineage was in part due to capsular switching from MenC cc11 strains (12). Within the UK, the “original UK strain” (original) emerged in 2009 and evolved into a novel “2013_strain” (2013) that subsequently became the dominant cause of UK MenW IMD cases (13, 14). The phenotypic traits underlying the spread and hyper-invasiveness of these MenW:cc11 sub-lineages are uncertain although this lineage is highly virulent in model systems (15). One suggestion, arising from analyses of epidemiological carriage data, is that the 2013_strain has evolved to higher transmissibility (16).

Routine whole-genome sequencing of meningococcal isolates from UK IMD cases and large-scale carriage studies have generated near-complete meningococcal genomes encompassing ~40,000 carriage and disease isolates that are publicly available through the PubMLST *Neisseria* database and Meningitis Research Foundation Meningococcus Genome Library (17). These resources provide exceptional opportunities for studying meningococcal epidemiology and genome dynamics. Limited coverage of repetitive DNA sequences has, however, hampered analyses of the simple sequence repeats (SSR) responsible for phase variation (PV) of ~60 meningococcal genes (18–20). Located within coding sequences or promoter regions, the hypermutability of these SSRs generates high-frequency and reversible changes in repeat number that produce translational or transcriptional ON-OFF switches in gene expression. Meningococcal phase-variable genes primarily encode single-copy outer membrane proteins (OMP), multi-copy OMP proteins, or enzymatic modifiers of major surface molecules (19, 20). PV of these genes impacts meningococcal carriage and disease attributes (21).

Meningococcal GWAS approaches have focused on lineage or disease-carriage comparisons. Utilizing a novel treeWAS approach with 129 MenC:cc11 isolates, Collins and Didelot detected associations between 12 genes and disease/carriage states (22). In a similar study of 261 MenC:cc11 isolates, Earle et al. detected a linkage of 7 SNPs and 465 kmers, harbored in 17 loci, with these states but also validated associations of *fba-fHbp* variation with IMD isolates and demonstrated that fHbp intergenic region (IGR) variation was a key virulence determinant (23). Finally, two recent studies detected a linkage of variation in the transferrin-binding protein with IMD (4, 24). One limitation of these studies is the absence of phenotypic data or more detailed meta-data. Effects of genetic variation on phenotypes of circulating meningococcal populations have had to be extrapolated from experiments with single isolates and are at risk from epistatic effects of multiple-interacting genetic variants. To overcome these limitations, we have combined high-throughput phenotypic testing with GWAS, machine learning, and focused genetic analyses of PV genes to improve the identification of genetic determinants of disease-associated behaviors in this major bacterial pathogen.

## RESULTS

### Comparison of allelic variation and PV states of meningococcal isolates selected for phenotype-to-genotype testing

**Fig 1:**
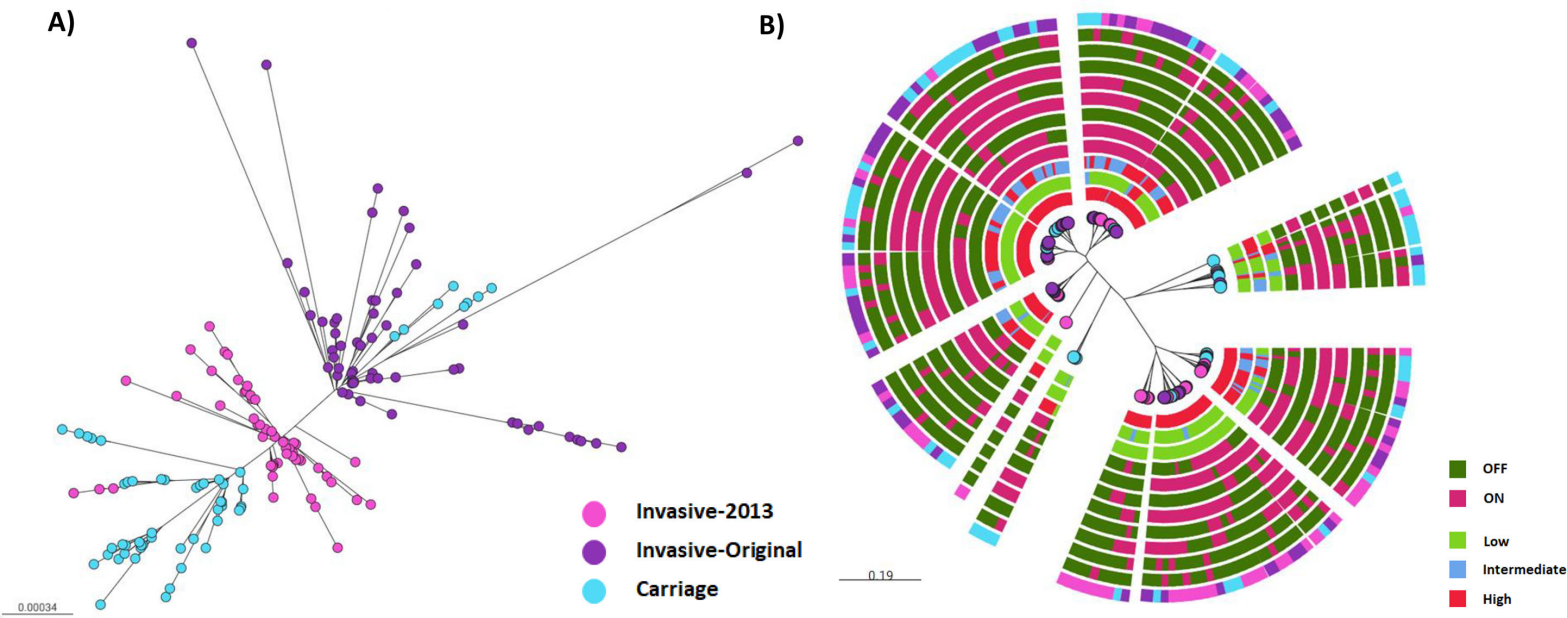
Phylogeny of MenW:cc11 UK carriage and disease isolates utilized in phenotype-to-genotype analysis. This figure shows the phylogenetic relationships of the isolates as determined from whole-genome sequence data in panel A and from the expression states of 11 phase-variable genes in panel B. The genome sequence data and PV states were obtained either from previous publications or generated during this study (see Materials and Methods). Panel (A) Phylogenetic tree of the 163 MenW:cc11 isolates utilized in the current study (purple dots, invasive, original isolates; pink dots, invasive, 2013 isolates; light blue dots, carriage isolates). Note that the carriage isolates encompass both lineages. Panel (B) A phylogenetic tree for the 163 MenW:cc11 isolates derived utilizing the combinatorial PV states for nine phase-variable outer membrane proteins and two DNA methyltransferases (PV states of each gene are shown for each cluster—dark green, OFF state; magenta, ON state; red, high expression; blue, intermediate expression; green, low expression) in the order from the second outer ring inwards of *modB*, *modA*, *pilC2*, *pilC1*, *hmbR*, *mspA*, *nalP*, *hpuA*, *porA*, *nadA*, and *fetA*. The outermost ring corresponds to the tips of the phylogenetic tree and represents the group that each isolate belongs to (color coding for the dots in the phylogenetic tree is the same as for panel A).

To explore genetic determinants of disease-associated attributes by GWAS and PV analyses, we selected hypervirulent MenW:cc11 meningococcal isolates as high levels of genetic relatedness limit confounding effects in GWAS assays. Our contemporaneous set of 163 isolates was split into three groups—two phylogenetically divergent invasive groups and a carriage group that was phylogenetically mixed but where all isolates were recovered from identical host sites (Fig. 1A). The invasive groups, original (*n* = 57) and 2013 (*n* = 52), represent an increase in disease due to either altered invasiveness or transmissibility (see Introduction). These isolates were obtained from the blood or cerebrospinal fluid (CSF) of patients (data not shown). The carriage isolates (*n* = 54) were obtained from nasopharyngeal swabs of Nottingham University studies and include both original and 2013 isolates (Fig. 1A). Thus, three potential endpoints were obtainable with these groups: ( i) genomic determinants of phenotypic variation; (ii) phenotypic and genomic determinants of disease evolution; and (iii) phenotypic and genomic determinants of disease-to-carriage transitions. These isolates exhibited diversity in the core genomes (Fig. 1) and were also genetically divergent from previously studied MenC:cc11 isolates (Fig. S1). To determine whether PV expression states were evolving independently of the core genomes, a phylogenetic tree was derived from the expression states of 11 PV genes (Data File S1). This analysis identified isolated clusters with distinctive PV expression patterns (Fig. 1B). Congruence testing detected divergence between PV and genome-based clusters (data not shown). This divergence indicated that PV expression states and genomic lineage were not linked and could be used for independent evaluation of associations between allelic variants or PV states and phenotypic variation.

### High-throughput assays of phenotypic variation in a large meningococcal isolate set

Experimental assays were selected to test for variability in phenotypic traits associated with meningococcal carriage and disease in humans. A key step was the preparation of replicate inoculum populations of multiple isolates in microtiter plates and prepared from one culture grown to the mid-exponential phase (Fig. S2) (25). This format ensured both high throughput and reproducibility as assays were initiated from similar “starter” populations. Assays encompassed growth in both brain heart infusion (BHI) and Roswell Park Memorial Institute (RPMI) media, representing high- and low-nutrient conditions, biofilm formation, release of lactate dehydrogenase (LDH) activity, adhesion to A549 epithelial cells, and a serum bactericidal antibody-depleted (SBAD) assay. Lactate has multiple influences on meningococcal virulence while autolysis contributes to extracellular DNA release and biofilm formation (26, 27). Thus, the LDH assay provided a high throughput combined measure of LDH levels and autolysis. The SBAD assay is a modified serum bactericidal antibody assay with reduced potential for confounding effects from differing levels of bactericidal antibody-binding antigens on bacterial surfaces. Reduced recovery of viable bacteria in antibody-depleted heat-inactivated (AD-HI) controls relative to the inoculum led to a separate assessment of an AD-HI serum sensitivity phenotype. The final data set consisted of 11 phenotypic trait outputs for all 163 MenW cc11 isolates (Data File S2).

### Significant differences in multiple phenotypes between disease and carriage isolates

**Fig 2:**
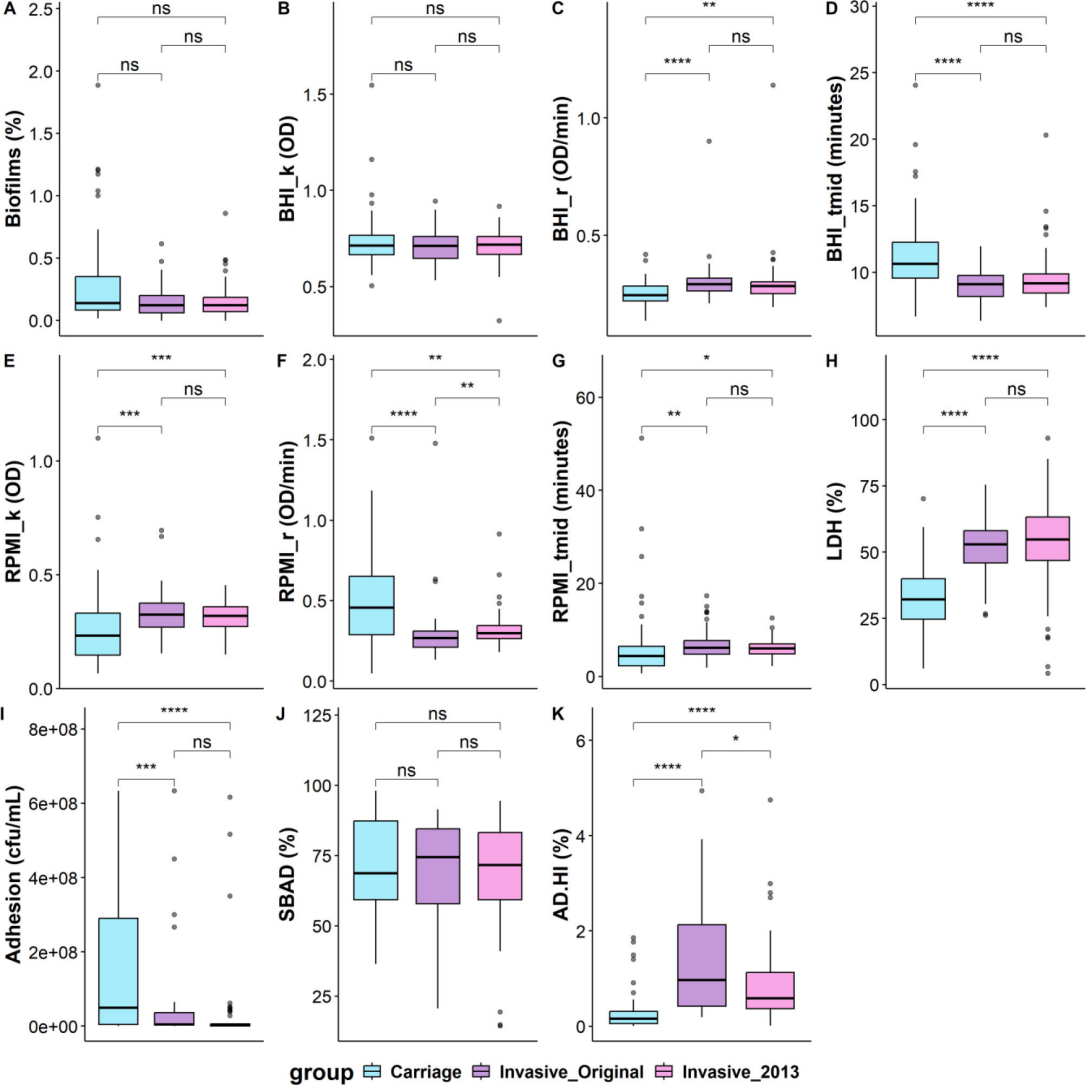
Association of source or phylogenetic group with phenotypic variation of MenW:cc11 UK disease and carriage isolates. Data were obtained for 11 phenotypic traits from six assays for 163 isolates of MenW:cc11 lineage. These data were tested for significant differences arising from source (carriage versus invasive isolates) and invasive sub-lineage (original versus 2013 isolates). The compositions of these three groups were as follows: 57 invasive original, 52 invasive 2013, and 54 carriage isolates. Phenotypic assays: (A) Biofilm formation (% of crystal violet staining relative to isolate B141); (B–G) growth assays (B–D, BHI; E–G, RPMI; k, OD for maximum growth plateau; r, replication rate in OD/min; T-mid, minutes to mid-log phase); (H) released LDH activity (% of total LDH activity); (I) adhesion to A549 cells (cfu/mL); (J) SBAD resistance (% of CFU counts following incubation in antibody-depleted serum versus heat-inactivated antibody-depleted serum); (K) AD-HI serum sensitivity (% of CFU counts following incubation in heat-inactivated antibody-depleted serum versus inoculum). Panels (A–K) Phenotypic variation between isolate groups for 11 phenotypic traits. Bar, median; box, interquartile range; line, minimum and maximum values; dots, outliers. Statistical significance was determined using a Wilcoxon Rank-Sum test and the Kruskal-Wallis test for comparing two states or multiple states, respectively. *P < 0.05; **P < 0.01; ***P < 0.001. Significant differences were detected for BHI_r, BHI_tmid, RPMI_k, RPMI_r, RPMI_tmid, LDH, adhesion, and AD-HI serum sensitivity.

Most of the phenotypic traits were shown to vary independently of each other through covariance testing (Fig. S3). Weak positive correlations between carrying capacity and lag phase for RPMI and weak negative correlations between replication rate and lag phase for both media were indicative of an interplay between growth parameters. Having established the extent of phenotypic correlations, we examined the three isolate groups for divergence in phenotypic traits. Significant differences were found between both invasive groups and the carriage group for eight traits and between the two invasive groups for two traits (Fig. 2). Carriage isolates exhibited lower replication rates and longer lag phases in BHI media, contrasting increases and decreases in all RPMI traits, lower levels of released LDH activity, and higher adherence to A549 cells. In two of the assays, AD-HI serum sensitivity and RPMI replication rate, there were significant differences between all three groups with AD-HI serum sensitivity being highest in the carriage isolates and lowest in the original invasive isolates. The distribution of phenotypic variation across the lineages was examined by mapping scores for individual phenotypes onto the phylogenetic tree (Fig. S4). No obvious interactions between phenotypes were observed apart from high adhesion and low AD-HI serum sensitivity co-locating for some carriage isolates.

### Insertions or deletions in a MenW capsule biosynthetic gene as determinants of extreme phenotypic variation

**Fig 3:**
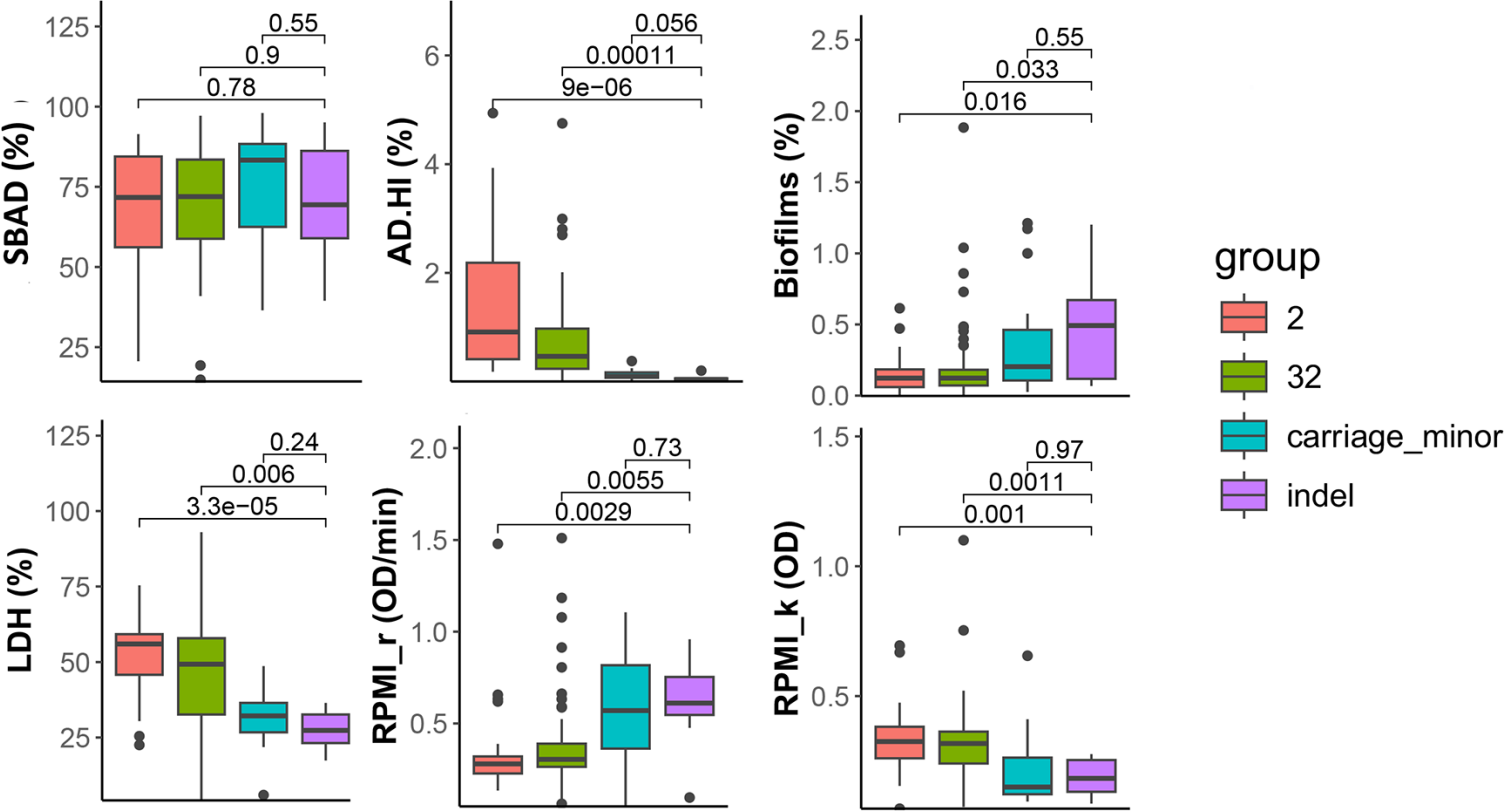
Association of csw deletions and minor allele variation with sensitivity to AD-HI serum sensitivity. The capsule is a major meningococcal disease determinant. This figure shows associations between variation in six phenotypic traits and allelic variants of csw, a gene critical for biosynthesis of the MenW capsule (the data for the other five phenotypic traits are non-significant, see Fig. S5). Variation includes indels that result in translational inactivation of this gene. Allele types for the csw gene were obtained for all isolates from the Neisseria PubMLST database (Fig. S6). The majority of invasive isolates and carriage isolates contained either allele 2 (*n* = 48 disease; three carriage) or allele 32 (*n* = 49 disease; 28 carriage). A sub-set of carriage isolates contained novel minor alleles with either single nucleotide differences (*n* = 11) or indel mutations (*n* = 8) referred to as the carriage and indel group, respectively. A one-way ANOVA was applied across each of these four groupings for each of the phenotypic traits; *P* values for comparison of the indel group to all other groups are provided. Significant differences were observed between the indel group and both major capsular allele groups at a range of levels: *P* < 0.001, AD-HI serum sensitivity; *P* < 0.01, LDH, RPMI_k, RPMI_r; *P* < 0.05, biofilms.

The sensitivity of some isolates in the AD-HI assay was suggestive of inactivation of capsular biosynthesis, a major determinant of meningococcal serum resistance (28). Comparisons of capsular locus gene sequences detected very limited allelic variation in all these genes except *csw*, which is responsible for the final MenW capsule assembly step [(29); Data File S3]. Two major allelic variants of *csw* were found [these alleles co-segregate with the original and 2013 invasive groups (13)] plus several minor alleles including indel mutations. Comparative analyses of phenotypic variation for the major and minor *csw* alleles detected a highly significant association of indel mutations with AD-HI serum sensitivity but not SBAD sensitivity (Fig. 3). The indel mutants were also associated with higher biofilm formation and replication rates in RPMI media (RPMI_r) and lower LDH release and RPMI carrying capacity (RPMI_k) but at a lower level of significance (Fig. 3). In carriage isolate comparisons, only AD-HI serum sensitivity was significantly different between the indel mutations and major alleles (Fig. S5). Phylogenetic analyses detected a scattered distribution of the indel mutations indicating that this trait was linked to *csw* inactivation, not a clone-specific effect (Fig. S6).

### A genome-wide association test for allelic gene variation as a determinant of phenotypic variation

**Fig 4:**
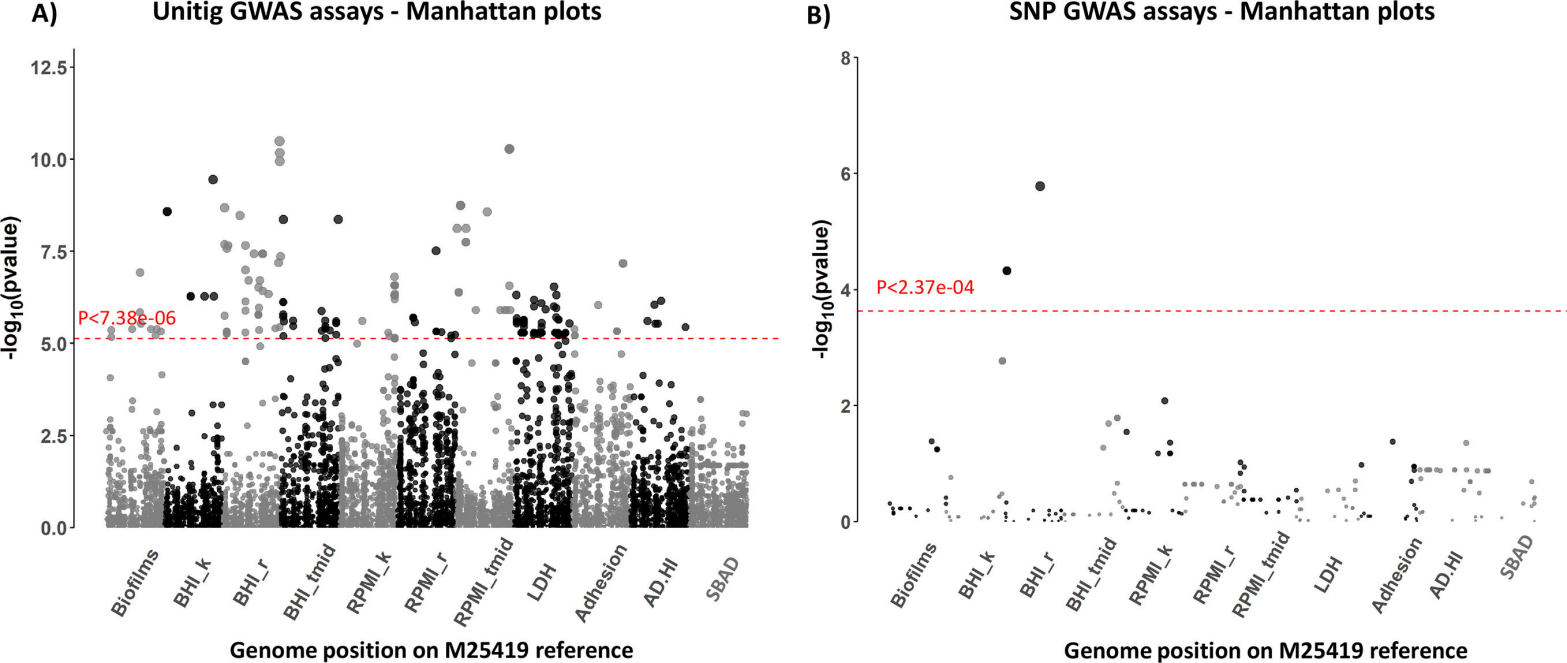
Manhattan plot displaying significant GWAS hits for each phenotype. GWAS assays were performed with unitigs (**A**) or SNPs (**B**) obtained from pyseer or snippy, respectively, by mapping assembled sequences for the 163 MenW:cc11 isolates to the M25419 genome sequence. Genetic variants were filtered for a minor allele frequency of >5% and adjusted for population structure relative to a recombination-corrected phylogenetic tree prior to association testing with FaST-LMM. Panel (A) Unitig-based GWAS; each circle represents a hit with a specific nucleotide (i.e., a unitig) whose lengths range from 10 to 1,000 base pairs. Panel (B) SNP-based GWAS; each hit represents a single nucleotide variant. Unitigs and SNPs for each assay are plotted relative to the position in the M2549 genome sequence. Assays are as indicated in Fig. 2. The red dotted line shows the Bonferroni-corrected significance level.

Previous meningococcal GWAS studies have utilized binary characteristics for detecting associations. To detect genomic determinants of variation in the 11 phenotypic traits, we utilized continuous phenotypic data combined with meningococcal genetic variation in single nucleotides or sequences of varying length (unitigs). Genetic data, comprising 1,810 genes conserved across all isolates, were obtained by mapping assembled contigs to the closed genome sequence of M25419 (ID:47007; CP016678.1), a MenW cc11 isolate of the original sub-lineage (30). Utilizing linear mixed-effect models with SNPs or unitigs, we found that genetic variation explained differing proportions of the phenotype variance for each trait (Table 1; highest, BHI_r, h^2^ = 98%; lowest, RPMI_tmid, h^2^ = 0%). GWAS testing was performed with 14,800 unitigs and 211 SNPs (Fig. 4). Following Bonferroni correction, we observed associations for 338 unitigs (*P* < 7.38E-06) and three SNPs (*P* < 2.37E-04) (Data File S4). All significant SNPs were associated with lower BHI_r values. Two produced non-synonymous polymorphisms in *cssA* (*NEIS0054*), a capsule biosynthesis gene, while the other was a synonymous polymorphism within the excinuclease ABC subunit C (*uvrC*, *NEIS1263*) gene.

**TABLE 1:** Significant unitigs associated with divergent MenW phenotypic traits^*f*^

^*a*^Phenotype	% ^*b*^Heritability (h²)	Total gene hits	Total unitig hits	Number, location and type of unitig hits					
				^*c*^IGR	Single copy genes			Multicopy genes	^*e*^PV
					^*d*^ORF synonymous variation	^*d*^ORF Non-synonymous variation	^*d*^ORF non- specific		
Adhesion	39	5	10	2	-	-	3	5	-
Biofilm	27	4	10	6	-	1	1	1	1
BHI_k	2	1	7	5	-	2	-	-	-
BHI_r	98	19	53	9	15	13	12	2	1
BHI_tmid	27	0	22	22	-	-	-	-	-
RPMI_k	10	9	75	9	43	16	7	-	-
RPMI_r	35	9	64	6	37	14	4	2	-
RPMI_tmid	0	2	21	19	-	-	-	2	-
LDH	58	5	70	59	-	-	-	11	-
SBAD	14	-	-	-	-	-	-	-	-
AD-HI	52	5	6	1	1	1	2	1	-

^*a*^ Pheno, phenotype (note the SBAD assay had no significant unitig hits).

^*b*^ Heritability (h^2^), this is the proportion of variance in phenotype explained by the genetic variation when maximizing the log-likelihood.

^*c*^ IGR, these are unitig hits within intergenic regions.

^*d*^ ORF, these are unitig hits within the open reading frame.

^*e*^ PV, indicates a unitig containing a phase-variable repeat tract; non-specific, unitigs where variation differed among the isolates lacking the reference unitig sequence.

^*f*^ Significance values, derived using a linear mixed-effect model, for all SNPs and unitigs can be found in Data File S4.

For the unitigs, significant associations were detected for every phenotype except the SBAD assay and encompassed hits in coding regions or intergenic regions (IGR) of 59 genes (Table 1). Unitigs were distributed among single-copy genes (51%; 174/338), multicopy genes (7%; 24/338), phase-variable repeat tracts (1%; 2/338), and IGR (41%; 138/338). In all, 40 single-copy gene hits mapped to regions of high variability (termed non-specific). Six unitigs mapped to transposases while the BHI_tmid, RPMI_tmid, and LDH phenotypes were only associated with unitigs in IGR or multi-copy genes. These non-specific, multi-copy, IGR-biased, and transposase unitigs may have arisen due to mapping to genomic regions that are difficult to assemble from Illumina sequence data.

Four phenotypes exhibited hits in single-copy and PV genes. Adhesion was associated with a non-synonymous substitution in the *pilC2* gene (*NEIS0033*) and variation in *marR* and *mutS*. One biofilm unitig was in a hypothetical protein (cds-ANX87421.1) while the other contained the polyC repeat tract of *nalP* (*NEIS1943*), a phase-variable autotransporter. Two nonsynonymous changes in a putative membrane protein (*NEIS0005*) and variations in four IGRs were associated with carrying capacity in BHI media. The AD-HI serum sensitivity phenotype had hits with a non-synonymous substitution in *sucA* (*NEIS0931*, 2-oxoglutarate dehydrogenase E1 component) and other types of variation in *NEIS0837* (hypothetical protein), *aldA* (*NEIS1942*), *NEIS0516* and *NEIS3042*.

Three of the growth phenotypes exhibited high numbers of unitigs. Significant unitigs for growth rate in BHI media (BHI_r) contained non-synonymous substitutions in genes involved in capsule assembly (*NEIS0055; ctrA*), transcription (*NEIS0059*), DNA mismatch repair (*NEIS2138; mutS*), LOS biosynthesis (phase-variable gene *lgtG*, *NEIS2011*), and a hypothetical protein (*NEIS0105*). In addition, high levels of genetic variants were observed in carbonic anhydrase (*NEIS2004*) and subunits B (*NEIS1269*) and C (*NEIS1263*) of the ABC excinuclease (UvrABC). For the other two growth phenotypes, the significant unitig hits spanned multiple genes across large genome fragments. Carrying capacity in RPMI media (RPMI_k) had multiple hits, both synonymous and non-synonymous, within a region encoding the enterobactin receptor (*NEIS1963; fetA*), an ABC transporter system (*NEIS1964* to *NEIS1968*), and *nadA* (*NEIS1969*). The replication rate in this media (RPMI_r) was associated with a similar mix of substitutions in a region encoding a chromosome segregation protein (SMC; NEIS0484), alcohol dehydrogenase (NEIS0486; AdhA), type IV pilin protein (NEIS0487; PilV), and a macrolide ABC transporter (NEIS0488/0489; MacAB).

A generic observation was that much of the significant non-synonymous genic variation was driven by small numbers of isolates (2–14) and that significant unitigs were present or absent in the same isolates for some phenotypes with similar summary statistics being indicative of linkage disequilibrium (Fig. S7 and S8). Overall, the total proportion of variance in phenotype explained by unitig genetic variation was considerable, whereas individual unitigs tended to confer low levels of heritability suggestive of the presence of other predictors of phenotypic variation.

### Associations of phenotypic variation with PV expression states and phasotypes

**Fig 5:**
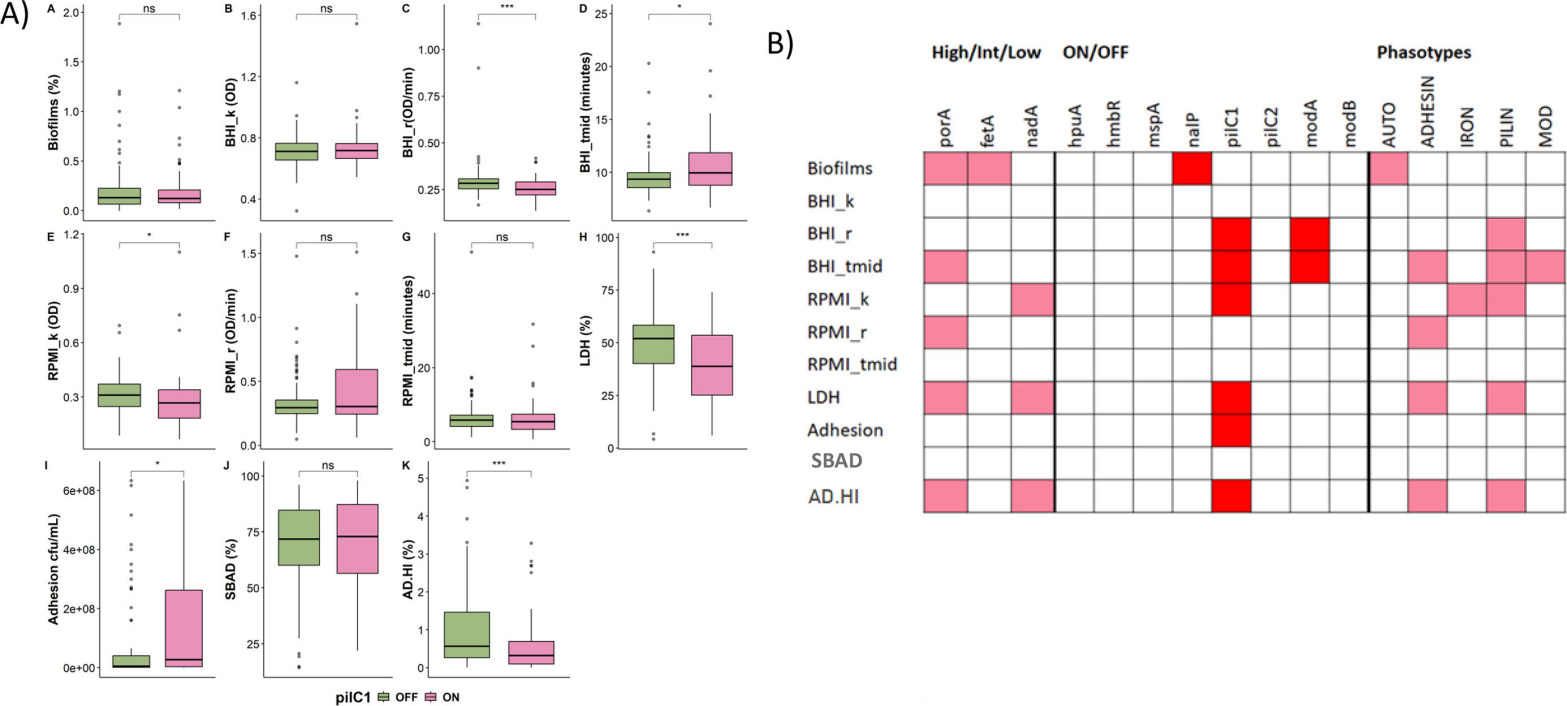
Association of pilC1 and other phase-variable OMP expression states with phenotypic variation. To assess whether PV is a determinant of phenotypic variation, the expression states of individual PV genes and combinations of PV genes (termed phasotypes) were tested for significant differences for the 11 traits. The *pilC1* data are shown as an example in panel (A) and a summary of all significant data in panel (B). The expression states of each PV gene were determined for the 163 isolates by a combination of GeneScan and genomic analyses of the repeat tracts. The repeat tracts are located in either the promoter (*n* = 3) or reading frames (*n* = 8) of this gene resulting in three (high, intermediate, low) or two (ON, OFF) expression states, respectively. Phasotypes were generated from the expression states of five combinations of phase-variable genes: IRON, *fetA*, *hpuA*, and *hmbR*; ADHESIN, *nadA* and *porA*; AUTO, *nalP* and *mspA*; PILIN, *pilC1* and *pilC2*; and MOD, *modA* and *modB*. Phenotypic values were compared for isolates grouped by expression state of individual genes and phasotypes using a Wilcoxon Rank-Sum test and the Kruskal-Wallis test for comparing two states or multiple states, respectively. Panel (A) Plots of the 11 sets of phenotypic data for the two expression states of *pilC1*. Plots: Bar, median; box, interquartile range; line, minimum and maximum; dots, outliers. *P* values: *P < 0.05; **P < 0.01; ***P < 0.001; ns, not significant. Panel (B) Tabular display of significant differences (*P* < 0.05 or lower) in phenotypic data between expression states of individual genes and phasotypes. Red filled box, ON/OFF, all three promoter-located expression states or a phasotype. Pink-filled box, one or two promoter-located expression states. Unfilled boxes, no significant differences.

**Fig 6:**
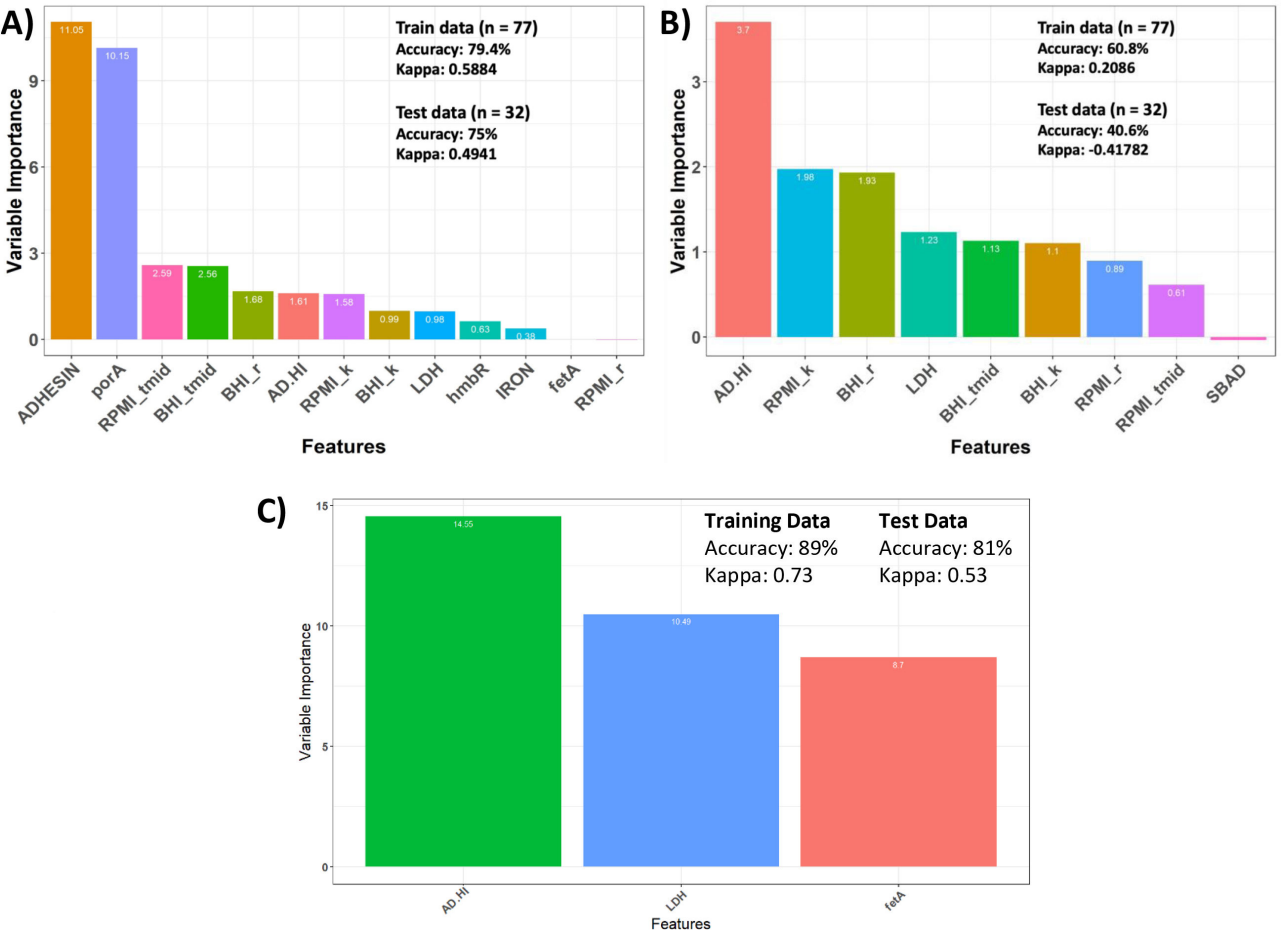
Using phenotypic variation, PV states, and phenotypes to predict disease evolution and potential. The phenotypic traits and PV expression states were tested for utility in predicting whether isolates were from a specific invasive group (**A and B**) or from a specific source (**C**) with the former predicting disease evolution and the latter disease potential. Recursive Feature Elimination with random forest analysis was utilized to predict invasive isolate group (i.e., original or 2013) based on phenotypic variation and/or PV states. The PV states were inputted as both the expression states of individual genes or as phasotypes—combinatorial expression states. Panel (A) important phenotypes and PV states in predicting invasive isolate group. Panel (B) important phenotypes in predicting invasive isolate group. Panel (C) important phenotypes and phasotypes/PV genes in predicting disease/carriage phenotype. The y-axis indicates the relative contribution of each determinant to the separation of isolates into these groups. Classification accuracy is the proportion of the outcomes that were correctly predicted. The Kappa values range from 1, complete concordance, to −1, complete discordance, while values near 0 indicate no relationship between model predictions and true results.

Meningococci have multiple phase-variable genes, we therefore investigated whether PV states were associated with variance in the 11 phenotypic traits. PV states of nine outer membrane proteins (*porA*, *fetA*, *nadA*, *hpuA*, *hmbR*, *mspA*, *nalP*, *pilC1,* and *pilC2*) and two DNA methyltransferases (*modA* and *modB*) were determined for the 163 MenW:cc11 isolates (Fig. S9). Expression states of each gene were individually analyzed for differences in distribution across groups and for all phenotypic traits (Fig. 5; Fig. S10 to S19). No statistically significant differences were detected for five of the PV genes (*hmbR, hpuA, modB, mspA,* and *pilC2*). Other genes were associated with significant differences in 1–8 phenotypes with *porA*, *nadA,* and *pilC1* exhibiting three or more significant effects (Fig. 5B). An important observation was the association of *pilC1* ON expression state with higher levels of adhesion but lower survival in AD-HI serum (Fig. 5A) and a moderate correlation with group (Fig. S20; 62% *pilC1* ON states were in carriage isolates). Intriguingly, the major phase-variable meningococcal OMP, *porA*, exhibited an association of high expression with lower production of LDH, lower survival in AD-HI serum, and contrasting effects on growth rate (higher in BHI and lower in RPMI). To test for combinatorial PV effects, we combined genes of related functions into five phasotypes (i.e., AUTO, ADHESION, IRON, PILIN, and MOD; Fig. 6) where a phasotype of, for example, two genes with ON/OFF states has four combinatorial expression states [ON-ON, ON-OFF, OFF-ON, OFF-OFF; see also (31)]. Each phasotype was associated with significant differences in at least one phenotypic trait while the PILIN and ADHESIN phasotypes affected five and four phenotypes, respectively (Fig. 6B; Fig. S21 to S25). No significant correlations were detected between phasotypes and group or single PV genes (Fig. S26 and S27).

### Confounding effects of strain source on associations between PV expression states and phasotypes with phenotypic variation

Although reduced by high switching rates and selection, confounding effects on phenotypic associations may arise from a linkage of individual PV gene states with variation in another PV or non-PV gene. To control for these factors, we performed ANOVA and linear regression models using isolate group (i.e., carriage, invasive_original and invasive_2013) as a proxy for allelic variation in other genes based on the observed phylogenetic divergence of these groups as seen in Fig. 1A. After Bonferroni correction (*P* < 0.0038), isolate group was the only determinant of phenotypic variation for five phenotypes (Table 2; Data_File_A5). However, we also detected two more complex effects; an association of a group:*nalP* interaction with biofilm formation; and independent associations of group, *fetA* and the IRON phasotype with carrying capacity in RPMI media. To further test for interactions, we implemented random forest models with Recursive Feature Elimination (RFE) to predict variables by regression for phenotype (Table 2). RFE identified multiple features as having relevance for phenotypes and confirmed the importance of group for six of the phenotypes, *nalP* for biofilm formation, and *fetA* for RPMI_k (Table 2; Fig. S28 and S29).

**TABLE 2:** Identification of phase-variable and non-phase-variable determinants of phenotypic states from statistical analyses and random forest models^*a*^

Phenotype	Type II/III ANOVA^*b*^ (*P* < 0.0038)	Linear regression (*P* < 0.05)	Random forest with recursive feature elimination (RFE)^*c*^	Concordant model features^*d*^
Biofilms	*nalP*:group, group	*porA, fetA, nalP,* AUTO	group, AUTO, *nalP*, *porA*	*nalP*
BHI_k	none	none	*pilC1*	none
BHI_r	none	none	group	none
BHI_tmid	group	*fetA, modA*, group	group, ADHESIN, MOD, *porA*	group
RPMI_k	group, *fetA*, IRON	group, *fetA,* IRON	group, *nadA, pilC1, modA, fetA*	group*, fetA*
RPMI_r	group	*fetA*, group	group, *fetA*, *pilC2*	group
RPMI_tmid	none	none	group, *hpuA*, *mspA*, *modA*,	none
LDH	group	group	group	group
Adhesion	group	group	group	group
SBAD	none	*hpuA*	*fetA*	none
AD-HI	group	*hmbR*, group	group	group

^*a*^ Determinants: group, invasive_original, invasive_2013 or carriage isolates; genes, ON/OFF or high/intermediate/low expression states; phasotypes, combined ON/OFF and/or high/intermediate/low expression states of 2 or more genes.

^*b*^ Type II/III ANOVA with Bonferroni correction.

^*c*^ Variables that RFE recommends for the best model with lowest root mean squared error.

^*d*^ Concordant model features are those detected as significant in the ANOVA, linear regression and RFE models.

### Specific phenotypes and PV states as determinants of invasive group and disease potential

To detect predictors of meningococcal disease evolution and potential, comparisons were performed between the two invasive groups and between all invasive isolates and carriage isolates, respectively. Implementation of RFE models identified 13 variables as predictive of the invasive isolate group with an accuracy of 75% and Kappa of 0.49 for the test data (Fig. 6A). As the ADHESIN phasotypes and *porA* PV states have the highest predictive values and *porA* is part of this phasotype, this model suggests that *porA* PV states are major drivers of differentiation of these groups. This could be due to many of the original isolates having a short 7C repeat tract in the *porA* promoter that locks these isolates into a high expression state (Data_File_A1). Simplification of this model to only include phenotypes indicated that these groups could be differentiated by AD-HI and multiple growth traits but with a low level of accuracy (Fig. 6B). A further iteration of this model identified three characteristics (two phenotypes and *fetA* PV state) as strong predictors of the disease and carriage states (Fig. 6C). No significant differences were detected between carriage isolates of the original and 2013 lineages for the LDH and AD-HI traits (data not shown), indicating that the high preponderance of 2013 carriage isolates in our collection is not the major driver of the disease potential model.

## DISCUSSION

Exploitation of extensive microbial genome databases and high-throughput sequencing technologies for real-time predictions of microbial disease potential requires extensive post-genomic phenotyping of representative isolate collections. By combining high-throughput phenotypic testing with multifaceted genetic analyses, we demonstrate that generalized phenotype-to-genotype testing is achievable and can predict genetic determinants of disease-relevant phenotypes and the relative contributions of phenotypes/genotypes to disease evolution and potential for pathogenic bacterial lineages. We discuss our findings in terms of predicting determinants of specific disease-associated phenotypes from genomic data and understanding the transmission and disease-causing abilities of a major pathogenic meningococcal lineage (16, 32).

Altered metabolic genes have been correlated with the epidemic spread of meningococci and IMD in the elderly (4, 33). In line with these observations, we found that MenW:cc11 isolates exhibit differential growth characteristics with carriage isolates having higher and lower average growth rates in RPMI and BHI, respectively, than invasive isolates. These differences may be indicative of invasive isolates having adapted to growth in the bloodstream that has a higher and more diverse nutrient profile than mucosal surfaces from where carriage isolates are obtained. Furthermore, we observed that 2013 invasive isolates had significantly higher replication rates in minimal media than original disease isolates, a phenotype that may facilitate transmission by rapidly increasing bacterial load on mucosal surfaces and a partial explanation for this sub-clone becoming a major agent of IMD (14). However, the differentiation of the two invasive isolate groups in the random forest was mainly driven by differences in the *porA* expression states with the phenotypes being only weak predictors of the evolution of the 2013 sub-group (Fig. 6).

Our GWAS analysis detected both SNPs and unitigs associated with growth differences that explain some of the phenotypic divergences between carriage and disease isolates. Reduced growth rate in BHI media was associated with nonsynonymous alterations in genes involved in capsule biosynthesis, DNA repair, and metabolism. The capsular genes included *cssA*, a UDP-N-acetylglucosamine 2-epimerase required for sialic acid biosynthesis and capsule production (34), and *ctrA*, a transporter involved in capsule extrusion (35). Alterations in these genes may modify capsular polysaccharide production with consequent effects on growth rate. Similarly, allelic variation in *mutS* may destabilize PV genes and alter growth characteristics as this gene encodes a major DNA mismatch repair protein whose modification is associated with heightened mutation rates, elevated PV rates, and evasion of host immune responses (36). Finally, growth may be affected by metabolic differences arising from variations in carbonic anhydrase (*NEIS2004),* a known regulator of meningococcal responses to carbon dioxide levels and maintenance of pH homeostasis (37). These latter variations are of particular interest as carbonic anhydrase inhibitors are being tested as novel therapeutics for gonococcal diseases (38). Non-over-lapping with BHI determinants, a lower growth rate in RPMI was associated with genetic variation across a locus encoding genes involved in resource utilization and metabolism. This locus contained nonsynonymous changes in *pilV*, a minor Type IV pilus subunit (39), but the phenotypic variation is probably explained by nonsynonymous changes in SMC, a chromosome segregation protein, alcohol dehydrogenase (*adhA*), and/or the *macA-macB* efflux system. Decreases in RPMI carrying capacity were associated with nonsynonymous variation in *fetA* and a genetically linked ABC transporter locus. These results are consistent with associations between the IRON phasotype and *fetA* PV state with differential RPMI_k values (Table 2; Fig. S12 and S24). The mechanism is uncertain as the known siderophore-ligands for FetA (40) are not present in our assays but suggest that iron metabolism impacts growth across this lineage. Associations between genetic variation in hypervirulent MenW:cc11 isolates and alterations in metabolic capabilities suggest that metabolic flexibility contributed to the rapid spread of this lineage within the UK population.

Another important output of our phenotype-to-genotype analyses was for potential determinants of serum resistance which is a major factor in meningococcal disease. A previous GWAS analysis of MenC:cc11 isolates associated allelic variation in the factor H binding protein with disease state and directly demonstrated an impact of this variation on serum bactericidal antibody activity (23). Genetic determinants of variance were not detected for our simplified, antibody-independent serum bactericidal antibody assay but were found for sensitivity to heat-inactivated serum possibly due to the absence of additional bovine serum albumin in our assay. This serendipitous observation indicated that inactivating mutations in capsular biosynthesis genes correlated with poor survival in this assay. Intriguingly, other minor *csw* alleles had unusual phenotypic attributes (Fig. S5). In the GWAS assay, nonsynonymous mutations in *sucA*, encoding a 2-oxoglutarate dehydrogenase E1 component, were associated with higher survival in AD-HI serum. These observations suggest that both major and more subtle changes in capsular synthesis impact meningococcal serum survival, possibly by increasing sensitivity to osmotic pressure or a heat-stable serum component, and that alterations in the Krebs cycle may buffer against the osmotic effects of serum and, by extrapolation, the bloodstream. An alternative possibility is that loss of the capsule increases aggregation and reduces the observed number of cfu. The effects of capsular alterations noted herein mirror previous findings showing that inactivation of meningococcal capsular genes or over-expression of capsule leads to alterations in resistance to the bactericidal effects of human serum (41, 42).

Our combined GWAS and PV analyses detected determinants of two meningococcal traits, biofilm formation and adhesion, critical for carriage, and putatively for transmissibility. Meningococcal lineages form biofilms through either extracellular DNA (eDNA)-dependent or -independent mechanisms (43). Positively charged surface proteins, such as *Neisseria* heparin-binding antigen (NhbA) and IgA protease, are thought to stabilize eDNA biofilm structures while NalP reduces biofilm formation by proteolytic cleavage of these proteins (44). Our results detected a correlation between lower biofilm formation and the NalP ON state (Fig. S16) and an association of the *nalP* repeat tract with biofilm variance in the unitig GWAS analysis. Arenas et al. (44) have previously shown that inactivation of *nalP* in a cc11 strain background results in higher biofilm formation. As NalP cleaves outer membrane proteins involved in DNA-binding, NalP PV may enable MenW:cc11 isolates to switch between different modes of biofilm formation resulting in access to the “spreader” trait attributed to eDNA-independent meningococcal lineages (43). A marked difference in adhesion was also observed with the disease isolates having lower levels of adherence to A549 cells than carriage isolates. This difference was linked to a nonsynonymous substitution in *pilC2* in the unitig GWAS. Another potential explanation is the high levels of both *pilC1* and *pilC2* ON states among carriage isolates that may underpin the group effect detected for adhesion in the PV analyses (Table 2). PilC proteins have several non-redundant functions including antagonizing Type IV pilus retraction and variable evidence of adhesion to specific ligands and cell types (45–47). This analysis does, however, have a major defect as poor assembly of long repeat tracts and paralogous genes prevented the inclusion of contributions from Opa proteins, the major meningococcal adhesions. While observed adhesion differences reflect an important distinction between carriage and disease isolates, these findings are biased by an inherent inability to sample the microvasculature where meningococcal pilus-dependent aggregates accumulate during the establishment of disease as opposed to circulating bacteria where loss of surface-pilus expression is potentially advantageous (45, 48).

One key outcome of our study was predictions of determinants of disease potential. Our RFE analysis indicates that three traits provide >80% accuracy in predicting the disease and carriage states (Fig. 6). Only the *fetA* PV state was a genetic element while the others were phenotypic states. However, AD-HI phenotypic variation is linked to the *csw* gene sequence suggesting some potential for a high-throughput genotypic prediction assay. Identification of genetic determinants of LDH phenotypic variance would enhance the potential for genotypic prediction of disease ability. Our disease potential analysis may also have detected a shift in the disease-causing ability of this lineage. This viewpoint arises from a post hoc analysis of the *fetA* promoter in invasive UK MenW:cc11 isolates obtained after our study where changes in these sequences, similar to those described herein for carriage isolates, were detected at high prevalence (i.e., a shift from a 97% to 78% prevalence of the 6C *fetA* repeat tract in pre-2017 and 2017–2024 invasive isolates, respectively; data not shown). Thus, the Nottingham carriage isolates may have contained a novel disease-causing sub-lineage that was beginning to circulate among carriers. This post hoc analysis reflects some other potential caveats arising from biases in our isolated collections. The invasive isolates were obtained mainly from infants and multiple locations across the UK and hence are likely representative of UK disease-causing strains of the MenW:cc11 lineage. The carriage isolates were all obtained from one carriage study at the University of Nottingham. As discussed elsewhere (49), incoming Nottingham students are from geographically dispersed locations, so partially representative of the UK carriage of this lineage. However, significant clonal expansion of the MenW:cc11 lineage was observed in this carriage study and this expansion is partially responsible for the clustering of most of the 2013 carriage isolates on the phylogenetic tree [(16); Fig. 1A]. These caveats of sub-lineages and isolate sources may impair the generalization of findings from our study. Testing of additional sets of carriage and disease isolates is therefore required to confirm how representative our data is of general phenomena and whether shifts in disease-associated phenotypes or genotypes are occurring over time.

Our study has detected associations between several genetic factors and phenotypic variation. Other researchers have previously demonstrated that inactivation of capsule and *nalP* genes produced outcomes similar to those observed herein (41, 42, 44). While validation by molecular manipulation and testing of MenW:cc11 strains is required, our study has identified multiple targets that can be explored in experimentally tractable assays. Another major limitation of our study was the use of Illumina technologies in generating genome assemblies. This approach limited our GWAS and PV association tests by compromising the assembly of functionally important multi-copy genes (e.g., *pilS*, *pilC*, *opa,* and *maf* loci) and IS elements. Mitigation of these problems requires the implementation of long-read sequencing technologies to improve genome assemblies. Low numbers of isolates and monophyletic distributions may also have reduced detection of significant variants but this is only addressable through automation of phenotypic assays. Despite these limitations, we have shown that multi-assay phenotype-to-genotype studies can detect genetic determinants of disease-associated phenotypes and predictors of disease potential for a major bacterial pathogen.

## MATERIALS AND METHODS

### Meningococcal isolates and genome sequences

Carriage isolates were obtained from a 2015–2016 carriage survey performed at Nottingham University (49). Disease isolates, provided by the Meningococcal Reference Unit, were selected from the 2010–2011 and 2015–2016 epidemiological (July to June inclusive) year groups. Assembled whole-genome sequences are available through the PubMLST *Neisseria* database (17); disease isolates form part of the Meningitis Research Foundation Meningococcus Genome Library (Data File S6). All isolates were routinely grown at 37°C, 5% CO_2_ on BHI agar supplemented with 5% horse blood (Sigma Aldrich, UK).

### Phenotypic assays

Details of phenotypic assays and raw data are provided in Data Files S7 and S8. Briefly, multi-assay stock plates were prepared, as described by Farzand et al. (25), by arraying bacterial isolates into batches of multiple 96-well polypropylene non-pyrogenic plates (Corning Incorporated Coaster) from single, mid-log phase cultures of isolates grown to mid-exponential phase in BHI (3–3.5 h). Stock plates were utilized as starting cultures for each assay. Growth assays were performed in BHI or RPMI 1640 media (Gibco, USA) by diluting overnight cultures 1:10 into relevant media in clear, flat-bottomed 96-well microtiter plates (Nunc, USA). Plates, sealed with breath-easy membranes, were incubated with shaking at 37°C, 5% CO_2_ in a BMG Labtech Omega FLUOstar plate reader. Logistic function curves were fitted to OD_600_ data for calculation of doubling time (r), lag phase (time to mid-log phase, Tmid), and maximum growth (k). Biofilm assays were performed in 96-peg lid plates (see reference 50) by diluting overnight cultures 1:3 into 100 µL tryptic soy broth medium and incubating statically for 24 h before staining the pegs with 1% crystal violet (51). Crystal violet recovered from pegs was quantified at OD_550_ and converted into % values relative to strain B141. Released LDH was measured using bacterial suspensions of mid-exponential phase cultures in RPMI 1640 media and a cytotoxicity detection kit (Sigma-Aldrich). Released LDH activity was reported as percentages of total LDH activity [obtained by treating bacterial suspensions with 1% (vol/vol) Triton X-100]. Adhesion to semi-confluent A594 (ATCC number: CCL185TM) cells, grown in RPMI-1640/10% FBS, was performed at a multiplicity of infection of 30. Cells were incubated with bacteria for 1 h at 37°C, 5% CO_2_, washed with phosphate-buffered saline (PBS) to remove non-adherent bacteria, and incubated for 18 h at 37°C, 5% CO_2_. Adherent bacterial cells, obtained by lysing infected monolayers with 0.1% saponin (Sigma, USA), were quantified by plating serial dilutions onto BHI agar plates. SBAD assays were performed with a 30% dilution of IgG/IgM-depleted human sera (Pel-Freeze 34010-5 HU Comp IgG/IgM Depleted Pooled) and AD-HI serum sensitivity assays with the same sera, and concentration, inactivated by heating to 56°C for 30 min. Bacterial suspensions, containing 10^4^ CFU diluted in 1× Hanks’ balanced salt solution, were mixed with serum and incubated for 1 h (T1) at 37°C, 5% CO_2_. Following the enumeration of colony-forming units, SBAD activity was calculated as the percentage reduction at T1 for active versus HI AD-serum and for AD-HI serum sensitivity as the percentage reduction relative to the inoculum.

### PV analysis

Repeat numbers were obtained either from published bioinformatic analyses of genome sequence data or by analysis with Phasomelt (52, 53). Reassembly of incomplete repeat tracts was performed by mapping raw FASTQ reads to the M25419 reference genome, using BWA and a conserved 1 kb trap sequence adjacent to the repeat tract, followed by assembly of SSR-specific reads with SPAdes (54, 55) and manually assessing repeat number in BioEdit (56). Published multiplex PCR and GeneScan analysis protocols were utilized to confirm repeat numbers for seven genes (i.e., *porA*, *fetA*, *nadA*, *hpuA*, *hmbR*, *pilC1,* and *pilC2*) and to generate additional data for *modA* and *modB* (53) (provided in the Leicester Data Repository). Briefly, PCR products, generated using fluorescently labeled primers spanning the repeat tract, were subject to electrophoresis on an autosequencer followed by conversion of PCR product sizes into repeat numbers in comparison to products of known repeat number. Expression states were assigned based on repeat number, position of SSR (i.e., ON/OFF for translational SSR and HIGH/INTERMEDIATE/LOW for transcriptional SSR), and published data (53, 57).

### Pan-genome and phylogenetic analysis

Velvet *de novo* assemblies for the 163 isolates were downloaded from the PubMLST *Neisseria* database (http://pubmlst.org/neisseria) and Illumina sequencing reads from the European Nucleotide Archive (ENA) database (https://www.ebi.ac.uk/ena). Genomes were annotated in a reference pan-genome approach using *N. meningitidis* strains M25419 (CP016678.1), DE10444 (CP012392.1), MC58 (AE002098.2) Z2491 (AL157959.1), FAM18 (AM421808.1), and annotations from the 163 genomes. From 904,711 open reading frames, a total of 4,446 unique genes were produced by identifying homologs of >70% sequence identity using BLAST and subject to automated annotation with RAST (58). Whole-genome multiple sequence alignments were produced by aligning orthologs in a gene-by-gene manner (59) with MAFFT (60). Matches of individual genes to the reference pan-genome list were detected using BLAST at >70% identity/>50% coverage. Core genes were shared by >90% of isolates. Phylogenetic trees were reconstructed, based on core gene-by-gene alignments, using the maximum-likelihood (ML) algorithm in RAxML v8.2.11 with GTRGAMMA as a substitution model (61). ClonalFrameML was used to account for recombination (61).

### Genome-wide association studies

GWAS analyses were conducted using pyseer (version 1.3.6) (62). For SNPs, variants were called relative to the M25419 genome with snippy (https://github.com/tseemann/snippy). To remove k-mer counting redundancies (63), unitigs were called with the unitig-caller package. Phenotype associations were tested, using continuous data derived from the 163 isolates, with FaST-LMM, a linear mixed model of fixed and random effects. A high-quality phylogeny, reconstructed with ClonalFrameML (61), was used to calculate similarities based on shared branch lengths between each pair’s MRCA with the inclusion of a root to correct for population structure. Significance thresholds were calculated from unique variant patterns with Bonferroni correction to control for multiple tests. Significant unitigs were mapped using BWA-MEM (version 0.7.17) (62). Manhattan plots were visualized with qqman package (64). Sequences for significant genetic variants were interrogated to ascribe variation to specific genes or IGR and to determine position relative to the M25419 reference sequence.

### Statistical analyses

Correlations between traits were studied with Spearman’s Rho correlation test. Both Kruskal-Wallis Rank Sum and Wilcoxon Rank Sum tests were performed to detect significant differences between groups (i.e., carriage, invasive_original, and invasive_2013) and PV states. Congruence tests between core genome and PV phylogenetic trees were performed with Analysis of Phylogenetics and Evolution version 5.7 package in R (65). The predictive power of phenotypic traits, PV genes, and phasotypes in allocating isolates to phenotypic variation, group, or disease state, was examined with RFE in a random forest model. Associations between PV genes, phasotypes, and phenotypes were tested using the Chi-square test of independence, Cramer’s V, and Type II/III ANOVA. All analyses were undertaken in the R software version 4.1.3.

## Data Availability

All data are either included within the manuscript or are openly available at the University of Leicester Research Repository, accessible *via* (66). For the purpose of open access, the author has applied a Creative Commons Attribution (CC BY) license to the Author Accepted Manuscript version arising from this submission.

## SUPPLEMENTAL MATERIAL

Data File S1 mbio.03059-24-s0001.xlsx PV data.

Data File S2 mbio.03059-24-s0002.xlsx Combined phenotype and PV data.

Data File S3 mbio.03059-24-s0003.xlsx MenW capsule alleles.

Data File S4 mbio.03059-24-s0004.xlsx SNP and unitig outputs of GWAS analysis.

Data File S5 mbio.03059-24-s0005.docx Statistical, regression, and random forest models.

Data File S6 mbio.03059-24-s0006.xlsx Isolates and metadata.

Data File S7 mbio.03059-24-s0007.docx Detailed methods.

Data File S8 mbio.03059-24-s0008.xlsx Raw phenotype data.

Supplemental Figures mbio.03059-24-s0009.docx Figures S1 to S29.

ASM does not own the copyrights to Supplemental Material that may be linked to, or accessed through, an article. The authors have granted ASM a non-exclusive, world-wide license to publish the Supplemental Material files. Please contact the corresponding author directly for reuse.
