# Supplementary material for: High-throughput phenotype-to-genotype testing of meningococcal carriage and disease isolates detects genetic determinants of disease-relevant phenotypic traits
Source: mBio. 2024 Oct 30;15(12):e03059-24. doi: 10.1128/mbio.03059-24 (PMC11633189; doi:10.1128/mbio.03059-24)
Supplement: Data File S5 — Statistical, regression, and random forest models. [file mbio.03059-24-s0005.docx]

**Statistical data analysis**

**Index of sections and list of tables and figures**.

- 1. Summary statistics for phenotype analyses. Pages 5-7.
  2. Non-parametric alternative to one-way ANOVA test. Page 8.
  3. Statistical analyses of distribution of phase variation (PV) states. Pages 8-9.
  4. Non-parametric method for detecting associations between phenotype and PV expression state. Pages 9-10.
  5. Type II/III ANOVA tests for detecting association of group and single gene PV states with phenotypic variance. Pages 11-19.
  6. Regression model for detecting association of group and all phase-variable genes with phenotypic variance. Pages 20-24.
  7. Phasotype groups and correlation testing. Page 25.
  8. Non-parametric method for detecting association between phasotypes and phenotypic variation. Pages 25-26.
  9. Type II/III ANOVA Type II/III ANOVA tests for detecting association of group and individual phasotype groups with phenotypic variance. Pages 26-31.
  10. Type II/III ANOVA Type II/III ANOVA tests for detecting association of group and all phasotype groups with phenotypic variance. Pages 31-34.
  11. Predictive models. Page 34.
      1. Feature Selection: Recursive feature elimination. Page 34-35.
      2. Group analyses with all phenotypes and PV genes/phasotypes. Page 35.
      3. Group analyses with phenotype variables only. Pages 35-36.
      4. Group analyses with PV genes and phasotypes only. Pages 36-37.
      5. Disease/carriage phenotype analyses. Pages 37-38.
      6. Phenotype predictions from group, phasotypes and PV genes. Pages 38-42.

**List of Tables**

Table 1.1.1. Summary statistics for each of the study phenotypes.

Table 1.2.1. Association between group and phenotypes

Table 1.4.1. Association between transcriptional PV genes and phenotypes.

Table 1.4.2. Association between translational PV genes and phenotypes.

Table 1.5.1. Analysis of variance in Adhesion contributed by group and/or PV genes

Table 1.5.2. Analysis of variance in Biofilms contributed by group and/or PV genes.

Table 1.5.3. Analysis of variance in BHI_k contributed by group and/or PV genes.

Table 1.5.4. Analysis of variance in BHI_r contributed by group and/or PV genes.

Table 1.5.5. Analysis of variance in BHI_tmid contributed by group and/or PV genes.

Table 1.5.6. Analysis of variance in RPMI_r contributed by group and/or PV genes.

Table 1.5.7. Analysis of variance in RPMI_k contributed by group and/or PV genes.

Table 1.5.8. Analysis of variance in RPMI_tmid contributed by group and/or PV genes.

Table 1.5.9. Analysis of variance in LDH contributed by group and/or PV genes.

Table 1.5.10. Analysis of variance in SBAD contributed by group and/or PV genes.

Table 1.5.11. Analysis of variance in AD.HI contributed by group and/or PV genes.

Table 1.6.1. Regression model: *Biofilms ~ group + porA + fetA + nadA + hpuA + nalP + mspA + hmbR + pilC1 + pilC2 + modA + modB*

Table 1.6.2. Regression model: *BHI_k ~ group + porA + fetA + nadA + hpuA + nalP + mspA + hmbR + pilC1 + pilC2 + modA + modB*

Table 1.6.3. Regression model: *BHI_r ~ group + porA + fetA + nadA + hpuA + nalP + mspA + hmbR + pilC1 + pilC2 + modA + modB*

Table 1.6.4. Regression model: *BHI_tmid ~ group + porA + fetA + nadA + hpuA + nalP + mspA + hmbR + pilC1 + pilC2 + modA + modB*

Table 1.6.5. Regression model: *RPMI_k ~ group + porA + fetA + nadA + hpuA + nalP + mspA + hmbR + pilC1 + pilC2 + modA + modB*

Table 1.6.6. Regression model: *RPMI_r ~ group + porA + fetA + nadA + hpuA + nalP + mspA + hmbR + pilC1 + pilC2 + modA + modB*

Table 1.6.7. Regression model: *RPMI_tmid ~ group + porA + fetA + nadA + hpuA + nalP + mspA + hmbR + pilC1 + pilC2 + modA + modB*

Table 1.6.8. Regression model: *LDH ~ group + porA + fetA + nadA + hpuA + nalP + mspA + hmbR + pilC1 + pilC2 + modA + modB*

Table 1.6.9. Regression model: *Adhesion ~ group + porA + fetA + nadA + hpuA + nalP + mspA + hmbR + pilC1 + pilC2 + modA + modB*

Table 1.6.10. Regression model: *SBAD ~ group + porA + fetA + nadA + hpuA + nalP + mspA + hmbR + pilC1 + pilC2 + modA + modB*

Table 1.6.11. Regression model: *AD.HI ~ group + porA + fetA + nadA + hpuA + nalP + mspA + hmbR + pilC1 + pilC2 + modA + modB*

Table 1.8.1. Association between phasotypes and phenotypes.

Table 1.9.1. Analysis of variance in Adhesion contributed by group and/or phasotypes.

Table 1.9.2. Analysis of variance in Biofilms contributed by group and/or phasotypes.

Table 1.9.3. Analysis of variance in BHI_k contributed by group and/or phasotypes.

Table 1.9.4. Analysis of variance in BHI_r contributed by group and/or phasotypes.

Table 1.9.5. Analysis of variance in BHI_tmid contributed by group and/or phasotypes.

Table 1.9.6. Analysis of variance in RPMI_k contributed by group and/or phasotypes.

Table 1.9.7. Analysis of variance in RPMI_r contributed by group and/or phasotypes.

Table 1.9.8. Analysis of variance in RPMI_tmid contributed by group and/or phasotypes.

Table 1.9.9. Analysis of variance in LDH contributed by group and/or phasotypes.

Table 1.9.10. Analysis of variance in SBAD contributed by group and/or phasotypes.

Table 1.9.11. Analysis of variance in AD.HI contributed by group and/or phasotypes.

Table 1.10.1. Regression model: *Biofilms ~ PILIN + AUTO + IRON + ADHESIN + MOD + group*

Table 1.10.2. Regression model: *BHI_k ~ PILIN + AUTO + IRON + ADHESIN + MOD + group*

Table 1.10.3. Regression model: *BHI_r ~ PILIN + AUTO + IRON + ADHESIN + MOD + group*

Table 1.10.4. Regression model: *BHI_tmid ~ PILIN + AUTO + IRON + ADHESIN + MOD + group*

Table 1.10.5. Regression model: *RPMI_k ~ PILIN + AUTO + IRON + ADHESIN + MOD + group*

Table 1.10.6. Regression model: *RPMI_r ~ PILIN + AUTO + IRON + ADHESIN + MOD + group*

Table 1.10.7. Regression model: *RPMI_tmid ~ PILIN + AUTO + IRON + ADHESIN + MOD + group*

Table 1.10.8. Regression model: *LDH ~ PILIN + AUTO + IRON + ADHESIN + MOD + group*

Table 1.10.9. Regression model: *Adhesion ~ PILIN + AUTO + IRON + ADHESIN + MOD + group*

Table 1.10.10. Regression model: *SBAD ~ PILIN + AUTO + IRON + ADHESIN + MOD + group*

Table 1.10.11. Regression model: *AD.HI ~ PILIN + AUTO + IRON + ADHESIN + MOD + group*

**List of Figures**

Figure 1.1.1. Density plots to view the distribution of each of the study phenotypes

Figure 1.11.1. Phenotypes identified in predicting group

Figure 1.11.2. Phasotypes and PV genes identified in predicting group

Figure 1.11.3. Phenotypes, phasotypes and/or PV genes identified in predicting whether an isolate is a disease or carriage isolate.

Figure 1.11.4. Group, phasotypes and PV genes identified in predicting BHI_tmid phenotype.

Figure 1.11.5. Group and PV genes identified in predicting RPMI_r phenotype.

Figure 1.11.6. Group and PV genes identified in predicting RPMI_tmid phenotype.

- 1. **Summary statistics for phenotype analyses**

| We used plots and summary statistics to explore the data. Table 1.1.1. shows the summary statistics for each set of phenotypic data. We visualised the phenotype data using density plots. Figure 1.1.1 shows data plots that indicate the data is highly skewed. Since this violates the assumptions of normality, we decided to use non-parametric methods for statistical analysis. To measure the strength of association between the phenotypes, we used Spearman’s Rho correlation test (see Figure Ax). The correlation coefficient is interpreted as: positive(+); negative(-); perfect(1); very strong(0.8 -0.9); moderate(0.6 - 0.7); fair(0.3 - 0.5);poor(0.1 - 0.2); none (0). The results show that the phenotypes are independent of each other.  Table 1.1.1. Summary statistics for each of the study phenotypes. |
| --- |
| \| **Phenotype** \| **Min** \| **1^st^ Quartile** \| **Median** \| **Mean** \| **3^rd^ Quartile** \| **Max** \| \| --- \| --- \| --- \| --- \| --- \| --- \| --- \| \| Biofilm \| -0.0025 \| 0.071 \| 0.1232 \| 0.2048 \| 0.2194 \| 1.884 \| \| BHI_k \| 0.3232 \| 0.6589 \| 0.7142 \| 0.7209 \| 0.7647 \| 1.545 \| \| BHI_r \| 0.1364 \| 0.244 \| 0.2719 \| 0.2842 \| 0.3028 \| 1.138 \| \| BHI_tmid \| 6.363 \| 8.558 \| 9.518 \| 10.01 \| 10.62 \| 24.04 \| \| RPMI_k \| 0.0662 \| 0.2238 \| 0.296 \| 0.3026 \| 0.3615 \| 1.1 \| \| RPMI_r \| 0.0476 \| 0.2456 \| 0.2943 \| 0.3756 \| 0.3915 \| 1.51 \| \| RPMI_tmid \| 0.6485 \| 3.846 \| 5.762 \| 6.488 \| 7.191 \| 51.22 \| \| LDH \| 4.198 \| 33.43 \| 48.65 \| 45.96 \| 57.11 \| 93.03 \| \| Adhesion \| 750 \| 1808333 \| 5166667 \| 80785775 \| 50833333 \| 633333333 \| \| SBAD \| 14.3 \| 58.72 \| 71.98 \| 69.93 \| 86.29 \| 97.99 \| \| AD.HI \| 0.001 \| 0.2119 \| 0.4693 \| 0.8674 \| 1.274 \| 4.939 \| |


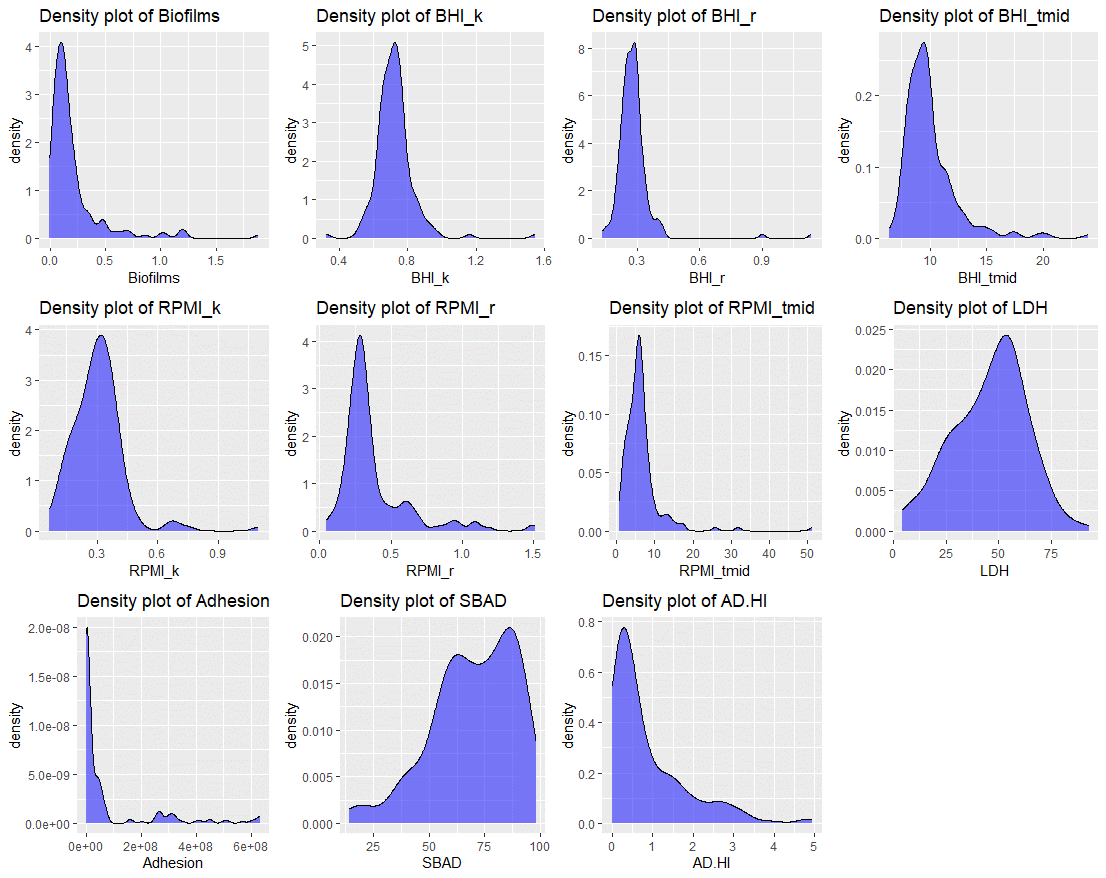
 ***Figure 1.1.1. Density plots to view the distribution of each of the study phenotypes.***

**1.2 Non-parametric alternative to one-way ANOVA test**

We used the Kruskal-Wallis rank sum method for testing whether samples originated from the same distribution. The null hypothesis of the Kruskal-Wallis test is that the mean ranks of the groups are the same. A Wilcoxon Rank Sum test for two independent samples (also known as Mann-Whitney U test) was then used to perform pairwise comparisons of phenotype means between the different groups. Results from these analyses are summarised in the table below (also visualised in Figure 3 in the manuscript). Note, we only performed Wilcoxon test for phenotypes that had significant differences (bold) in the Kruskal-Wallis test.

Table 1.2.1. Association between group and phenotypes.

| **Phenotype** | **Kruskal-Wallis rank sum test** | | | **Wilcoxon rank sum test** | |
| --- | --- | --- | --- | --- | --- |
|  | **Chi-square** | **DF** | **p-value** | **Comparisons** | **p-value** |
| Biofilm | 4.259 | 2 | 0.1189 | NA |  |
| BHI_k | 0.3405 | 2 | 0.8434 | NA |  |
| BHI_r | 22.58 | 2 | **1.247e-05** | Carriage vs Invasive Original | **1.6e-05** |
|  |  |  |  | Carriage vs Invasive 2013 | **0.0018** |
|  |  |  |  | Invasive Original vs Invasive 2013 | 0.1271 |
| BHI_tmid | 30.57 | 2 | **2.328e-07** | Carriage vs Invasive Original | **3.1e-07** |
|  |  |  |  | Carriage vs Invasive 2013 | **0.0001** |
|  |  |  |  | Invasive Original vs Invasive 2013 | 0.2404 |
| RPMI_k | 16.11 | 2 | **0.0003** | Carriage vs Invasive Original | **0.0011** |
|  |  |  |  | Carriage vs Invasive 2013 | **0.0012** |
|  |  |  |  | Invasive Original vs Invasive 2013 | 0.7001 |
| RPMI_r | 25.72 | 2 | **2.602e-06** | Carriage vs Invasive Original | 2.4e-05 |
|  |  |  |  | Carriage vs Invasive 2013 | **0.0015** |
|  |  |  |  | Invasive Original vs Invasive 2013 | **0.0034** |
| RPMI_tmid | 11.15 | 2 | **0.0038** | Carriage vs Invasive Original | **0.006** |
|  |  |  |  | Carriage vs Invasive 2013 | **0.017** |
|  |  |  |  | Invasive Original vs Invasive 2013 | 0.401 |
| LDH | 57.26 | 2 | **3.675e-13** | Carriage vs Invasive Original | **3.9e-12** |
|  |  |  |  | Carriage vs Invasive 2013 | **3.9e-09** |
|  |  |  |  | Invasive Original vs Invasive 2013 | 0.51 |
| Adhesion | 26.002 | 2 | **2.258e-06** | Carriage vs Invasive Original | **0.0003** |
|  |  |  |  | Carriage vs Invasive 2013 | **8.5e-06** |
|  |  |  |  | Invasive Original vs Invasive 2013 | 0.0664 |
| SBAD | 0.5145 | 2 | 0.7732 | NA |  |
| AD.HI | 60.58 | 2 | **7.017e-14** | Carriage vs Invasive Original | **3.6e-12** |
|  |  |  |  | Carriage vs Invasive 2013 | **3.3e-09** |
|  |  |  |  | Invasive Original vs Invasive 2013 | **0.036** |

**1.3 Statistical analyses of distribution of phase variation (PV) states**

The distribution of isolates among different PV expression states for the 11 phase-variable genes is shown in Figure A9 of the Supplementary Figures file. We used the Chi-square Test of Independence (Pearson’s chi-square test), for genes with two levels, or Cramer’s V, for genes with more than two levels, to study the correlation between the single genes and group (Figure A20 of Supplementary Figures file) as well as between the PV genes. Cramer’s V was used to calculate the correlation between the nominal categorical variables. The value for Cramer’s V ranges from 0 to 1, with 0 indicating no association between the variables and 1 indicating a strong association between the variables. To interpret Cramer’s V, we convert Cramér’s V to Cohen’s omega (ω) by multiplying V by the square root of the table’s degrees of freedom. The degrees of freedom being the contingency table’s smallest dimension minus 1. We then use the rule of thumb for interpreting ω, which is:

| **ω** | **Effect Size** |
| --- | --- |
| 0.10 | small |
| 0.30 | medium |
| 0.50 | large |

Most of the genes had weak associations except for moderate associations between *hmbR* and *fetA* (Cramer’s V = 0.29); *mspA* and *fetA* (Cramer’s V = 0.32). Associations were also observed between *hmbR* and *mspA* (χ^2^ _pearson’s_ = 20.32, p-value = 6.55e-06); *pilC1* and *hmbR* (χ^2^ _pearson’s_ = 8.87, p-value = 2.89e-03); *pilC1* and *pilC2* (χ^2^ _pearson’s_ = 6.19, p-value = 0.01).

**1.4 Non-parametric method for detecting associations between phenotype and PV expression state**

We used Kruskal-Wallis test to test for mean differences in phenotypes for the expression states in the transcriptional PV genes. Wilcoxon test was used to perform pairwise comparisons of phenotype means between different levels of the gene expression. Results for these tests are shown in the supplementary figures file: Figure A10 - A19 and tables 1.4.1 and 1.4.2 (divided into transcriptional PV genes and translational PV genes, respectively). Note, for transcriptional genes, Wilcoxon test was only performed for phenotypes that had significant differences (bold) in the Kruskal-Wallis test.

Transcriptional result summary (Table 1.4.1). Higher Biofilm formation was associated with low as compared to high expression of the *fetA* and *porA* genes. More time to reach mid-log growth in BHI, lower LDH activity and a lower survival in heat-inactivated serum relative to the total population were associated with high expression of *porA* as compared to both low and intermediate expression. A higher growth rate in RPMI was associated with high and low expression as compared to intermediate expression of the *porA* gene. Higher maximum growth in RPMI media and LDH activity were observed during high and low expression as compared to intermediate expression of the *nadA* gene. Survival in heat-inactivated serum relative to the total population was higher during low expression of the *nadA* gene compared to intermediate expression.

Translational result summary (Table 1.4.2). A lower growth rate and consequently more time to reach mid-log growth in BHI; a lower carrying capacity in RPMI; lower LDH activity; and a lower survival in heat-inactivated serum relative to the total population were observed when *pilC1* was in an ON expression state. There was also higher adhesion to A549 cells when *pilC1* was in an ON expression state. Lower biofilm formation was observed when *nalP* was in an ON expression state. Higher growth rate and consequently lower time to reach mid log phase in BHI was observed when *modA* was in an ON expression state.

Table 1.4.1. Association between transcriptional PV genes and phenotypes.

| **Phenotype** | **Kruskal-Wallis rank sum test** (**p-value)** | | | **Wilcoxon rank sum test** (**p-value)** | | | |
| --- | --- | --- | --- | --- | --- | --- | --- |
|  | **fetA** | **porA** | **nadA** | **Comparisons** | **fetA** | **porA** | **nadA** |
| Biofilm | **0.02** | **0.017** | 0.89 | Low vs Intermediate | 0.0553 | 0.1774 | NA |
|  |  |  |  | Low vs High | **0.0069** | **0.0047** |  |
|  |  |  |  | Intermediate vs High | 0.548 | 0.1445 |  |
| BHI_k | 0.97 | 0.78 | 0.22 | NA |  |  |  |
| BHI_r | 0.36 | 0.095 | 0.54 | NA |  |  |  |
| BHI_tmid | 0.56 | **0.004** | 0.5 | Low vs Intermediate | NA | 0.1306 | NA |
|  |  |  |  | Low vs High |  | **0.0022** |  |
|  |  |  |  | Intermediate vs High |  | **0.0308** |  |
| RPMI_k | 0.077 | 0.064 | **0.014** | Low vs Intermediate | NA | NA | **0.049** |
|  |  |  |  | Low vs High |  |  | 0.0573 |
|  |  |  |  | Intermediate vs High |  |  | **0.0046** |
| RPMI_r | 0.85 | **0.0042** | 0.4 | Low vs Intermediate | NA | **0.048** | NA |
|  |  |  |  | Low vs High |  | 0.097 |  |
|  |  |  |  | Intermediate vs High |  | **0.002** |  |
| RPMI_tmid | 0.3 | 0.4 | 0.17 | NA |  |  |  |
| LDH | 0.47 | **8e-05** | **0.046** | Low vs Intermediate | NA | 0.9756 | **0.038** |
|  |  |  |  | Low vs High |  | **0.0005** | 0.24 |
|  |  |  |  | Intermediate vs High |  | **0.0001** | **0.03** |
| Adhesion | 0.067 | 0.79 | 0.93 | NA |  |  |  |
| SBAD | 0.13 | 0.68 | 0.21 | NA |  |  |  |
| AD.HI | 0.21 | **1e-04** | **0.012** | Low vs Intermediate | NA | 0.1358 | **0.0035** |
|  |  |  |  | Low vs High |  | **0.0057** | 0.3953 |
|  |  |  |  | Intermediate vs High |  | **4.1e-05** | 0.0964 |

Table 1.4.2. Association between translational PV genes and phenotypes.

| **Phenotype** | **Wilcoxon rank sum test (p-value)** | | | | | | | |
| --- | --- | --- | --- | --- | --- | --- | --- | --- |
|  | **nalP** | **mspA** | **hmbR** | **hpuA** | **pilC1** | **pilC2** | **modA** | **modB** |
| Biofilm | **0.0007** | 0.87 | 0.4 | 0.59 | 0.83 | 0.78 | 0.15 | 0.83 |
| BHI_k | 0.22 | 0.33 | 0.26 | 0.82 | 0.6 | 0.4 | 0.79 | 0.82 |
| BHI_r | 0.22 | 0.81 | 0.099 | 0.38 | **0.00057** | 0.36 | **0.014** | 0.99 |
| BHI_tmid | 0.32 | 0.13 | 0.063 | 0.53 | **0.016** | 0.61 | **0.0054** | 0.064 |
| RPMI_k | 0.56 | 0.092 | 0.43 | 0.87 | **0.016** | 0.39 | 0.42 | 0.31 |
| RPMI_r | 0.48 | 0.77 | 0.88 | 0.69 | 0.19 | 0.094 | 0.74 | 0.71 |
| RPMI_tmid | 0.55 | 0.44 | 0.49 | 0.62 | 0.28 | 0.061 | 0.44 | 0.22 |
| LDH | 0.69 | 0.22 | 0.34 | 0.56 | **0.00013** | 0.16 | 0.33 | 0.13 |
| Adhesion | 0.53 | 0.14 | 0.8 | 0.11 | 0.048 | 0.65 | 0.99 | 0.23 |
| SBAD | 0.25 | 0.85 | 0.46 | 0.073 | 0.77 | 0.55 | 0.44 | 0.63 |
| AD.HI | 0.42 | 0.47 | 0.39 | 0.83 | **0.00069** | 0.65 | 0.37 | 0.77 |

**1.5 Type II/III ANOVA tests for detecting association of group and single gene PV states with phenotypic variance**

We used ANOVA, a statistical process for analysing the contributions of different factors to the amount of variance of each phenotype. Since our data is unbalanced, we either used type II or type III ANOVA depending on the significance of the interaction term. Type II tests for evidence of a main effect and then for each main effector under the assumption of no significant interaction (i.e. test for interaction first and, only if an interaction is not significant, continue with the analysis for main effects). Type III tests for the presence of a main effect and then for other main effects but with interaction. This approach is therefore valid in the presence of significant interactions. If there is indeed no interaction, then type II is statistically more powerful than type III. These results are presented in table 2 in the main manuscript with more details for each phenotypes in Tables 1.5.1 to 1.5.11. P-values in bold shows the factors (either group, PV gene, or interaction term) that were significant in explaining the variance in our phenotypes.

Table 1.5.1. Analysis of variance in Adhesion contributed by group and/or PV genes.

| **Model** | **Factors** | **Sum of squares** | **Degree of Freedom (DF)** | **F-statistic** | | **p-value** |
| --- | --- | --- | --- | --- | --- | --- |
| Adhesion ~ fetA + group + fetA:group | fetA | 5.65e+16 | 2 | 1.35 | 0.26 | |
|  | group | 4.32e+17 | 2 | 10.29 | **6.34e-05** | |
|  | fetA:group | 9.54e+15 | 1 | 0.45 | 0.50 | |
| Adhesion ~ nadA + group + nadA:group | nadA | 1.82e+16 | 2 | 0.43 | 0.65 | |
|  | group | 5.69e+17 | 2 | 13.39 | **4.36e-06** | |
|  | nadA:group | 7.15e+16 | 4 | 0.84 | 0.50 | |
| Adhesion ~ porA + group + porA:group | porA | 8.32e+15 | 2 | 0.20 | 0.82 | |
|  | group | 4.69e+17 | 2 | 11.01 | **3.41e-05** | |
|  | porA:group | 7.32e+16 | 4 | 0.86 | 0.49 | |
| Adhesion ~ mspA + group + mspA:group | mspA | 1.14e+16 | 1 | 0.54 | 0.46 | |
|  | group | 5.56e+17 | 2 | 13.19 | **5.08e-06** | |
|  | mspA:group | 4.20e+16 | 2 | 1.00 | 0.37 | |
| Adhesion ~ hpuA + group + hpuA:group | hpuA | 2.72e+16 | 1 | 1.29 | 0.26 | |
|  | group | 5.54e+17 | 2 | 13.15 | **5.25e-06** | |
|  | hpuA:group | 2.96e+16 | 2 | 0.70 | 0.50 | |
| Adhesion ~ hmbR + group + hmbR:group | hmbR | 2.79e+14 | 1 | 0.01 | 0.91 | |
|  | group | 5.51e+17 | 2 | 12.98 | **6.05e-06** | |
|  | hmbR:group | 2.96e+16 | 2 | 0.70 | 0.50 | |
| Adhesion ~ nalP + group + nalP:group | nalP | 1.96e+16 | 1 | 0.93 | 0.34 | |
|  | group | 5.37e+17 | 2 | 12.79 | **7.13e-06** | |
|  | nalP:group | 4.81e+16 | 2 | 1.15 | 0.32 | |
| Adhesion ~ pilC1 + group + pilC1:group | pilC1 | 1.59e+16 | 1 | 0.76 | 0.38 | |
|  | group | 3.77e+17 | 2 | 9.01 | **1.97e-04** | |
|  | pilC1:group | 5.99e+16 | 2 | 1.43 | 0.24 | |
| Adhesion ~ pilC2 + group + pilC2 | pilC2 | 1.21e+16 | 1 | 0.57 | 0.45 | |
|  | group | 5.58e+17 | 2 | 13.25 | **4.81e-06** | |
|  | pilC2:group | 4.41e+16 | 2 | 1.05 | 0.35 | |
| Adhesion ~ modA + group + modA:group | modA | 9.66e+14 | 1 | 0.05 | 0.83 | |
|  | group | 5.47e+17 | 2 | 13.01 | **5.93e-06** | |
|  | modA:group | 5.94e+16 | 2 | 1.41 | 0.25 | |
| Adhesion ~ modB + group + modB:group | modB | 5.48e+16 | 1 | 2.66 | 0.11 | |
|  | group | 5.75e+17 | 2 | 13.95 | **2.66e-06** | |
|  | modB:group | 6.99e+16 | 2 | 1.69 | 0.19 | |

Table 1.5.2. Analysis of variance in Biofilms contributed by group and/or PV genes.

| **Model** | **Factors** | **Sum of squares** | **DF** | **F-statistic** | **p-value** |
| --- | --- | --- | --- | --- | --- |
| Biofilms ~ fetA + group + fetA:group | fetA | 0.62 | 2 | 5.21 | **0.01** |
|  | group | 0.48 | 2 | 4.01 | **0.02** |
|  | fetA:group | 0.08 | 1 | 1.28 | 0.26 |
| Biofilms ~ nadA + group + nadA:group | nadA | 0.07 | 2 | 0.52 | 0.60 |
|  | group | 0.97 | 2 | 7.64 | **6.88e-04** |
|  | nadA:group | 0.24 | 4 | 0.93 | 0.45 |
| Biofilms ~ porA + group + porA:group | porA | 0.34 | 2 | 2.81 | 0.06 |
|  | group | 0.65 | 2 | 5.36 | **0.01** |
|  | porA:group | 0.33 | 4 | 1.37 | 0.25 |
| Biofilms ~ mspA + group + mspA:group | mspA | 0.05 | 1 | 0.84 | 0.36 |
|  | group | 0.90 | 2 | 7.17 | **1.05e-03** |
|  | mspA:group | 0.10 | 2 | 0.82 | 0.44 |
| Biofilms ~ hpuA + group + hpuA:group | hpuA | 0.01 | 1 | 0.21 | 0.65 |
|  | group | 0.93 | 2 | 7.37 | **8.69e-04** |
|  | hpuA:group | 0.19 | 2 | 1.49 | 0.23 |
| Biofilms ~ hmbR + group + hmbR:group | hmbR | 0.23 | 1 | 3.76 | 0.05 |
|  | group | 0.93 | 2 | 7.57 | **7.27e-04** |
|  | hmbR:group | 0.23 | 2 | 1.86 | 0.16 |
| Biofilms ~ nalP + group + nalP:group | nalP | 2.08 | 1 | 41.58 | **1.33e-09** |
|  | group | 1.69 | 2 | 16.83 | **2.39e-07** |
|  | nalP:group | 0.94 | 2 | 9.36 | **1.44e-04** |
| Biofilms ~ pilC1 + group + pilC1:group | pilC1 | 0.20 | 1 | 3.33 | 0.07 |
|  | group | 1.13 | 2 | 9.18 | **1.69e-04** |
|  | pilC1:group | 0.22 | 2 | 1.83 | 0.16 |
| Biofilms ~ pilC2 + group + pilC2 | pilC2 | 0.01 | 1 | 0.21 | 0.65 |
|  | group | 0.93 | 2 | 7.25 | **9.74e-04** |
|  | pilC2:group | 0.03 | 2 | 0.22 | 0.81 |
| Biofilms ~ modA + group + modA:group | modA | 0.01 | 1 | 0.21 | 0.64 |
|  | group | 0.91 | 2 | 7.15 | **1.06e-03** |
|  | modA:group | 0.08 | 2 | 0.64 | 0.53 |
| Biofilms ~ modB + group + modB:group | modB | 0.00 | 1 | 0.03 | 0.87 |
|  | group | 0.91 | 2 | 7.14 | **1.08e-03** |
|  | modB:group | 0.03 | 2 | 0.25 | 0.78 |

Table 1.5.3. Analysis of variance in BHI_k contributed by group and/or PV genes.

| **Model** | **Factors** | **Sum of squares** | **DF** | **F-statistic** | **p-value** |
| --- | --- | --- | --- | --- | --- |
| BHI_k ~ fetA + group + fetA:group | fetA | 0.01 | 2 | 0.46 | 0.64 |
|  | group | 0.04 | 2 | 1.60 | 0.20 |
|  | fetA:group | 0.02 | 1 | 1.34 | 0.25 |
| BHI_k ~ nadA + group + nadA:group | nadA | 0.04 | 2 | 1.39 | 0.25 |
|  | group | 0.05 | 2 | 1.78 | 0.17 |
|  | nadA:group | 0.10 | 4 | 1.96 | 0.10 |
| BHI_k ~ porA + group + porA:group | porA | 0.01 | 2 | 0.26 | 0.77 |
|  | group | 0.03 | 2 | 1.18 | 0.31 |
|  | porA:group | 0.02 | 4 | 0.35 | 0.84 |
| BHI_k ~ mspA + group + mspA:group | mspA | 0.02 | 1 | 1.30 | 0.26 |
|  | group | 0.03 | 2 | 1.31 | 0.27 |
|  | mspA:group | 0.03 | 2 | 1.04 | 0.35 |
| BHI_k ~ hpuA + group + hpuA:group | hpuA | 0.08 | 1 | 6.67 | **0.01** |
|  | group | 0.11 | 2 | 4.15 | **0.02** |
|  | hpuA:group | 0.08 | 2 | 3.25 | **0.04** |
| BHI_k ~ hmbR + group + hmbR:group | hmbR | 0.00 | 1 | 0.11 | 0.74 |
|  | group | 0.03 | 2 | 1.22 | 0.30 |
|  | hmbR:group | 0.00 | 2 | 0.06 | 0.94 |
| BHI_k ~ nalP + group + nalP:group | nalP | 0.03 | 1 | 2.38 | 0.13 |
|  | group | 0.03 | 2 | 1.11 | 0.33 |
|  | nalP:group | 0.01 | 2 | 0.20 | 0.82 |
| BHI_k ~ pilC1 + group + pilC1:group | pilC1 | 0.00 | 1 | 0.37 | 0.54 |
|  | group | 0.02 | 2 | 0.67 | 0.51 |
|  | pilC1:group | 0.01 | 2 | 0.33 | 0.72 |
| BHI_k ~ pilC2 + group + pilC2 | pilC2 | 0.02 | 1 | 1.25 | 0.27 |
|  | group | 0.02 | 2 | 0.80 | 0.45 |
|  | pilC2:group | 0.01 | 2 | 0.21 | 0.81 |
| BHI_k ~ modA + group + modA:group | modA | 0.00 | 1 | 0.20 | 0.65 |
|  | group | 0.03 | 2 | 1.19 | 0.31 |
|  | modA:group | 0.01 | 2 | 0.24 | 0.79 |
| BHI_k ~ modB + group + modB:group | modB | 0.00 | 1 | 0.00 | 0.95 |
|  | group | 0.03 | 2 | 1.25 | 0.29 |
|  | modB:group | 0.04 | 2 | 1.65 | 0.20 |

Table 1.5.4. Analysis of variance in BHI_r contributed by group and/or PV genes.

| **Model** | **Factors** | **Sum of squares** | **DF** | **F-statistic** | **p-value** |
| --- | --- | --- | --- | --- | --- |
| BHI_r ~ fetA + group + fetA:group | fetA | 0.001 | 2 | 0.06 | 0.95 |
|  | group | 0.08 | 2 | 4.32 | **0.01** |
|  | fetA:group | 0.00 | 1 | 0.41 | 0.52 |
| BHI_r ~ nadA + group + nadA:group | nadA | 0.00 | 2 | 0.21 | 0.81 |
|  | group | 0.08 | 2 | 4.57 | **0.01** |
|  | nadA:group | 0.05 | 4 | 1.26 | 0.29 |
| BHI_r ~ porA + group + porA:group | porA | 0.06 | 2 | 3.24 | **0.04** |
|  | group | 0.09 | 2 | 4.96 | **0.01** |
|  | porA:group | 0.02 | 4 | 0.60 | 0.67 |
| BHI_r ~ mspA + group + mspA:group | mspA | 0.001 | 1 | 0.08 | 0.78 |
|  | group | 0.09 | 2 | 4.75 | **0.01** |
|  | mspA:group | 0.01 | 2 | 0.46 | 0.63 |
| BHI_r ~ hpuA + group + hpuA:group | hpuA | 0.02 | 1 | 2.24 | 0.14 |
|  | group | 0.09 | 2 | 4.95 | **0.01** |
|  | hpuA:group | 0.01 | 2 | 0.48 | 0.62 |
| BHI_r ~ hmbR + group + hmbR:group | hmbR | 0.001 | 1 | 0.11 | 0.74 |
|  | group | 0.09 | 2 | 4.78 | **0.01** |
|  | hmbR:group | 0.01 | 2 | 0.28 | 0.75 |
| BHI_r ~ nalP + group + nalP:group | nalP | 0.01 | 1 | 1.24 | 0.27 |
|  | group | 0.08 | 2 | 4.59 | **0.01** |
|  | nalP:group | 0.01 | 2 | 0.36 | 0.70 |
| BHI_r ~ pilC1 + group + pilC1:group | pilC1 | 0.01 | 1 | 1.58 | 0.21 |
|  | group | 0.05 | 2 | 2.53 | 0.08 |
|  | pilC1:group | 0.01 | 2 | 0.44 | 0.64 |
| BHI_r ~ pilC2 + group + pilC2 | pilC2 | 0.003 | 1 | 0.33 | 0.56 |
|  | group | 0.08 | 2 | 4.18 | **0.02** |
|  | pilC2:group | 0.004 | 2 | 0.24 | 0.79 |
| BHI_r ~ modA + group + modA:group | modA | 0.003 | 1 | 0.29 | 0.59 |
|  | group | 0.09 | 2 | 4.72 | **0.01** |
|  | modA:group | 0.01 | 2 | 0.79 | 0.46 |
| BHI_r ~ modB + group + modB:group | modB | 0.003 | 1 | 0.30 | 0.59 |
|  | group | 0.09 | 2 | 4.89 | **0.01** |
|  | modB:group | 0.01 | 2 | 0.42 | 0.66 |

Table 1.5.5. Analysis of variance in BHI_tmid contributed by group and/or PV genes.

| **Model** | **Factors** | **Sum of squares** | **DF** | **F-statistic** | **p-value** |
| --- | --- | --- | --- | --- | --- |
| BHI_tmid ~ fetA + group + fetA:group | fetA | 52.37 | 2 | 5.45 | **0.01** |
|  | group | 203.66 | 2 | 21.18 | **7.16e-09** |
|  | fetA:group | 3.52 | 1 | 0.73 | 0.39 |
| BHI_tmid ~ nadA + group + nadA:group | nadA | 15.06 | 2 | 1.51 | 0.22 |
|  | group | 169.97 | 2 | 17.07 | **2.01e-07** |
|  | nadA:group | 28.95 | 4 | 1.45 | 0.22 |
| BHI_tmid ~ porA + group + porA:group | porA | 16.81 | 2 | 1.66 | 0.19 |
|  | group | 116.24 | 2 | 11.48 | **2.25e-05** |
|  | porA:group | 14.26 | 4 | 0.70 | 0.59 |
| BHI_tmid ~ mspA + group + mspA:group | mspA | 49.2 | 1 | 10.17 | **0.00** |
|  | group | 196.1 | 2 | 20.29 | **1.45e-08** |
|  | mspA:group | 41.8 | 2 | 4.32 | **0.01** |
| BHI_tmid ~ hpuA + group + hpuA:group | hpuA | 0.24 | 1 | 0.05 | 0.83 |
|  | group | 156.44 | 2 | 15.26 | **8.80e-07** |
|  | hpuA:group | 5.46 | 2 | 0.53 | 0.59 |
| BHI_tmid ~ hmbR + group + hmbR:group | hmbR | 3.8 | 1 | 0.76 | 0.38 |
|  | group | 155.38 | 2 | 15.55 | **6.87e-07** |
|  | hmbR:group | 22.58 | 2 | 2.26 | 0.11 |
| BHI_tmid ~ nalP + group + nalP:group | nalP | 2.91 | 1 | 0.57 | 0.45 |
|  | group | 152.87 | 2 | 14.86 | **1.23e-06** |
|  | nalP:group | 0.25 | 2 | 0.02 | 0.98 |
| BHI_tmid ~ pilC1 + group + pilC1:group | pilC1 | 27.64 | 1 | 5.59 | **0.02** |
|  | group | 30.02 | 2 | 3.04 | 0.05 |
|  | pilC1:group | 31.41 | 2 | 3.18 | **0.04** |
| BHI_tmid ~ pilC2 + group + pilC2 | pilC2 | 26.16 | 1 | 5.29 | **0.02** |
|  | group | 23.06 | 2 | 2.33 | 0.10 |
|  | pilC2:group | 32.57 | 2 | 3.29 | **0.04** |
| BHI_tmid ~ modA + group + modA:group | modA | 29.64 | 1 | 6.15 | **0.01** |
|  | group | 149.03 | 2 | 15.45 | **7.48e-07** |
|  | modA:group | 23.82 | 2 | 2.47 | 0.09 |
| BHI_tmid ~ modB + group + modB:group | modB | 15 | 1 | 3.01 | 0.08 |
|  | group | 150.87 | 2 | 15.13 | **9.84e-07** |
|  | modB:group | 12.55 | 2 | 1.26 | 0.29 |

Table 1.5.6. Analysis of variance in RPMI_r contributed by group and/or PV genes.

| **Model** | **Factors** | **Sum of squares** | **DF** | **F-statistic** | **p-value** |
| --- | --- | --- | --- | --- | --- |
| RPMI_r ~ fetA + group + fetA:group | fetA | 0.51 | 2 | 5.34 | **0.01** |
|  | group | 2.01 | 2 | 20.93 | **8.73e-09** |
|  | fetA:group | 0.03 | 1 | 0.69 | 0.41 |
| RPMI_r ~ nadA + group + nadA:group | nadA | 0.07 | 2 | 0.73 | 0.49 |
|  | group | 1.40 | 2 | 13.67 | **3.44e-06** |
|  | nadA:group | 0.12 | 4 | 0.58 | 0.68 |
| RPMI_r ~ porA + group + porA:group | porA | 0.06 | 2 | 0.59 | 0.56 |
|  | group | 1.11 | 2 | 10.88 | **3.81e-05** |
|  | porA:group | 0.18 | 4 | 0.88 | 0.48 |
| RPMI_r ~ mspA + group + mspA:group | mspA | 0.13 | 1 | 2.71 | 0.10 |
|  | group | 1.56 | 2 | 15.99 | **4.76e-07** |
|  | mspA:group | 0.29 | 2 | 2.95 | 0.06 |
| RPMI_r ~ hpuA + group + hpuA:group | hpuA | 0.05 | 1 | 0.96 | 0.33 |
|  | group | 1.51 | 2 | 15.01 | **1.08e-06** |
|  | hpuA:group | 0.12 | 2 | 1.22 | 0.30 |
| RPMI_r ~ hmbR + group + hmbR:group | hmbR | 0.02 | 1 | 0.46 | 0.50 |
|  | group | 1.52 | 2 | 15.35 | **8.17e-07** |
|  | hmbR:group | 0.26 | 2 | 2.66 | 0.07 |
| RPMI_r ~ nalP + group + nalP:group | nalP | 0.003 | 1 | 0.07 | 0.80 |
|  | group | 1.51 | 2 | 14.74 | **1.36e-06** |
|  | nalP:group | 0.03 | 2 | 0.27 | 0.77 |
| RPMI_r ~ pilC1 + group + pilC1:group | pilC1 | 0.03 | 1 | 0.56 | 0.46 |
|  | group | 1.08 | 2 | 10.90 | **3.69e-05** |
|  | pilC1:group | 0.26 | 2 | 2.63 | 0.08 |
| RPMI_r ~ pilC2 + group + pilC2 | pilC2 | 0.01 | 1 | 0.12 | 0.73 |
|  | group | 1.42 | 2 | 13.90 | **2.76e-06** |
|  | pilC2:group | 0.06 | 2 | 0.60 | 0.55 |
| RPMI_r ~ modA + group + modA:group | modA | 0.001 | 1 | 0.01 | 0.92 |
|  | group | 1.52 | 2 | 14.94 | **1.15e-06** |
|  | modA:group | 0.10 | 2 | 0.96 | 0.38 |
| RPMI_r ~ modB + group + modB:group | modB | 0.04 | 1 | 0.84 | 0.36 |
|  | group | 1.49 | 2 | 14.71 | **1.40e-06** |
|  | modB:group | 0.09 | 2 | 0.89 | 0.41 |

Table 1.5.7. Analysis of variance in RPMI_k contributed by group and/or PV genes.

| **Model** | **Factors** | **Sum of squares** | **DF** | **F-statistic** | **p-value** |
| --- | --- | --- | --- | --- | --- |
| RPMI_k ~ fetA + group + fetA:group | fetA | 0.31 | 2 | 10.86 | **3.84e-05** |
|  | group | 0.23 | 2 | 8.12 | **4.42e-04** |
|  | fetA:group | 2.59e-03 | 1 | 0.18 | 0.67 |
| RPMI_k ~ nadA + group + nadA:group | nadA | 0.06 | 2 | 1.96 | 0.14 |
|  | group | 0.08 | 2 | 2.43 | 0.09 |
|  | nadA:group | 0.03 | 4 | 0.42 | 0.79 |
| RPMI_k ~ porA + group + porA:group | porA | 0.02 | 2 | 0.47 | 0.62 |
|  | group | 0.09 | 2 | 2.67 | 0.07 |
|  | porA:group | 0.06 | 4 | 0.89 | 0.47 |
| RPMI_k ~ mspA + group + mspA:group | mspA | 0.07 | 1 | 4.66 | 0.03 |
|  | group | 0.11 | 2 | 3.74 | **0.03** |
|  | mspA:group | 0.14 | 2 | 4.75 | 0.01 |
| RPMI_k ~ hpuA + group + hpuA:group | hpuA | 2.60e-03 | 1 | 0.16 | 0.69 |
|  | group | 0.11 | 2 | 3.28 | **0.04** |
|  | hpuA:group | 0.04 | 2 | 1.15 | 0.32 |
| RPMI_k ~ hmbR + group + hmbR:group | hmbR | 0.01 | 1 | 0.73 | 0.40 |
|  | group | 0.10 | 2 | 3.21 | **0.04** |
|  | hmbR:group | 0.02 | 2 | 0.71 | 0.49 |
| RPMI_k ~ nalP + group + nalP:group | nalP | 0.01 | 1 | 0.43 | 0.51 |
|  | group | 0.11 | 2 | 3.32 | **0.04** |
|  | nalP:group | 0.01 | 2 | 0.42 | 0.66 |
| RPMI_k ~ pilC1 + group + pilC1:group | pilC1 | 7.50e-04 | 1 | 0.05 | 0.83 |
|  | group | 0.08 | 2 | 2.42 | 0.09 |
|  | pilC1:group | 0.01 | 2 | 0.17 | 0.84 |
| RPMI_k ~ pilC2 + group + pilC2 | pilC2 | 9.80e-04 | 1 | 0.06 | 0.81 |
|  | group | 0.10 | 2 | 2.97 | 0.05 |
|  | pilC2:group | 8.80e-04 | 2 | 0.03 | 0.97 |
| RPMI_k ~ modA + group + modA:group | modA | 0.02 | 1 | 1.42 | 0.24 |
|  | group | 0.11 | 2 | 3.37 | **0.04** |
|  | modA:group | 3.00e-05 | 2 | 0.001 | 1.00 |
| RPMI_k ~ modB + group + modB:group | modB | 6.40e-04 | 1 | 0.04 | 0.84 |
|  | group | 0.10 | 2 | 3.19 | **0.04** |
|  | modB:group | 0.02 | 2 | 0.48 | 0.62 |

Table 1.5.8. Analysis of variance in RPMI_tmid contributed by group and/or PV genes.

| **Model** | **Factors** | **Sum of squares** | **DF** | **F-statistic** | **p-value** |
| --- | --- | --- | --- | --- | --- |
| RPMI_tmid ~ fetA + group + fetA:group | fetA | 80.2 | 2 | 1.40 | 0.25 |
|  | group | 28 | 2 | 0.49 | 0.62 |
|  | fetA:group | 15.2 | 1 | 0.53 | 0.47 |
| RPMI_tmid ~ nadA + group + nadA:group | nadA | 31.6 | 2 | 0.53 | 0.59 |
|  | group | 20 | 2 | 0.34 | 0.71 |
|  | nadA:group | 17.8 | 4 | 0.15 | 0.96 |
| RPMI_tmid ~ porA + group + porA:group | porA | 14.4 | 2 | 0.24 | 0.78 |
|  | group | 18.8 | 2 | 0.32 | 0.73 |
|  | porA:group | 38.1 | 4 | 0.32 | 0.86 |
| RPMI_tmid ~ mspA + group + mspA:group | mspA | 11.9 | 1 | 0.41 | 0.52 |
|  | group | 22 | 2 | 0.38 | 0.68 |
|  | mspA:group | 50.1 | 2 | 0.87 | 0.42 |
| RPMI_tmid ~ hpuA + group + hpuA:group | hpuA | 1.1 | 1 | 0.04 | 0.84 |
|  | group | 21.8 | 2 | 0.37 | 0.69 |
|  | hpuA:group | 2.3 | 2 | 0.04 | 0.96 |
| RPMI_tmid ~ hmbR + group + hmbR:group | hmbR | 0.3 | 1 | 0.01 | 0.91 |
|  | group | 20.7 | 2 | 0.35 | 0.70 |
|  | hmbR:group | 4.2 | 2 | 0.07 | 0.93 |
| RPMI_tmid ~ nalP + group + nalP:group | nalP | 6.2 | 1 | 0.21 | 0.65 |
|  | group | 21 | 2 | 0.36 | 0.70 |
|  | nalP:group | 40.2 | 2 | 0.69 | 0.50 |
| RPMI_tmid ~ pilC1 + group + pilC1:group | pilC1 | 6.2 | 1 | 0.22 | 0.64 |
|  | group | 23.8 | 2 | 0.41 | 0.66 |
|  | pilC1:group | 80.8 | 2 | 1.40 | 0.25 |
| RPMI_tmid ~ pilC2 + group + pilC2 | pilC2 | 1.4 | 1 | 0.05 | 0.83 |
|  | group | 22.5 | 2 | 0.39 | 0.68 |
|  | pilC2:group | 24.1 | 2 | 0.41 | 0.66 |
| RPMI_tmid ~ modA + group + modA:group | modA | 30 | 1 | 1.03 | 0.31 |
|  | group | 20.5 | 2 | 0.35 | 0.70 |
|  | modA:group | 0.1 | 2 | 0.001 | 0.99 |
| RPMI_tmid ~ modB + group + modB:group | modB | 0.2 | 1 | 0.01 | 0.93 |
|  | group | 21.4 | 2 | 0.36 | 0.70 |
|  | modB:group | 0.7 | 2 | 0.01 | 0.99 |

Table 1.5.9. Analysis of variance in LDH contributed by group and/or PV genes.

| **Model** | **Factors** | **Sum of squares** | **DF** | **F-statistic** | **p-value** |
| --- | --- | --- | --- | --- | --- |
| LDH ~ fetA + group + fetA:group | fetA | 1547 | 2 | 3.88 | **0.02** |
|  | group | 17114 | 2 | 42.96 | **1.32e-15** |
|  | fetA:group | 212 | 1 | 1.06 | 0.30 |
| LDH ~ nadA + group + nadA:group | nadA | 496 | 2 | 1.19 | 0.31 |
|  | group | 14558 | 2 | 34.79 | **3.41e-13** |
|  | nadA:group | 316 | 4 | 0.38 | 0.82 |
| LDH ~ porA + group + porA:group | porA | 1491.4 | 2 | 3.68 | **0.03** |
|  | group | 11400.5 | 2 | 28.13 | **3.85e-11** |
|  | porA:group | 339.8 | 4 | 0.42 | 0.79 |
| LDH ~ mspA + group + mspA:group | mspA | 692.5 | 1 | 3.51 | 0.06 |
|  | group | 16053.1 | 2 | 40.66 | **5.90e-15** |
|  | mspA:group | 1349 | 2 | 3.42 | **0.04** |
| LDH ~ hpuA + group + hpuA:group | hpuA | 294 | 1 | 1.45 | 0.23 |
|  | group | 15890 | 2 | 39.23 | **1.52e-14** |
|  | hpuA:group | 945 | 2 | 2.33 | 0.10 |
| LDH ~ hmbR + group + hmbR:group | hmbR | 217 | 1 | 1.07 | 0.30 |
|  | group | 15852 | 2 | 38.87 | **1.93e-14** |
|  | hmbR:group | 805 | 2 | 1.97 | 0.14 |
| LDH ~ nalP + group + nalP:group | nalP | 215 | 1 | 1.04 | 0.31 |
|  | group | 16002 | 2 | 38.61 | **2.31e-14** |
|  | nalP:group | 283 | 2 | 0.68 | 0.51 |
| LDH ~ pilC1 + group + pilC1:group | pilC1 | 238 | 1 | 1.15 | 0.29 |
|  | group | 11373 | 2 | 27.49 | **5.82e-11** |
|  | pilC1:group | 314 | 2 | 0.76 | 0.47 |
| LDH ~ pilC2 + group + pilC2 | pilC2 | 53 | 1 | 0.25 | 0.62 |
|  | group | 15384 | 2 | 36.63 | **8.82e-14** |
|  | pilC2:group | 8 | 2 | 0.02 | 0.98 |
| LDH ~ modA + group + modA:group | modA | 138 | 1 | 0.66 | 0.42 |
|  | group | 15639 | 2 | 37.45 | **5.03e-14** |
|  | modA:group | 117 | 2 | 0.28 | 0.76 |
| LDH ~ modB + group + modB:group | modB | 173 | 1 | 0.83 | 0.36 |
|  | group | 15497 | 2 | 37.24 | **5.80e-14** |
|  | modB:group | 195 | 2 | 0.47 | 0.63 |

Table 1.5.10. Analysis of variance in SBAD contributed by group and/or PV genes.

| **Model** | **Factors** | **Sum of squares** | **DF** | **F-statistic** | **p-value** |
| --- | --- | --- | --- | --- | --- |
| SBAD ~ fetA + group + fetA:group | fetA | 875 | 2 | 1.25 | 0.29 |
|  | group | 147 | 2 | 0.21 | 0.81 |
|  | fetA:group | 347 | 1 | 0.99 | 0.32 |
| SBAD ~ nadA + group + nadA:group | nadA | 473 | 2 | 0.68 | 0.51 |
|  | group | 441 | 2 | 0.64 | 0.53 |
|  | nadA:group | 2274 | 4 | 1.64 | 0.17 |
| SBAD ~ porA + group + porA:group | porA | 52 | 2 | 0.07 | 0.93 |
|  | group | 396 | 2 | 0.55 | 0.58 |
|  | porA:group | 1043 | 4 | 0.73 | 0.57 |
| SBAD ~ mspA + group + mspA:group | mspA | 1 | 1 | 0.00 | 0.96 |
|  | group | 516 | 2 | 0.73 | 0.49 |
|  | mspA:group | 263 | 2 | 0.37 | 0.69 |
| SBAD ~ hpuA + group + hpuA:group | hpuA | 902 | 1 | 2.60 | 0.11 |
|  | group | 126 | 2 | 0.18 | 0.83 |
|  | hpuA:group | 105 | 2 | 0.15 | 0.86 |
| SBAD ~ hmbR + group + hmbR:group | hmbR | 93 | 1 | 0.26 | 0.61 |
|  | group | 512 | 2 | 0.72 | 0.49 |
|  | hmbR:group | 484 | 2 | 0.68 | 0.51 |
| SBAD ~ nalP + group + nalP:group | nalP | 602 | 1 | 1.74 | 0.19 |
|  | group | 580 | 2 | 0.84 | 0.44 |
|  | nalP:group | 1124 | 2 | 1.62 | 0.20 |
| SBAD ~ pilC1 + group + pilC1:group | pilC1 | 3 | 1 | 0.01 | 0.92 |
|  | group | 457 | 2 | 0.66 | 0.52 |
|  | pilC1:group | 1817 | 2 | 2.63 | 0.08 |
| SBAD ~ pilC2 + group + pilC2 | pilC2 | 68 | 1 | 0.19 | 0.66 |
|  | group | 399 | 2 | 0.56 | 0.57 |
|  | pilC2:group | 195 | 2 | 0.27 | 0.76 |
| SBAD ~ modA + group + modA:group | modA | 189 | 1 | 0.54 | 0.46 |
|  | group | 485 | 2 | 0.69 | 0.50 |
|  | modA:group | 795 | 2 | 1.13 | 0.33 |
| SBAD ~ modB + group + modB:group | modB | 103 | 1 | 0.29 | 0.59 |
|  | group | 475 | 2 | 0.67 | 0.52 |
|  | modB:group | 162 | 2 | 0.23 | 0.80 |

Table 1.5.11. Analysis of variance in AD.HI contributed by group and/or PV genes.

| **Model** | **Factors** | **Sum of squares** | **DF** | **F-statistic** | **p-value** |
| --- | --- | --- | --- | --- | --- |
| AD.HI ~ fetA + group + fetA:group | fetA | 0.63 | 2 | 0.42 | 0.66 |
|  | group | 30.73 | 2 | 20.45 | **1.28e-08** |
|  | fetA:group | 0.21 | 1 | 0.28 | 0.60 |
| AD.HI ~ nadA + group + nadA:group | nadA | 1.12 | 2 | 0.74 | 0.48 |
|  | group | 29.31 | 2 | 19.36 | **3.15e-08** |
|  | nadA:group | 1.16 | 4 | 0.38 | 0.82 |
| AD.HI ~ porA + group + porA:group | porA | 1.21 | 2 | 0.82 | 0.44 |
|  | group | 20.83 | 2 | 14.15 | **2.28e-06** |
|  | porA:group | 4.27 | 4 | 1.45 | 0.22 |
| AD.HI ~ mspA + group + mspA:group | mspA | 0.65 | 1 | 0.87 | 0.35 |
|  | group | 32.55 | 2 | 21.77 | **4.51e-09** |
|  | mspA:group | 0.85 | 2 | 0.57 | 0.57 |
| AD.HI ~ hpuA + group + hpuA:group | hpuA | 0.69 | 1 | 0.93 | 0.34 |
|  | group | 31.68 | 2 | 21.30 | **6.53e-09** |
|  | hpuA:group | 1.41 | 2 | 0.95 | 0.39 |
| AD.HI ~ hmbR + group + hmbR:group | hmbR | 3.56 | 1 | 4.92 | **0.03** |
|  | group | 31.56 | 2 | 21.83 | **4.32e-09** |
|  | hmbR:group | 1.77 | 2 | 1.22 | 0.30 |
| AD.HI ~ nalP + group + nalP:group | nalP | 1.21 | 1 | 1.62 | 0.21 |
|  | group | 31.26 | 2 | 20.98 | **8.43e-09** |
|  | nalP:group | 0.65 | 2 | 0.44 | 0.65 |
| AD.HI ~ pilC1 + group + pilC1:group | pilC1 | 0.00 | 1 | 0.00 | 0.98 |
|  | group | 26.74 | 2 | 17.71 | **1.16e-07** |
|  | pilC1:group | 0.31 | 2 | 0.21 | 0.81 |
| AD.HI ~ pilC2 + group + pilC2 | pilC2 | 0.38 | 1 | 0.50 | 0.48 |
|  | group | 32.46 | 2 | 21.60 | **5.19e-09** |
|  | pilC2:group | 0.45 | 2 | 0.30 | 0.74 |
| AD.HI ~ modA + group + modA:group | modA | 1.11 | 1 | 1.50 | 0.22 |
|  | group | 31.55 | 2 | 21.29 | **6.59e-09** |
|  | modA:group | 1.40 | 2 | 0.94 | 0.39 |
| AD.HI ~ modB + group + modB:group | modB | 0.00 | 1 | 0.00 | 0.98 |
|  | group | 32.15 | 2 | 21.45 | **5.83e-0****9** |
|  | modB:group | 1.15 | 2 | 0.76 | 0.47 |

**1.6. Regression model for detecting association of group and all phase-variable genes with phenotypic variance**

We created regression models with all the phase-variable genes and group to identify the factors that are associated with our phenotypes (Results also in Table 2 in the manuscript). When the p-value is less than the significance level (p-value < 0.05), then the independent variable (phase-variable gene or group) is associated with changes in the dependent variable (phenotype). We went further to determine the coefficients for each of the independent variables. These coefficient estimates show whether there is a positive or negative correlation between each independent variable and the dependent variable. The coefficient estimates also indicates the average difference in the dependent variable between the reference category and the other categories within the independent variable while holding other variables in the model constant. For example, taking group as an independent variable with three categories: carriage, invasive original and invasive 2013, the model has chosen carriage as the reference. The coefficient estimates are therefore a comparison between carriage and invasive original and a comparison between carriage and invasive 2013.

Table 1.6.1. Regression model: *Biofilms ~ group + porA + fetA + nadA + hpuA + nalP + mspA + hmbR + pilC1 + pilC2 + modA + modB*

| **Factors** | **Sum of squares** | **DF** | **F-statistic** | **p-value** | **Coefficients** | | | | |
| --- | --- | --- | --- | --- | --- | --- | --- | --- | --- |
|  |  |  |  |  |  | **Estimate** | **Std. Error** | **t value** | **P-value** |
|  |  |  |  |  | (Intercept) | 0.55 | 0.09 | 5.90 | **2.44e-08** |
| **group** | 0.32 | 2 | 3.02 | 0.05 | group-Invasive_Original | -0.15 | 0.06 | -2.39 | **0.02** |
|  |  |  |  |  | group-Invasive_2013 | -0.11 | 0.06 | -1.89 | 0.06 |
| **porA** | 0.42 | 2 | 3.93 | **0.02** | porA-Intermediate | 0.10 | 0.06 | 1.81 | 0.07 |
|  |  |  |  |  | porA-High | 0.13 | 0.05 | 2.76 | **0.01** |
| **fetA** | 0.37 | 2 | 3.49 | **0.03** | fetA-Intermediate | -0.31 | 0.13 | -2.37 | **0.02** |
|  |  |  |  |  | fetA-High | -0.20 | 0.09 | -2.21 | **0.03** |
| **nadA** | 0.01 | 2 | 0.09 | 0.91 | nadA-Intermediate | -0.005 | 0.06 | -0.07 | 0.94 |
|  |  |  |  |  | nadA-High | 0.02 | 0.06 | 0.40 | 0.69 |
| **hpuA** | 0.03 | 1 | 0.59 | 0.45 | hpuA-ON | 0.03 | 0.04 | 0.77 | 0.45 |
| **nalP** | 0.83 | 1 | 15.63 | **0.0001** | nalP-ON | -0.16 | 0.04 | -3.95 | **1.19e-04** |
| **mspA** | 0.003 | 1 | 0.05 | 0.82 | mspA-ON | 0.01 | 0.05 | 0.23 | 0.82 |
| **hmbR** | 0.06 | 1 | 1.04 | 0.31 | HmbR-ON | -0.05 | 0.05 | -1.02 | 0.31 |
| **pilC1** | 0.06 | 1 | 1.15 | 0.29 | pilC1-ON | -0.05 | 0.05 | -1.07 | 0.29 |
| **pilC2** | 0.0001 | 1 | 0.001 | 0.97 | pilC2-ON | -0.002 | 0.04 | -0.04 | 0.97 |
| **modA** | 0.0003 | 1 | 0.005 | 0.95 | modA-ON | -0.003 | 0.05 | -0.07 | 0.95 |
| **modB** | 0.0001 | 1 | 0.002 | 0.96 | modB-ON | -0.002 | 0.05 | -0.05 | 0.96 |

Table 1.6.2. Regression model: *BHI_k ~ group + porA + fetA + nadA + hpuA + nalP + mspA + hmbR + pilC1 + pilC2 + modA + modB*

| Factors | Sum of squares | DF | F-statistic | p-value |
| --- | --- | --- | --- | --- |
| group | 0.02 | 2 | 0.93 | 0.40 |
| porA | 0.02 | 2 | 0.62 | 0.54 |
| fetA | 0.03 | 2 | 1.03 | 0.36 |
| nadA | 0.04 | 2 | 1.50 | 0.23 |
| hpuA | 0.02 | 1 | 1.58 | 0.21 |
| nalP | 0.04 | 1 | 3.02 | 0.08 |
| mspA | 0.01 | 1 | 0.79 | 0.37 |
| hmbR | 0.02 | 1 | 1.21 | 0.27 |
| pilC1 | 0.02 | 1 | 1.14 | 0.29 |
| pilC2 | 0.001 | 1 | 0.04 | 0.84 |
| modA | 0.01 | 1 | 0.45 | 0.50 |
| modB | 0.001 | 1 | 0.09 | 0.77 |

Table 1.6.3. Regression model: *BHI_r ~ group + porA + fetA + nadA + hpuA + nalP + mspA + hmbR + pilC1 + pilC2 + modA + modB*

| ***Factors*** | ***Sum of squares*** | ***DF*** | ***F-statistic*** | ***p-value*** |
| --- | --- | --- | --- | --- |
| group | 0.03 | 2 | 1.78 | 0.17 |
| porA | 0.05 | 2 | 2.68 | 0.07 |
| fetA | 0.001 | 2 | 0.07 | 0.94 |
| nadA | 0.01 | 2 | 0.45 | 0.64 |
| hpuA | 0.01 | 1 | 1.45 | 0.23 |
| nalP | 0.01 | 1 | 1.27 | 0.26 |
| mspA | 0.002 | 1 | 0.17 | 0.68 |
| hmbR | 2.00e-05 | 1 | 0.002 | 0.97 |
| pilC1 | 0.01 | 1 | 0.81 | 0.37 |
| pilC2 | 0.01 | 1 | 0.67 | 0.41 |
| modA | 1.00e-05 | 1 | 0.00 | 0.97 |
| modB | 0.01 | 1 | 0.67 | 0.41 |

Table 1.6.4. Regression model: *BHI_tmid ~ group + porA + fetA + nadA + hpuA + nalP + mspA + hmbR + pilC1 + pilC2 + modA + modB*

| **Factors** | **Sum of squares** | **DF** | **F-statistic** | **p-value** | **Coefficients** | | | | |
| --- | --- | --- | --- | --- | --- | --- | --- | --- | --- |
|  |  |  |  |  |  | **Estimate** | **Std. Error** | **t value** | **P-value** |
|  |  |  |  |  | (Intercept) | 10.77 | 0.88 | 12.23 | **2.00e-16** |
| group | 110.55 | 2 | 11.61 | **2.09e-05** | groupInvasive_Original | -2.81 | 0.60 | -4.70 | **6.10e-06** |
|  |  |  |  |  | groupInvasive_2013 | -1.99 | 0.54 | -3.69 | **3.21e-04** |
| porA | 4.45 | 2 | 0.47 | 0.63 | porAIntermediate | 0.32 | 0.53 | 0.61 | 0.54 |
|  |  |  |  |  | porAHigh | 0.44 | 0.46 | 0.95 | 0.34 |
| fetA | 32.08 | 2 | 3.37 | **0.04** | fetAIntermediate | -0.64 | 1.25 | -0.51 | 0.61 |
|  |  |  |  |  | fetAHigh | 1.68 | 0.87 | 1.94 | 0.05 |
| nadA | 10.01 | 2 | 1.05 | 0.35 | nadAIntermediate | -0.87 | 0.60 | -1.45 | 0.15 |
|  |  |  |  |  | nadAHigh | -0.09 | 0.55 | -0.16 | 0.88 |
| hpuA | 1.04 | 1 | 0.22 | 0.64 | hpuAON | -0.17 | 0.36 | -0.47 | 0.64 |
| nalP | 3.69 | 1 | 0.78 | 0.38 | nalPON | -0.34 | 0.39 | -0.88 | 0.38 |
| mspA | 0.29 | 1 | 0.06 | 0.80 | mspAON | -0.12 | 0.50 | -0.25 | 0.80 |
| hmbR | 0.26 | 1 | 0.05 | 0.82 | hmbRON | -0.10 | 0.44 | -0.23 | 0.82 |
| pilC1 | 0.47 | 1 | 0.10 | 0.75 | pilC1ON | 0.14 | 0.43 | 0.31 | 0.75 |
| pilC2 | 1.41 | 1 | 0.30 | 0.59 | pilC2ON | -0.21 | 0.39 | -0.54 | 0.59 |
| modA | 27.17 | 1 | 5.71 | **0.02** | modAON | -1.07 | 0.45 | -2.39 | **0.02** |
| modB | 10.99 | 1 | 2.31 | 0.13 | modBON | -0.68 | 0.45 | -1.52 | 0.13 |

Table 1.6.5. Regression model: *RPMI_k ~ group + porA + fetA + nadA + hpuA + nalP + mspA + hmbR + pilC1 + pilC2 + modA + modB*

| **Factors** | **Sum of squares** | **DF** | **F-statistic** | **p-value** | **Coefficients** | | | | |
| --- | --- | --- | --- | --- | --- | --- | --- | --- | --- |
|  |  |  |  |  |  | **Estimate** | **Std. Error** | **t value** | **P-value** |
|  |  |  |  |  | (Intercept) | 0.42 | 0.05 | 8.47 | **2.36e-14** |
| group | 0.18 | 2 | 6.09 | **0.003** | groupInvasive_Original | 0.11 | 0.03 | 3.44 | **0.001** |
|  |  |  |  |  | groupInvasive_2013 | 0.08 | 0.03 | 2.52 | **0.01** |
| porA | 0.02 | 2 | 0.68 | 0.51 | porAIntermediate | -0.03 | 0.03 | -1.14 | 0.26 |
|  |  |  |  |  | porAHigh | -0.01 | 0.03 | -0.33 | 0.74 |
| fetA | 0.22 | 2 | 7.49 | **0.001** | fetAIntermediate | -0.12 | 0.07 | -1.74 | 0.08 |
|  |  |  |  |  | fetAHigh | -0.19 | 0.05 | -3.86 | **1.68e-04** |
| nadA | 0.06 | 2 | 1.91 | 0.15 | nadAIntermediate | -0.05 | 0.03 | -1.37 | 0.17 |
|  |  |  |  |  | nadAHigh | 0.04 | 0.03 | 1.14 | 0.26 |
| hpuA | 1.00e-05 | 1 | 0.001 | 0.98 | hpuAON | 0.001 | 0.02 | 0.03 | 0.98 |
| nalP | 0.001 | 1 | 0.04 | 0.85 | nalPON | -0.004 | 0.02 | -0.20 | 0.85 |
| mspA | 0.02 | 1 | 1.03 | 0.31 | mspAON | 0.03 | 0.03 | 1.02 | 0.31 |
| hmbR | 0.01 | 1 | 0.59 | 0.44 | hmbRON | 0.02 | 0.02 | 0.77 | 0.44 |
| pilC1 | 0.01 | 1 | 0.59 | 0.45 | pilC1ON | 0.02 | 0.02 | 0.77 | 0.45 |
| pilC2 | 0.002 | 1 | 0.11 | 0.74 | pilC2ON | -0.01 | 0.02 | -0.33 | 0.74 |
| modA | 0.02 | 1 | 1.35 | 0.25 | modAON | -0.03 | 0.02 | -1.16 | 0.25 |
| modB | 0.00 | 1 | 0.27 | 0.61 | modBON | -0.01 | 0.02 | -0.52 | 0.61 |

Table 1.6.6. Regression model: *RPMI_r ~ group + porA + fetA + nadA + hpuA + nalP + mspA + hmbR + pilC1 + pilC2 + modA + modB*

| **Factors** | **Sum of squares** | **DF** | **F-statistic** | **p-value** | **Coefficients** | | | | |
| --- | --- | --- | --- | --- | --- | --- | --- | --- | --- |
|  |  |  |  |  |  | **Estimate** | **Std. Error** | **t value** | **P-value** |
|  |  |  |  |  | (Intercept) | 0.39 | 0.09 | 4.35 | **2.51e-05** |
| group | 1.04 | 2 | 10.34 | **0.0001** | groupInvasive_Original | -0.26 | 0.06 | -4.15 | **5.56e-05** |
|  |  |  |  |  | groupInvasive_2013 | -0.22 | 0.06 | -3.97 | **0.0001** |
| porA | 0.01 | 2 | 0.13 | 0.875 | porAIntermediate | 0.004 | 0.05 | 0.07 | 0.94 |
|  |  |  |  |  | porAHigh | 0.02 | 0.05 | 0.48 | 0.63 |
| fetA | 0.38 | 2 | 3.82 | **0.02** | fetAIntermediate | 0.02 | 0.13 | 0.18 | 0.85 |
|  |  |  |  |  | fetAHigh | 0.22 | 0.09 | 2.48 | **0.01** |
| nadA | 0.06 | 2 | 0.56 | 0.57 | nadAIntermediate | 0.04 | 0.06 | 0.57 | 0.57 |
|  |  |  |  |  | nadAHigh | -0.04 | 0.06 | -0.78 | 0.44 |
| hpuA | 0.08 | 1 | 1.54 | 0.216 | hpuAON | -0.05 | 0.04 | -1.24 | 0.22 |
| nalP | 0.01 | 1 | 0.18 | 0.673 | nalPON | -0.02 | 0.04 | -0.42 | 0.67 |
| mspA | 0.02 | 1 | 0.43 | 0.51 | mspAON | -0.03 | 0.05 | -0.66 | 0.51 |
| hmbR | 0.03 | 1 | 0.55 | 0.46 | hmbRON | -0.03 | 0.05 | -0.74 | 0.46 |
| pilC1 | 0.0009 | 1 | 0.018 | 0.89 | pilC1ON | 0.01 | 0.04 | 0.14 | 0.89 |
| pilC2 | 0.0002 | 1 | 0.004 | 0.95 | pilC2ON | 0.002 | 0.04 | 0.06 | 0.95 |
| modA | 0.01 | 1 | 0.11 | 0.74 | modAON | -0.02 | 0.05 | -0.34 | 0.74 |
| modB | 0.01 | 1 | 0.18 | 0.67 | modBON | -0.02 | 0.05 | -0.43 | 0.67 |

Table 1.6.7. Regression model: *RPMI_tmid ~ group + porA + fetA + nadA + hpuA + nalP + mspA + hmbR + pilC1 + pilC2 + modA + modB*

| ***Factors*** | ***Sum of squares*** | ***DF*** | ***F-statistic*** | ***p-value*** |
| --- | --- | --- | --- | --- |
| group | 28.90 | 2 | 0.48 | 0.62 |
| porA | 29.40 | 2 | 0.49 | 0.62 |
| fetA | 79.80 | 2 | 1.32 | 0.27 |
| nadA | 23.00 | 2 | 0.38 | 0.68 |
| hpuA | 1.30 | 1 | 0.04 | 0.83 |
| nalP | 15.40 | 1 | 0.51 | 0.48 |
| mspA | 2.00 | 1 | 0.07 | 0.80 |
| hmbR | 10.50 | 1 | 0.35 | 0.56 |
| pilC1 | 2.10 | 1 | 0.07 | 0.79 |
| pilC2 | 2.20 | 1 | 0.07 | 0.79 |
| modA | 31.50 | 1 | 1.04 | 0.31 |
| modB | 2.00 | 1 | 0.07 | 0.80 |

Table 1.6.8. Regression model: *LDH ~ group + porA + fetA + nadA + hpuA + nalP + mspA + hmbR + pilC1 + pilC2 + modA + modB*

| **Factors** | **Sum of squares** | **DF** | **F-statistic** | **p-value** | **Coefficients** | | | | |
| --- | --- | --- | --- | --- | --- | --- | --- | --- | --- |
|  |  |  |  |  |  | **Estimate** | **Std. Error** | **t value** | **P-value** |
|  |  |  |  |  | (Intercept) | 39.35 | 5.66 | 6.95 | **1.13e-10** |
| group | 7512.0 | 2 | 19.09 | **4.32e-08** | groupInvasive_Original | 19.33 | 3.85 | 5.03 | **1.45e-06** |
|  |  |  |  |  | groupInvasive_2013 | 20.46 | 3.47 | 5.89 | **2.56e-08** |
| porA | 1153.2 | 2 | 2.93 | 0.06 | porAIntermediate | -0.83 | 3.43 | -0.24 | 0.81 |
|  |  |  |  |  | porAHigh | -6.52 | 2.94 | -2.22 | **0.03** |
| fetA | 659.1 | 2 | 1.68 | 0.19 | fetAIntermediate | 9.87 | 8.03 | 1.23 | 0.22 |
|  |  |  |  |  | fetAHigh | -3.09 | 5.57 | -0.56 | 0.58 |
| nadA | 725.4 | 2 | 1.84 | 0.16 | nadAIntermediate | -5.85 | 3.89 | -1.51 | 0.13 |
|  |  |  |  |  | nadAHigh | 3.24 | 3.56 | 0.91 | 0.36 |
| hpuA | 230.2 | 1 | 1.17 | 0.28 | hpuAON | -2.53 | 2.34 | -1.08 | 0.28 |
| nalP | 178.3 | 1 | 0.91 | 0.34 | nalPON | -2.38 | 2.50 | -0.95 | 0.34 |
| mspA | 312.6 | 1 | 1.59 | 0.21 | mspAON | 4.07 | 3.23 | 1.26 | 0.21 |
| hmbR | 0.0 | 1 | 0.00 | 0.99 | hmbRON | 0.00 | 2.83 | 0.00 | 0.99 |
| pilC1 | 7.7 | 1 | 0.04 | 0.84 | pilC1ON | -0.55 | 2.78 | -0.20 | 0.84 |
| pilC2 | 29.8 | 1 | 0.15 | 0.70 | pilC2ON | 0.99 | 2.54 | 0.39 | 0.70 |
| modA | 134.7 | 1 | 0.68 | 0.41 | modAON | 2.38 | 2.88 | 0.83 | 0.41 |
| modB | 82.5 | 1 | 0.42 | 0.52 | modBON | 1.87 | 2.89 | 0.65 | 0.52 |

Table 1.6.9. Regression model: *Adhesion ~ group + porA + fetA + nadA + hpuA + nalP + mspA + hmbR + pilC1 + pilC2 + modA + modB*

| **Factors** | **Sum of squares** | **DF** | **F-statistic** | **p-value** | **Coefficients** | | | | |
| --- | --- | --- | --- | --- | --- | --- | --- | --- | --- |
|  |  |  |  |  |  | **Estimate** | **Std. Error** | **t value** | **P-value** |
|  |  |  |  |  | (Intercept) | 2.54e+08 | 5.86e+07 | 4.33 | **2.79e-0****5** |
| group | 2.40e+17 | 2 | 5.68 | **0.004** | groupInvasive_Original | -9.33e+07 | 3.98e+07 | -2.34 | **0.02** |
|  |  |  |  |  | groupInvasive_2013 | -1.20e+08 | 3.60e+07 | -3.34 | **0.001** |
| porA | 2.18e+16 | 2 | 0.52 | 0.60 | porAIntermediate | -3.60e+07 | 3.55e+07 | -1.01 | 0.31 |
|  |  |  |  |  | porAHigh | -1.32e+07 | 3.05e+07 | -0.44 | 0.66 |
| fetA | 5.60e+16 | 2 | 1.33 | 0.27 | fetAIntermediate | -1.15e+08 | 8.32e+07 | -1.38 | 0.17 |
|  |  |  |  |  | fetAHigh | -8.31e+07 | 5.76e+07 | -1.44 | 0.15 |
| nadA | 2.32e+16 | 2 | 0.55 | 0.58 | nadAIntermediate | -3.97e+07 | 4.02e+07 | -0.99 | 0.33 |
|  |  |  |  |  | nadAHigh | -1.93e+07 | 3.69e+07 | -0.52 | 0.60 |
| hpuA | 4.88e+16 | 1 | 2.32 | 0.13 | hpuAON | 3.68e+07 | 2.42e+07 | 1.52 | 0.13 |
| nalP | 2.99e+16 | 1 | 1.42 | 0.24 | nalPON | -3.08e+07 | 2.59e+07 | -1.19 | 0.24 |
| mspA | 2.79e+16 | 1 | 1.33 | 0.25 | mspAON | -3.85e+07 | 3.35e+07 | -1.15 | 0.25 |
| hmbR | 1.99e+14 | 1 | 0.01 | 0.92 | hmbRON | 2.85e+06 | 2.93e+07 | 0.10 | 0.92 |
| pilC1 | 4.75e+16 | 1 | 2.25 | 0.14 | pilC1ON | 4.32e+07 | 2.88e+07 | 1.50 | 0.14 |
| pilC2 | 3.76e+16 | 1 | 1.78 | 0.18 | pilC2ON | -3.51e+07 | 2.63e+07 | -1.34 | 0.18 |
| modA | 1.61e+14 | 1 | 0.01 | 0.93 | modAON | 2.61e+06 | 2.98e+07 | 0.09 | 0.93 |
| modB | 4.79e+16 | 1 | 2.27 | 0.13 | modBON | 4.50e+07 | 2.99e+07 | 1.51 | 0.13 |

Table 1.6.10. Regression model: *SBAD ~ group + porA + fetA + nadA + hpuA + nalP + mspA + hmbR + pilC1 + pilC2 + modA + modB*

| **Factors** | **Sum of squares** | **DF** | **F-statistic** | **p-value** | **Coefficients** | | | | |
| --- | --- | --- | --- | --- | --- | --- | --- | --- | --- |
|  |  |  |  |  |  | **Estimate** | **Std. Error** | **t value** | **P-value** |
|  |  |  |  |  | (Intercept) | 71.90 | 7.61 | 9.45 | <2e-16 |
| group | 4 | 2 | 0.01 | 0.99 | groupInvasive_Original | 0.56 | 5.17 | 0.11 | 0.91 |
|  |  |  |  |  | groupInvasive_2013 | 0.42 | 4.67 | 0.09 | 0.93 |
| porA | 90 | 2 | 0.13 | 0.88 | porAIntermediate | -0.27 | 4.61 | -0.06 | 0.95 |
|  |  |  |  |  | porAHigh | 1.61 | 3.96 | 0.41 | 0.68 |
| fetA | 1148 | 2 | 1.62 | 0.20 | fetAIntermediate | 5.43 | 10.79 | 0.50 | 0.62 |
|  |  |  |  |  | fetAHigh | -9.28 | 7.48 | -1.24 | 0.22 |
| nadA | 630 | 2 | 0.89 | 0.41 | nadAIntermediate | 3.89 | 5.22 | 0.75 | 0.46 |
|  |  |  |  |  | nadAHigh | -4.58 | 4.79 | -0.96 | 0.34 |
| hpuA | 1543 | 1 | 4.34 | **0.04** | hpuAON | 6.54 | 3.14 | 2.08 | 0.04 |
| nalP | 583 | 1 | 1.64 | 0.20 | nalPON | 4.31 | 3.36 | 1.28 | 0.20 |
| mspA | 147 | 1 | 0.41 | 0.52 | mspAON | -2.79 | 4.34 | -0.64 | 0.52 |
| hmbR | 0 | 1 | 0.00 | 1.00 | hmbRON | 0.02 | 3.80 | 0.01 | 0.99 |
| pilC1 | 7 | 1 | 0.02 | 0.89 | pilC1ON | -0.51 | 3.74 | -0.14 | 0.89 |
| pilC2 | 174 | 1 | 0.49 | 0.49 | pilC2ON | 2.38 | 3.41 | 0.70 | 0.49 |
| modA | 126 | 1 | 0.35 | 0.55 | modAON | -2.30 | 3.87 | -0.60 | 0.55 |
| modB | 229 | 1 | 0.65 | 0.42 | modBON | -3.12 | 3.88 | -0.80 | 0.42 |

Table 1.6.11. Regression model: *AD.HI ~ group + porA + fetA + nadA + hpuA + nalP + mspA + hmbR + pilC1 + pilC2 + modA + modB*

| **Factors** | **Sum of squares** | **DF** | **F-statistic** | **p-value** | **Coefficients** | | | | |
| --- | --- | --- | --- | --- | --- | --- | --- | --- | --- |
|  |  |  |  |  |  | **Estimate** | **Std. Error** | **t value** | **P-value** |
|  |  |  |  |  | (Intercept) | 0.44 | 0.35 | 1.27 | 0.21 |
| group | 12.49 | 2 | 8.43 | **0.0003** | groupInvasive_Original | 0.94 | 0.24 | 4.00 | **9.88e-05** |
|  |  |  |  |  | groupInvasive_2013 | 0.67 | 0.21 | 3.13 | **0.002** |
| porA | 1.65 | 2 | 1.12 | 0.33 | porAIntermediate | 0.10 | 0.21 | 0.48 | 0.63 |
|  |  |  |  |  | porAHigh | -0.18 | 0.18 | -0.99 | 0.32 |
| fetA | 0.16 | 2 | 0.11 | 0.90 | fetAIntermediate | -0.20 | 0.49 | -0.41 | 0.68 |
|  |  |  |  |  | fetAHigh | -0.14 | 0.34 | -0.40 | 0.69 |
| nadA | 1.39 | 2 | 0.94 | 0.39 | nadAIntermediate | -0.11 | 0.24 | -0.46 | 0.65 |
|  |  |  |  |  | nadAHigh | -0.30 | 0.22 | -1.35 | 0.18 |
| hpuA | 0.58 | 1 | 0.79 | 0.38 | hpuAON | 0.13 | 0.14 | 0.89 | 0.38 |
| nalP | 1.23 | 1 | 1.66 | 0.20 | nalPON | 0.20 | 0.15 | 1.29 | 0.20 |
| mspA | 0.02 | 1 | 0.03 | 0.87 | mspAON | 0.03 | 0.20 | 0.16 | 0.87 |
| hmbR | 2.95 | 1 | 3.98 | **0.048** | hmbRON | -0.35 | 0.17 | -1.99 | **0.048** |
| pilC1 | 0.12 | 1 | 0.16 | 0.69 | pilC1ON | 0.07 | 0.17 | 0.41 | 0.69 |
| pilC2 | 0.55 | 1 | 0.75 | 0.39 | pilC2ON | 0.13 | 0.16 | 0.86 | 0.39 |
| modA | 1.92 | 1 | 2.60 | 0.11 | modAON | 0.29 | 0.18 | 1.61 | 0.11 |
| modB | 0.00 | 1 | 0.00 | 0.99 | modBON | 0.00 | 0.18 | -0.01 | 0.99 |

**1.7 Phasotype groups and correlation testing.**

We combined the PV gene expressions into specific functional group. Three genes - *fetA*, *hpuA*, and *hmbR* - are involved in iron acquisition and were therefore combined to form the “IRON” phasotype. Two genes - *nadA* and *porA* - are encode adhesins or major porins and were therefore combined to form the “ADHESIN” phasotype. Two genes - *nalP* and *mspA* - are autotransporters and were therefore combined to form the “AUTO” phasotype. Two genes - *pilC1* and *pilC2* – are modifiers of Type IV pilus functions and were combined to form the “PILIN” phasotype. Two genes - *modA* and *modB* - encode DNA methyltransferases as part of restriction-modification systems and were combined to form the “MOD” phasotype.

We used the Cramer’s V to study the correlations between phasotype and group and between different phasotypes. Strong associations were observed between the group and PILIN (Cramer’s V = 0.32) and ADHESIN (Cramer’s V = 0.43) phasotypes. Moderate associations were observed between the group and IRON (Cramer’s V = 0.25) phasotype while Weak associations (Cramer’s V = 0) were observed between the group and the AUTO and MOD phasotypes. We observe strong associations between: IRON and AUTO (Cramer’s V = 0.37); PILIN and ADHESIN (Cramer’s V = 0.2) and moderate association between IRON and ADHESIN (Cramer’s V = 0.09); PILIN and IRON (Cramer’s V = 0.1); IRON and MOD (Cramer’s V = 0.12); ADHESIN and MOD (Cramer’s V = 0.13) phasotypes. Weak associations were observed between the rest of the phasotypes.

**1.8. Non-parametric method for detecting association between phasotypes and phenotypic variation.**

We used a Kruskal-Wallis test to test for mean differences in phenotypic data for the different expression states of the phasotypes. A Wilcoxon test was then used to perform pairwise comparisons of phenotype means between different combinations of gene expression in the phasotypes. Results for these tests are shown in the supplementary figures file: Figure A21 – A25 and Table 1.8.1. Note, Wilcoxon test was only performed for phenotypes that had significant differences (bold) in the Kruskal-Wallis test. The PILIN phasotype was associated with the following phenotypes, BHI_r, BHI_tmid, RPMI_k, LDH, and AD.HI. The AUTO phasotype was associated with only Biofilms while the ADHESIN phasotype was associated with BHI-tmid and RPMI_r, LDH and AD.HI. The IRON phasotype was associated with RPMI_k while MOD phasotype was associated with BHI_tmid.

Table 1.8.1. Association between phasotypes and phenotypes.

| **Phenotype** | **Kruskal-Wallis rank sum test** (**p-value)** | | | | |
| --- | --- | --- | --- | --- | --- |
|  | **PILIN** | **AUTO** | **IRON** | **ADHESIN** | **MOD** |
| Biofilm | 0.99 | **0.007** | 0.25 | 0.17 | 0.11 |
|  |  |  |  |  |  |
|  |  |  |  |  |  |
| BHI_k | 0.65 | 0.41 | 0.75 | 0.73 | 0.97 |
| BHI_r | **0.002** | 0.28 | 0.08 | 0.47 | 0.11 |
|  |  |  |  |  |  |
|  |  |  |  |  |  |
|  |  |  |  |  |  |
| BHI_tmid | **0.03** | 0.12 | 0.06 | **0.04** | 0.007 |
|  |  |  |  |  |  |
|  |  |  |  |  |  |
| RPMI_k | **0.046** | 0.22 | **0.03** | 0.05 | 0.63 |
|  |  |  |  |  |  |
|  |  |  |  |  |  |
| RPMI_r | 0.14 | 0.89 | 0.79 | **0.02** | 0.96 |
|  |  |  |  |  |  |
|  |  |  |  |  |  |
| RPMI_tmid | 0.23 | 0.27 | 0.45 | 0.19 | 0.53 |
| LDH | **0.002** | 0.65 | 0.26 | **0.001** | 0.28 |
|  |  |  |  |  |  |
|  |  |  |  |  |  |
|  |  |  |  |  |  |
| Adhesion | 0.14 | 0.46 | 0.18 | 0.7 | 0.68 |
| SBAD | 0.94 | 0.7 | 0.35 | 0.83 | 0.06 |
| AD.HI | **0.008** | 0.75 | 0.06 | **0.0005** | 0.59 |
|  |  |  |  |  |  |
|  |  |  |  |  |  |

**1.9 Type II/III ANOVA Type II/III ANOVA tests for detecting association of group and individual phasotype groups with phenotypic variance**

We used type II/III ANOVA to analyse whether the phasotypes contributed to the amount of variance observed in phenotype while holding group constant. We also checked for interactions between the two factors(phasotype:group). These results are presented in table 2 in the main manuscript, but for more details please see below. The variance in phenotypes was mostly explained by group in most of the phenotypes. Interaction between AUTO and group explained the variance in Biofilms, BHI_tmid and RPMI_k while both group and AUTO explained the variance in RPMI_k when holding the other factor constant respectively. Both IRON and group explained the variance in Biofilms, RPMI_k, RPMI_r and LDH when holding the other factor constant resp. Both MOD and group explained the variance in BHI_tmid when holding the other factor constant respectively. P-values in bold shows the factors (either group, phasotype, or interaction term) that were significant in explaining the variance in the phenotypes.

Table 1.9.1. Analysis of variance in Adhesion contributed by group and/or phasotypes.

| **Model** | **Factors** | **Sum of squares** | **Degree of Freedom (DF)** | **F-statistic** | **p-value** |
| --- | --- | --- | --- | --- | --- |
| Adhesion ~ PILIN + group + PILIN:group | PILIN | 1.13e+17 | 3 | 1.80 | 0.15 |
|  | group | 3.84e+17 | 2 | 9.19 | **1.71e-04** |
|  | PILIN:group | 9.56e+16 | 6 | 0.76 | 0.60 |
| Adhesion ~ AUTO + group + AUTO:group | AUTO | 3.11e+16 | 3 | 0.49 | 0.69 |
|  | group | 5.37e+17 | 2 | 12.77 | **7.50e-06** |
|  | AUTO:group | 1.37e+17 | 5 | 1.30 | 0.27 |
| Adhesion ~ IRON + group + IRON:group | IRON | 1.48e+17 | 9 | 0.76 | 0.66 |
|  | group | 4.05e+17 | 2 | 9.34 | **1.54e-04** |
|  | IRON:group | 9.22e+16 | 7 | 0.61 | 0.75 |
| Adhesion ~ ADHESIN + group + ADHESIN:group | ADHESIN | 1.39e+17 | 8 | 0.83 | 0.57 |
|  | group | 5.11e+17 | 2 | 12.30 | **1.20e-05** |
|  | ADHESIN:group | 3.40e+17 | 13 | 1.26 | 0.24 |
| Adhesion ~ MOD + group + MOD:group | MOD | 6.09e+16 | 3 | 0.97 | 0.41 |
|  | group | 5.74e+17 | 2 | 13.72 | **3.35e-06** |
|  | MOD:group | 1.42e+17 | 6 | 1.13 | 0.35 |

Table 1.9.2. Analysis of variance in Biofilms contributed by group and/or phasotypes.

| **Model** | **Factors** | **Sum of squares** | **Degree of Freedom (DF)** | **F-statistic** | **p-value** |
| --- | --- | --- | --- | --- | --- |
| Biofilms ~ PILIN + group + PILIN:group | PILIN | 0.23 | 3 | 1.21 | 0.31 |
|  | group | 1.13 | 2 | 8.97 | **2.09e-04** |
|  | PILIN:group | 0.33 | 6 | 0.87 | 0.52 |
| Biofilms ~ AUTO + group + AUTO:group | AUTO | 0.69 | 2 | 6.87 | **1.39e-03** |
|  | group | 0.57 | 1 | 11.28 | **9.90e-04** |
|  | AUTO:group | 0.84 | 5 | 3.33 | **0.01** |
| Biofilms ~ IRON + group + IRON:group | IRON | 1.40 | 9 | 2.76 | **0.01** |
|  | group | 0.42 | 2 | 3.76 | **0.03** |
|  | IRON:group | 0.57 | 7 | 1.44 | 0.20 |
| Biofilms ~ ADHESIN + group + ADHESIN:group | ADHESIN | 0.67 | 8 | 1.38 | 0.21 |
|  | group | 0.63 | 2 | 5.19 | **0.01** |
|  | ADHESIN:group | 0.93 | 13 | 1.18 | 0.30 |
| Biofilms ~ MOD + group + MOD:group | MOD | 0.08 | 3 | 0.43 | 0.73 |
|  | group | 0.91 | 2 | 7.00 | **1.24e-03** |
|  | MOD:group | 0.20 | 6 | 0.51 | 0.80 |

Table 1.9.3. Analysis of variance in BHI_k contributed by group and/or phasotypes.

| **Model** | **Factors** | **Sum of squares** | **Degree of Freedom (DF)** | **F-statistic** | **p-value** |
| --- | --- | --- | --- | --- | --- |
| BHI_k ~ PILIN + group + PILIN:group | PILIN | 0.02 | 3 | 0.49 | 0.69 |
|  | group | 0.01 | 2 | 0.45 | 0.64 |
|  | PILIN:group | 0.02 | 6 | 0.23 | 0.97 |
| BHI_k ~ AUTO + group + AUTO:group | AUTO | 0.05 | 3 | 1.23 | 0.30 |
|  | group | 0.03 | 2 | 1.14 | 0.32 |
|  | AUTO:group | 0.05 | 5 | 0.81 | 0.55 |
| BHI_k ~ IRON + group + IRON:group | IRON | 0.08 | 9 | 0.67 | 0.73 |
|  | group | 0.04 | 2 | 1.62 | 0.20 |
|  | IRON:group | 0.10 | 7 | 1.11 | 0.36 |
| BHI_k ~ ADHESIN + group + ADHESIN:group | ADHESIN | 0.06 | 8 | 0.59 | 0.79 |
|  | group | 0.04 | 2 | 1.68 | 0.19 |
|  | ADHESIN:group | 0.23 | 13 | 1.37 | 0.18 |
| BHI_k ~ MOD + group + MOD:group | MOD | 0.00 | 3 | 0.08 | 0.97 |
|  | group | 0.03 | 2 | 1.16 | 0.31 |
|  | MOD:group | 0.06 | 6 | 0.75 | 0.61 |

Table 1.9.4. Analysis of variance in BHI_r contributed by group and/or phasotypes.

| **Model** | **Factors** | **Sum of squares** | **Degree of Freedom (DF)** | **F-statistic** | **p-value** |
| --- | --- | --- | --- | --- | --- |
| BHI_r ~ PILIN + group + PILIN:group | PILIN | 0.02 | 3 | 0.60 | 0.61 |
|  | group | 0.04 | 2 | 2.24 | 0.11 |
|  | PILIN:group | 0.02 | 6 | 0.39 | 0.89 |
| BHI_r ~ AUTO + group + AUTO:group | AUTO | 0.01 | 3 | 0.45 | 0.72 |
|  | group | 0.08 | 2 | 4.35 | **0.01** |
|  | AUTO:group | 0.02 | 5 | 0.52 | 0.76 |
| BHI_r ~ IRON + group + IRON:group | IRON | 0.04 | 9 | 0.47 | 0.90 |
|  | group | 0.06 | 2 | 3.24 | **0.04** |
|  | IRON:group | 0.03 | 7 | 0.46 | 0.87 |
| BHI_r ~ ADHESIN + group + ADHESIN:group | ADHESIN | 0.04 | 8 | 0.52 | 0.84 |
|  | group | 0.10 | 2 | 5.56 | **0.005** |
|  | ADHESIN:group | 0.09 | 13 | 0.79 | 0.67 |
| BHI_r ~ MOD + group + MOD:group | MOD | 0.00 | 3 | 0.18 | 0.91 |
|  | group | 0.09 | 2 | 4.67 | **0.01** |
|  | MOD:group | 0.03 | 6 | 0.47 | 0.83 |

Table 1.9.5. Analysis of variance in BHI_tmid contributed by group and/or phasotypes.

| **Model** | **Factors** | **Sum of squares** | **Degree of Freedom (DF)** | **F-statistic** | **p-value** |
| --- | --- | --- | --- | --- | --- |
| BHI_tmid ~ PILIN + group + PILIN:group | PILIN | 16.39 | 3 | 1.11 | 0.35 |
|  | group | 103.57 | 2 | 10.49 | **5.41e-05** |
|  | PILIN:group | 48.90 | 6 | 1.65 | 0.14 |
| BHI_tmid ~ AUTO + group + AUTO:group | AUTO | 26.41 | 2 | 2.75 | 0.07 |
|  | group | 9.32 | 1 | 1.94 | 0.17 |
|  | AUTO:group | 58.54 | 5 | 2.44 | **0.04** |
| BHI_tmid ~ IRON + group + IRON:group | IRON | 59.40 | 9 | 1.34 | 0.22 |
|  | group | 183.98 | 2 | 18.71 | **5.98e-08** |
|  | IRON:group | 43.16 | 7 | 1.25 | 0.28 |
| BHI_tmid ~ ADHESIN + group + ADHESIN:group | ADHESIN | 43.28 | 8 | 1.06 | 0.40 |
|  | group | 128.09 | 2 | 12.50 | **1.02e-05** |
|  | ADHESIN:group | 55.04 | 13 | 0.83 | 0.63 |
| BHI_tmid ~ MOD + group + MOD:group | MOD | 49.13 | 3 | 3.42 | **0.02** |
|  | group | 142.21 | 2 | 14.87 | **1.27e-06** |
|  | MOD:group | 39.33 | 6 | 1.37 | 0.23 |

Table 1.9.6. Analysis of variance in RPMI_k contributed by group and/or phasotypes.

| **Model** | **Factors** | **Sum of squares** | **Degree of Freedom (DF)** | **F-statistic** | **p-value** |
| --- | --- | --- | --- | --- | --- |
| RPMI_k ~ PILIN + group + PILIN:group | PILIN | 0.01 | 3 | 0.27 | 0.85 |
|  | group | 0.08 | 2 | 2.35 | 0.10 |
|  | PILIN:group | 0.04 | 6 | 0.41 | 0.87 |
| RPMI_k ~ AUTO + group + AUTO:group | AUTO | 0.18 | 3 | 4.19 | **0.01** |
|  | group | 0.15 | 2 | 5.00 | **0.01** |
|  | AUTO:group | 0.18 | 5 | 2.40 | **0.04** |
| RPMI_k ~ IRON + group + IRON:group | IRON | 0.46 | 9 | 3.47 | **6.56e-04** |
|  | group | 0.22 | 2 | 7.51 | **7.92e-04** |
|  | IRON:group | 0.02 | 7 | 0.17 | 0.99 |
| RPMI_k ~ ADHESIN + group + ADHESIN:group | ADHESIN | 0.10 | 8 | 0.74 | 0.65 |
|  | group | 0.07 | 2 | 2.08 | 0.13 |
|  | ADHESIN:group | 0.25 | 13 | 1.19 | 0.29 |
| RPMI_k ~ MOD + group + MOD:group | MOD | 0.02 | 3 | 0.47 | 0.71 |
|  | group | 0.11 | 2 | 3.28 | **0.04** |
|  | MOD:group | 0.04 | 6 | 0.40 | 0.88 |

Table 1.9.7. Analysis of variance in RPMI_r contributed by group and/or phasotypes.

| **Model** | **Factors** | **Sum of squares** | **Degree of Freedom (DF)** | **F-statistic** | **p-value** |
| --- | --- | --- | --- | --- | --- |
| RPMI_r ~ PILIN + group + PILIN:group | PILIN | 0.10 | 3 | 0.69 | 0.56 |
|  | group | 1.06 | 2 | 10.54 | **5.20e-05** |
|  | PILIN:group | 0.37 | 6 | 1.22 | 0.30 |
| RPMI_r ~ AUTO + group + AUTO:group | AUTO | 0.20 | 3 | 1.34 | 0.26 |
|  | group | 1.60 | 2 | 16.03 | **4.81e-07** |
|  | AUTO:group | 0.30 | 5 | 1.20 | 0.31 |
| RPMI_r ~ IRON + group + IRON:group | IRON | 0.85 | 9 | 2.00 | **0.04** |
|  | group | 2.10 | 2 | 22.16 | **4.06e-09** |
|  | IRON:group | 0.39 | 7 | 1.18 | 0.32 |
| RPMI_r ~ ADHESIN + group + ADHESIN:group | ADHESIN | 0.35 | 8 | 0.84 | 0.57 |
|  | group | 1.00 | 2 | 9.49 | **1.37e-04** |
|  | ADHESIN:group | 0.42 | 13 | 0.62 | 0.84 |
| RPMI_r ~ MOD + group + MOD:group | MOD | 0.05 | 3 | 0.33 | 0.80 |
|  | group | 1.48 | 2 | 14.44 | **1.82e-06** |
|  | MOD:group | 0.28 | 6 | 0.92 | 0.48 |

Table 1.9.8. Analysis of variance in RPMI_tmid contributed by group and/or phasotypes.

| **Model** | **Factors** | **Sum of squares** | **Degree of Freedom (DF)** | **F-statistic** | **p-value** |
| --- | --- | --- | --- | --- | --- |
| RPMI_tmid ~ PILIN + group + PILIN:group | PILIN | 8.00 | 3 | 0.09 | 0.96 |
|  | group | 24.90 | 2 | 0.43 | 0.65 |
|  | PILIN:group | 175.70 | 6 | 1.00 | 0.43 |
| RPMI_tmid ~ AUTO + group + AUTO:group | AUTO | 113.60 | 3 | 1.32 | 0.27 |
|  | group | 34.70 | 2 | 0.61 | 0.55 |
|  | AUTO:group | 136.60 | 5 | 0.95 | 0.45 |
| RPMI_tmid ~ IRON + group + IRON:group | IRON | 137.60 | 9 | 0.50 | 0.87 |
|  | group | 26.50 | 2 | 0.44 | 0.65 |
|  | IRON:group | 79.50 | 7 | 0.37 | 0.92 |
| RPMI_tmid ~ ADHESIN + group + ADHESIN:group | ADHESIN | 136.00 | 8 | 0.55 | 0.81 |
|  | group | 34.20 | 2 | 0.56 | 0.57 |
|  | ADHESIN:group | 188.90 | 13 | 0.47 | 0.94 |
| RPMI_tmid ~ MOD + group + MOD:group | MOD | 73.70 | 3 | 0.84 | 0.47 |
|  | group | 22.00 | 2 | 0.38 | 0.69 |
|  | MOD:group | 127.30 | 6 | 0.73 | 0.63 |

Table 1.9.9. Analysis of variance in LDH contributed by group and/or phasotypes.

| **Model** | **Factors** | **Sum of squares** | **Degree of Freedom (DF)** | **F-statistic** | **p-value** |
| --- | --- | --- | --- | --- | --- |
| LDH ~ PILIN + group + PILIN:group | PILIN | 324 | 3 | 0.50 | 0.68 |
|  | group | 11349 | 2 | 26.49 | **1.38e-10** |
|  | PILIN:group | 357 | 6 | 0.28 | 0.95 |
| LDH ~ AUTO + group + AUTO:group | AUTO | 1061.2 | 3 | 1.76 | 0.16 |
|  | group | 16372.9 | 2 | 40.73 | **6.84e-15** |
|  | AUTO:group | 1423.7 | 5 | 1.42 | 0.22 |
| LDH ~ IRON + group + IRON:group | IRON | 3758.9 | 9 | 2.16 | **0.03** |
|  | group | 15870.9 | 2 | 40.96 | **8.26e-15** |
|  | IRON:group | 1377.2 | 7 | 1.02 | 0.42 |
| LDH ~ ADHESIN + group + ADHESIN:group | ADHESIN | 2634.3 | 8 | 1.64 | 0.12 |
|  | group | 10237.1 | 2 | 25.47 | **3.75e-10** |
|  | ADHESIN:group | 2469.5 | 13 | 0.95 | 0.51 |
| LDH ~ MOD + group + MOD:group | MOD | 390 | 3 | 0.61 | 0.61 |
|  | group | 15211 | 2 | 35.80 | **1.89e-13** |
|  | MOD:group | 559 | 6 | 0.44 | 0.85 |

Table 1.9.10. Analysis of variance in SBAD contributed by group and/or phasotypes.

| **Model** | **Factors** | **Sum of squares** | **Degree of Freedom (DF)** | **F-statistic** | **p-value** |
| --- | --- | --- | --- | --- | --- |
| SBAD ~ PILIN + group + PILIN:group | PILIN | 76 | 3 | 0.07 | 0.98 |
|  | group | 377 | 2 | 0.53 | 0.59 |
|  | PILIN:group | 1986 | 6 | 0.92 | 0.48 |
| SBAD ~ AUTO + group + AUTO:group | AUTO | 619 | 3 | 0.58 | 0.63 |
|  | group | 561 | 2 | 0.79 | 0.45 |
|  | AUTO:group | 1829 | 5 | 1.04 | 0.40 |
| SBAD ~ IRON + group + IRON:group | IRON | 2982 | 9 | 0.95 | 0.49 |
|  | group | 75 | 2 | 0.11 | 0.90 |
|  | IRON:group | 2824 | 7 | 1.15 | 0.33 |
| SBAD ~ ADHESIN + group + ADHESIN:group | ADHESIN | 752 | 8 | 0.26 | 0.98 |
|  | group | 357 | 2 | 0.50 | 0.61 |
|  | ADHESIN:group | 5436 | 13 | 1.16 | 0.31 |
| SBAD ~ MOD + group + MOD:group | MOD | 2493 | 3 | 2.37 | 0.07 |
|  | group | 408 | 2 | 0.58 | 0.56 |
|  | MOD:group | 754 | 6 | 0.36 | 0.90 |

Table 1.9.11. Analysis of variance in AD.HI contributed by group and/or phasotypes.

| **Model** | **Factors** | **Sum of squares** | **Degree of Freedom (DF)** | **F-statistic** | **p-value** |
| --- | --- | --- | --- | --- | --- |
| AD.HI ~ PILIN + group + PILIN:group | PILIN | 0.51 | 3 | 0.22 | 0.88 |
|  | group | 26.87 | 2 | 17.46 | **1.50e-07** |
|  | PILIN:group | 2.15 | 6 | 0.47 | 0.83 |
| AD.HI ~ AUTO + group + AUTO:group | AUTO | 2.33 | 3 | 1.03 | 0.38 |
|  | group | 32.06 | 2 | 21.23 | **7.37e-09** |
|  | AUTO:group | 1.77 | 5 | 0.47 | 0.80 |
| AD.HI ~ IRON + group + IRON:group | IRON | 10.78 | 9 | 1.64 | 0.11 |
|  | group | 22.50 | 2 | 15.45 | **8.35e-07** |
|  | IRON:group | 3.17 | 7 | 0.62 | 0.74 |
| AD.HI ~ ADHESIN + group + ADHESIN:group | ADHESIN | 3.90 | 8 | 0.64 | 0.74 |
|  | group | 18.64 | 2 | 12.32 | **1.19e-05** |
|  | ADHESIN:group | 9.74 | 13 | 0.99 | 0.46 |
| AD.HI ~ MOD + group + MOD:group | MOD | 3.84 | 3 | 1.74 | 0.16 |
|  | group | 32.03 | 2 | 21.83 | **4.70e-09** |
|  | MOD:group | 4.23 | 6 | 0.96 | 0.45 |

**1.10 Type II/III ANOVA Type II/III ANOVA tests for detecting association of group and all phasotype groups with phenotypic variance**

We created models with all the phasotypes and group to see the factors that are associated with the phenotypes (Results also in Table 2 in the manuscript). Biofilm was associated with the AUTO phasotype while BHI_tmid, RPMI_r, LDH, Adhesion and AD.HI were associated with group. RPMI_k was associated with both IRON and group. P-value significance for all tables below is 0.05.

Table 1.10.1. Regression model: *Biofilms ~ PILIN + AUTO + IRON + ADHESIN + MOD + group*

| Factors | Sum of squares | DF | F-statistic | p-value |
| --- | --- | --- | --- | --- |
| PILIN | 0.14 | 3 | 0.90 | 0.44 |
| AUTO | 0.65 | 3 | 4.14 | **0.01** |
| IRON | 0.71 | 9 | 1.49 | 0.16 |
| ADHESIN | 0.53 | 8 | 1.27 | 0.27 |
| MOD | 0.03 | 3 | 0.20 | 0.90 |
| group | 0.22 | 2 | 2.06 | 0.13 |

Table 1.10.2. Regression model: *BHI_k ~ PILIN + AUTO + IRON + ADHESIN + MOD + group*

| Factors | Sum of squares | DF | F-statistic | p-value |
| --- | --- | --- | --- | --- |
| PILIN | 0.02 | 3 | 0.38 | 0.77 |
| AUTO | 0.05 | 3 | 1.13 | 0.34 |
| IRON | 0.07 | 9 | 0.54 | 0.85 |
| ADHESIN | 0.05 | 8 | 0.43 | 0.90 |
| MOD | 0.02 | 3 | 0.49 | 0.69 |
| group | 0.02 | 2 | 0.63 | 0.54 |

Table 1.10.3. Regression model: *BHI_r ~ PILIN + AUTO + IRON + ADHESIN + MOD + group*

| Factors | Sum of squares | DF | F-statistic | p-value |
| --- | --- | --- | --- | --- |
| PILIN | 0.02 | 3 | 0.68 | 0.56 |
| AUTO | 0.02 | 3 | 0.81 | 0.49 |
| IRON | 0.03 | 9 | 0.34 | 0.96 |
| ADHESIN | 0.07 | 8 | 0.85 | 0.56 |
| MOD | 0.01 | 3 | 0.24 | 0.87 |
| group | 0.03 | 2 | 1.32 | 0.27 |

Table 1.10.4. Regression model: *BHI_tmid ~ PILIN + AUTO + IRON + ADHESIN + MOD + group*

| Factors | Sum of squares | DF | F-statistic | p-value |
| --- | --- | --- | --- | --- |
| PILIN | 10.75 | 3 | 0.74 | 0.53 |
| AUTO | 24.85 | 3 | 1.72 | 0.17 |
| IRON | 57.21 | 9 | 1.32 | 0.23 |
| ADHESIN | 24.75 | 8 | 0.64 | 0.74 |
| MOD | 22.83 | 3 | 1.58 | 0.20 |
| group | 98.73 | 2 | 10.22 | 7.39e-05 |

Table 1.10.5. Regression model: *RPMI_k ~ PILIN + AUTO + IRON + ADHESIN + MOD + group*

| Factors | Sum of squares | DF | F-statistic | p-value |
| --- | --- | --- | --- | --- |
| PILIN | 0.01 | 3 | 0.30 | 0.83 |
| AUTO | 0.04 | 3 | 0.91 | 0.44 |
| IRON | 0.31 | 9 | 2.42 | **0.01** |
| ADHESIN | 0.11 | 8 | 0.93 | 0.49 |
| MOD | 0.03 | 3 | 0.69 | 0.56 |
| group | 0.20 | 2 | 7.06 | **1.22e-03** |

Table 1.10.6. Regression model: *RPMI_r ~ PILIN + AUTO + IRON + ADHESIN + MOD + group*

| Factors | Sum of squares | DF | F-statistic | p-value |
| --- | --- | --- | --- | --- |
| PILIN | 0.03 | 3 | 0.21 | 0.89 |
| AUTO | 0.05 | 3 | 0.32 | 0.81 |
| IRON | 0.67 | 9 | 1.49 | 0.16 |
| ADHESIN | 0.35 | 8 | 0.88 | 0.54 |
| MOD | 0.03 | 3 | 0.22 | 0.88 |
| group | 0.97 | 2 | 9.64 | **1.23e-04** |

Table 1.10.7. Regression model: *RPMI_tmid ~ PILIN + AUTO + IRON + ADHESIN + MOD + group*

| Factors | Sum of squares | DF | F-statistic | p-value |
| --- | --- | --- | --- | --- |
| PILIN | 6.8 | 3 | 0.07 | 0.97 |
| AUTO | 80 | 3 | 0.86 | 0.46 |
| IRON | 126 | 9 | 0.45 | 0.90 |
| ADHESIN | 166.7 | 8 | 0.67 | 0.71 |
| MOD | 53.5 | 3 | 0.58 | 0.63 |
| group | 70.6 | 2 | 1.14 | 0.32 |

Table 1.10.8. Regression model: *LDH ~ PILIN + AUTO + IRON + ADHESIN + MOD + group*

| Factors | Sum of squares | DF | F-statistic | p-value |
| --- | --- | --- | --- | --- |
| PILIN | 32 | 3 | 0.05 | 0.98 |
| AUTO | 664.1 | 3 | 1.12 | 0.35 |
| IRON | 1905.9 | 9 | 1.07 | 0.39 |
| ADHESIN | 2029.6 | 8 | 1.28 | 0.26 |
| MOD | 164.8 | 3 | 0.28 | 0.84 |
| group | 7525.8 | 2 | 18.95 | **5.64e-08** |

Table 1.10.9. Regression model: *Adhesion ~ PILIN + AUTO + IRON + ADHESIN + MOD + group*

| Factors | Sum of squares | DF | F-statistic | p-value |
| --- | --- | --- | --- | --- |
| PILIN | 1.18e+17 | 3 | 1.81 | 0.15 |
| AUTO | 4.99e+16 | 3 | 0.77 | 0.51 |
| IRON | 1.03e+17 | 9 | 0.53 | 0.85 |
| ADHESIN | 8.51e+16 | 8 | 0.49 | 0.86 |
| MOD | 5.87e+16 | 3 | 0.90 | 0.44 |
| group | 2.70e+17 | 2 | 6.23 | **2.60e-03** |

Table 1.10.10. Regression model: *SBAD ~ PILIN + AUTO + IRON + ADHESIN + MOD + group*

| Factors | Sum of squares | DF | F-statistic | p-value |
| --- | --- | --- | --- | --- |
| PILIN | 265 | 3 | 0.24 | 0.87 |
| AUTO | 834 | 3 | 0.75 | 0.52 |
| IRON | 2739 | 9 | 0.82 | 0.60 |
| ADHESIN | 738 | 8 | 0.25 | 0.98 |
| MOD | 1399 | 3 | 1.26 | 0.29 |
| group | 6 | 2 | 0.01 | 0.99 |

Table 1.10.11. Regression model: *AD.HI ~ PILIN + AUTO + IRON + ADHESIN + MOD + group*

| Factors | Sum of squares | DF | F-statistic | p-value |
| --- | --- | --- | --- | --- |
| PILIN | 0.93 | 3 | 0.41 | 0.74 |
| AUTO | 0.54 | 3 | 0.24 | 0.87 |
| IRON | 8.28 | 9 | 1.23 | 0.28 |
| ADHESIN | 2.74 | 8 | 0.46 | 0.88 |
| MOD | 3.79 | 3 | 1.70 | 0.17 |
| group | 10.57 | 2 | 7.09 | **0.001** |

**1.11 Predictive Models**

Tree based learning algorithms are some of the best supervised learning methods capable of capturing interaction and complex relationships in the data. Trees are non-parametric models since they have no underlying assumptions. Based on the type of target variable, there are two types of decision tree: classification trees, where the target variable is categorical and the tree is used to identify the class within which a target variable would likely fall into; regression trees, where the target variable is continuous and tree is used to predict it’s value. During the process of analysis, multiple trees may be created using several techniques called ensemble methods.

Ensemble learning is a method that combines results produced by different learners into one format, with the aim of producing better classification results and regression results. There are three most common methods. The first is Bagging (Bootstrap Aggregating) where successive bootstrap (resampled) samples of the data are selected and a prediction is derived from each of these samples. The final prediction is determined by either averaging each of the predictions (for a continuous outcome), or taking a majority vote (for classification). The second method is boosting, this is a sequenstial ensemble technique where model is improved using the information from previously grown weaker models. The process is continued for multiple iterations until a final model is built which will predict a more accurate outcome (eg., Adaboost, Gradient Descent, Xgboost). The third method is random forest (RF) which creates a large number of decision trees. Every observation is fed into every decision tree. The most common outcome for each observation is used as the final output. RF is less prone to overfitting therefore well adapted for both prediction and variable importance. It has an effective method for estimating missing data and maintains accuracy when a large proportion of the data are missing.

**1.11.1 FEATURE SELECTION:** **Recursive Feature Elimination**

Recursive Feature Elimination (RFE) is a wrapper-type feature selection algorithm for selecting the most relevant features in predicting the target variable in a predictive model. A machine learning algorithm is used in the core of the method (wrapped by RFE) to help select features. RFE applies a backward selection process to find the optimal combination of features. It first builds a model based on all features and calculates the importance of each feature in the model. It then rank-orders the features and removes the one(s) with the least importance iteratively based on model evaluation metrics (e.g., RMSE, accuracy, and Kappa). Feature importance can be computed based on the model (e.g., the random forest importance criterion) or using a model-independent metric (e.g., ROC curve analysis). This process continues until a smaller subset of features is retained in the model (technically, RFE can still keep all (or most) of the features in the final model). Variable importance is calculated as follows: First compute the model Mean Squared Error(MSE)/ accuracy. Then for each variable in the model, permute the variable, and calculate the new model’s MSE/accuracy according to the variable permutation. Take the difference between model MSE/accuracy and new model MSE/accuracy then rank variables’ importance according to the value of the %incMSE/accuracy. Usually a positive number is expected, the greater the value the better. A negative number denotes that the random permutation worked better, which shows that the variable is probably not predictive enough (i.e., not important). Metrics used for classification are accuracy and Kappa. Classification accuracy is the proportion of outcome that were correctly predicted. It is used when there are nearly the same number of samples in each class. Cohen’s Kappa accounts for class imbalances and can be generalized to problems with more than two groups. Kappa of 1 shows complete concordance/perfect accuracy while -1 shows complete discordance, near 0 shows no relationship between model prediction and true results.

For all the predictive analyses, we first selected the control options to random forest since it has a built-in mechanism for computing feature importance. We also used repeated 10-fold cross-validation with 5 repeats to improve the performance of feature selection with RFE. We set random seed so the outcome would be reproducible and randomly split the dataset into training (70%) and test (30%) datasets.

**1.11.2 Group analyses with all phenotypes and PV genes/phasotypes**

We used sizes = c(1:27) in the “rfe” function so that the function tries all possible solutions (i.e., only 1 feature, 2 features, …, 27 features) to find the optimal features. The output indicates that RFE recommends 14 features for the best model where both accuracy (70%) and Kappa (0.54) reach the maximum level. The 14 features selected by RFE are: AD.HI, LDH, porA, ADHESIN, RPMI_r, fetA, Adhesion, PILIN, pilC1, BHI_r, BHI_tmid, RPMI_tmid, RPMI_k, pilC2. Checking the model performance using the test dataset gave an accuracy (79%) and Kappa (0.69) values that are slightly higher to those obtained from the training dataset (Figure A27 in the Supplementary figures datafile)

**1.11.3 Group analyses with phenotype variables only**

We used sizes = c(1:11) in the “rfe” function so that the function tries all possible solutions to find the optimal features. The output indicates that RFE recommends 8 features for the best model where both accuracy (60%) and Kappa (0.4) reach the maximum level. The features selected by RFE are: AD.HI, LDH, RPMI_r, Adhesion, BHI_r, BHI_tmid, RPMI_tmid, and RPMI_k. Using the test dataset to check the model performance gave similar accuracy (60%) and Kappa (0.41).


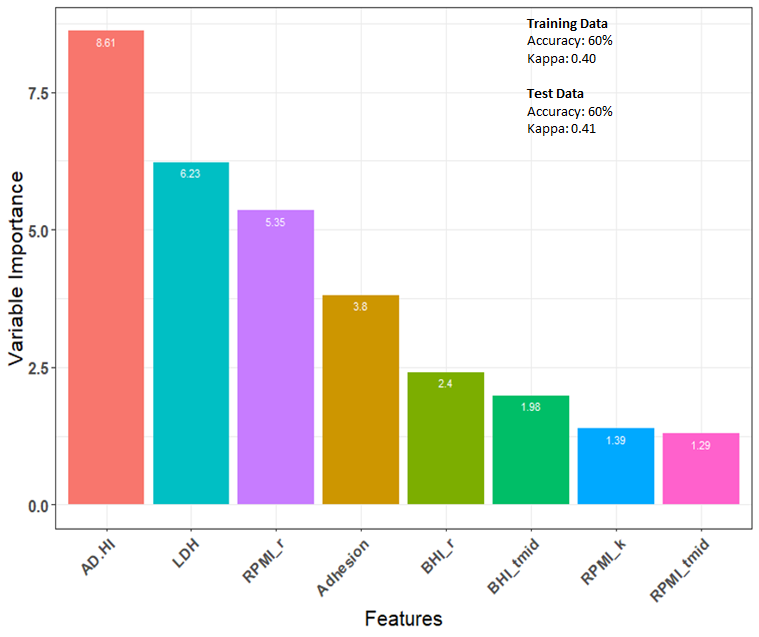
 Figure 1.11.1. Phenotypes identified in predicting group

**1.11.4. Group analyses with PV genes and phasotypes only**

We used sizes = c(1:16) in the “rfe” function so that the function tries all possible solutions to find the optimal features. The output indicates that RFE recommends 7 features for the best model where both accuracy (81%) and Kappa (0.55) reach the maximum level. The features selected by RFE are: fetA, PILIN, porA, ADHESIN, IRON, pilC1 and pilC2. Using the test dataset to check the model performance gave slightly higher accuracy (85%) and Kappa (0.67).


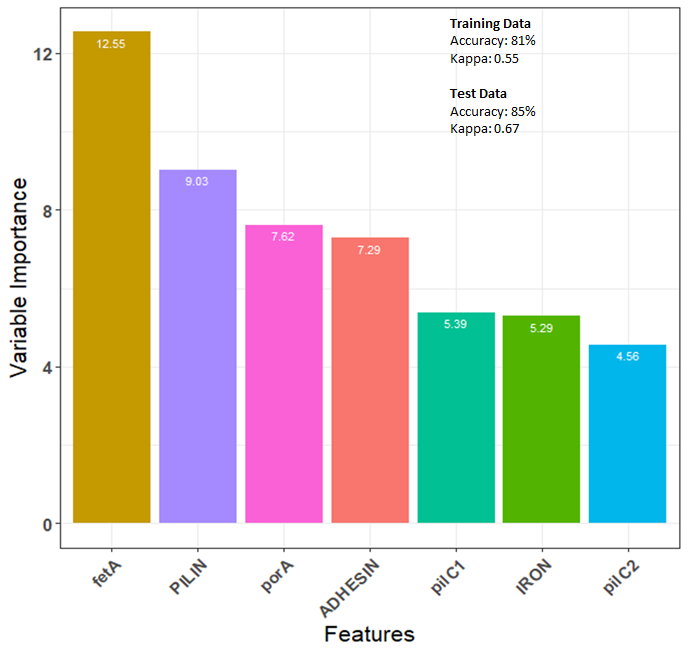
 Figure 1.11.2. Phasotypes and PV genes identified in predicting group

**1.11.5. Disease/carriage phenotype analyses**

We used sizes = c(1:16) in the “rfe” function so that the function tries all possible solutions to find the optimal features for predicting whether an isolate is a disease or carriage isolate. The output indicates that RFE recommends 3 features for the best model where both accuracy (89%) and Kappa (0.73) reach the maximum level. The features selected by RFE are: AD.HI, LDH and fetA. Using the test dataset to check the model performance gave slightly lower accuracy (81%) and Kappa (0.52).


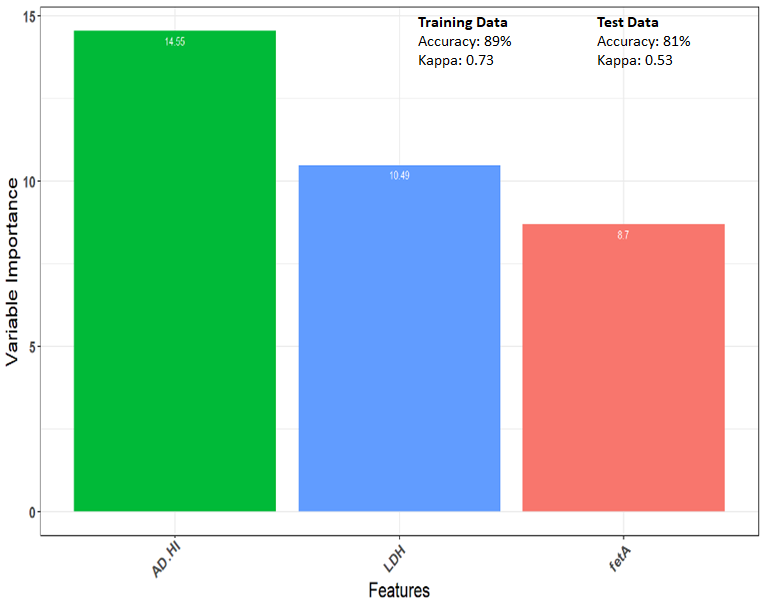
 Figure 1.11.3. Phenotypes, phasotypes and/or PV genes identified in predicting whether an isolate is a disease or carriage isolate.

**1.11.6. Phenotype predictions from group, Phasotypes and PV genes**

Root Mean Squared Error (RMSE)and Mean Absolute Error (MAE) are metrics used to evaluate the differences between the predicted values and the actual values of a variable. They are calculated as follows:

$$RMSE=\sqrt{\frac{\sum\left( y_{i}-y_{p} \right)^{2}}{n}}$$

$$MAE= \frac{\left| \left( y_{i}-y_{p} \right) \right|}{n}$$

Where;

$$y_{i}=actual value$$

$$y_{p}=predicted value$$

$$n=number of observations/rows$$

The lower the RMSE/MAE the better a model can predict sample outcomes. We used sizes = c(1:17) in the “rfe” function so that the function tries all possible solutions to find the optimal features. The BHI_k phenotype was best predicted by the pilC1 gene with a RMSE (0.1) and MAE (0.07) in the training data and RMSE (0.14) and MAE (0.07) in the test data. Models for predicting BHI_r, LDH, Adhesion and AD.HI all indicated group to be the best predictor for the model with the lowest RMSE (0.09, 14.63, 1.47e+08, and 0.85 respectively ) and MAE (0.06, 10.8, 1.03e+08, and 0.62 respectively). Using the test datasets to check the model performance gave the following RMSE (0.05, 13.3, 1.22e+08, and 0.8 respectively) and MAE (0.04, 10.7, 7.6e+07, and 0.57 respectively). SBAD phenotype was best predicted by the fetA gene with a RMSE (18.63) and MAE (15.41) in the training data and RMSE (19.32) and MAE (15.67) in the test data.

For the BHI_tmid phenotype, the output indicates that RFE recommends 4 features for the model with lowest RMSE (2.36) and MAE (1.66). The 4 features selected by RFE are: group, MOD, ADHESIN, and porA. Checking the model performance using the test dataset gave a RMSE (1.57) and MAE (1.25) respectively.


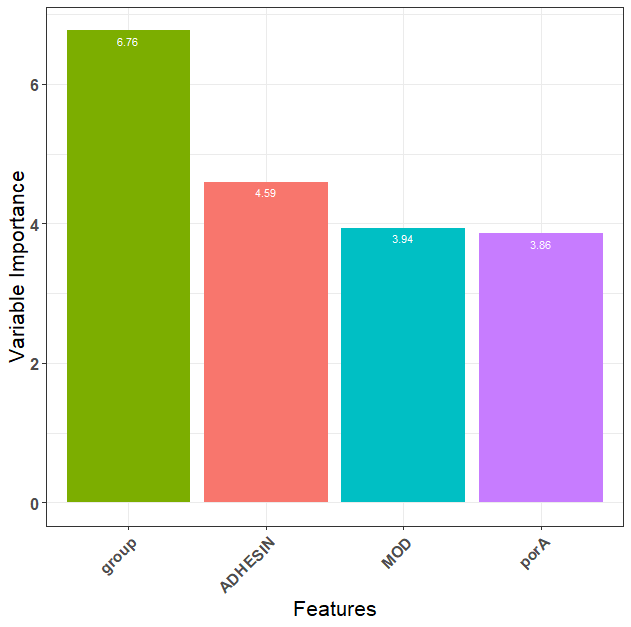
 Figure 1.11.4. Group, phasotypes and PV genes identified in predicting BHI_tmid phenotype.

For the RPMI_r phenotype, the output indicates that RFE recommends 3 features for the model with lowest RMSE (0.21) and MAE (1.15). The 3 features selected by RFE are: group, fetA, and pilC2. Checking the model performance using the test dataset gave a RMSE (0.24) and MAE (0.15) respectively.


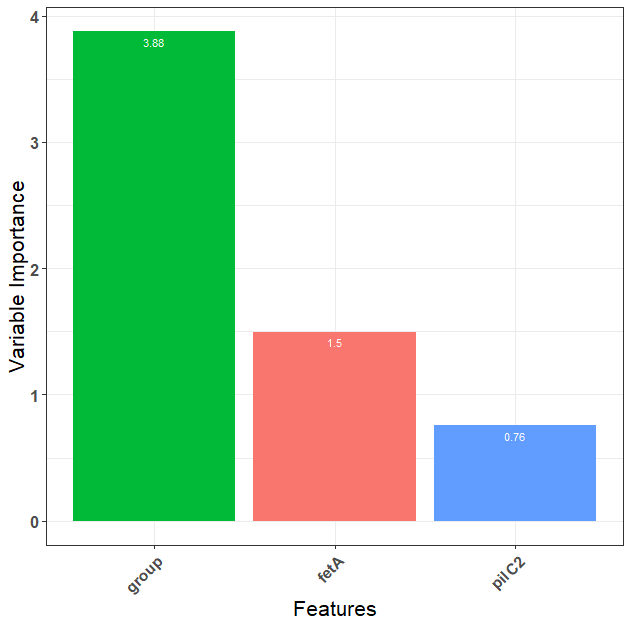
 Figure 1.11.5. Group and PV genes identified in predicting RPMI_r phenotype.

For the RPMI_tmid phenotype, the output indicates that RFE recommends 5 features for the model with lowest RMSE (3.68) and MAE (2.56). The 5 features selected by RFE are: group, hpuA, modA, mspA, and nalP. Checking the model performance using the test dataset gave a RMSE (7.85) and MAE (3.56) respectively.


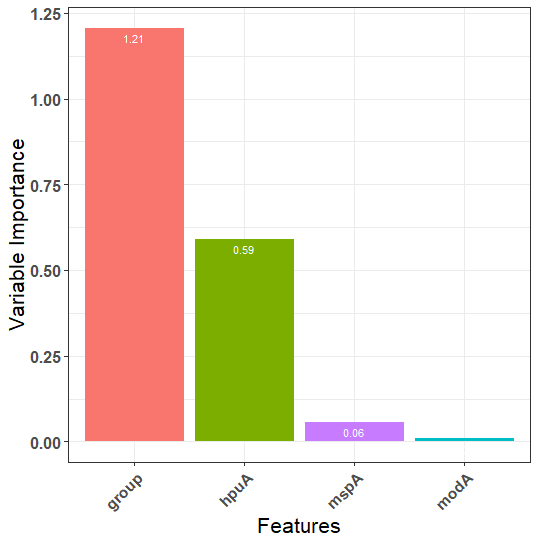


Figure 11.11.6. Group and PV genes identified in predicting RPMI_tmid phenotype.
