# Supplementary material for: High-throughput phenotype-to-genotype testing of meningococcal carriage and disease isolates detects genetic determinants of disease-relevant phenotypic traits
Source: mBio. 2024 Oct 30;15(12):e03059-24. doi: 10.1128/mbio.03059-24 (PMC11633189; doi:10.1128/mbio.03059-24)
Supplement: Data File S7 — Detailed methods. [file mbio.03059-24-s0007.docx]

**Details of phenotypic assays**

**Preparation of multi-assay stock plates.** As described in detail by Farzand *et al*. (1), master stocks of bacterial isolates were prepared in 96-well polypropylene non-pyrogenic plates (Corning Incorporated Coaster). Batches of plates were produced from a single mid-log phase culture. Briefly, strains were recovered from glycerol stocks by plating on supplemented BHI agar plates and incubated for ~18 hours. Colony sweeps were suspended into 2 mL BHI broth to OD580 of 0.05 and grown to mid-exponential phase (3-3.5 hr). Aliquots were transferred to 96-well plates containing 50% glycerol followed by sealing with an adhesive foil PCR seal.

**Growth Assay.** A 1:10 dilution was prepared from frozen multi-assay stock plates into BHI or Roswell Park Memorial Institute (RPMI) 1640 media (Gibco, USA) and incubated as above. Overnight cultures were diluted 1:10 into relevant media (final volume, 150 uL) in clear, flat-bottomed 96-well microtiter plates (Nunc, USA). Plates, sealed with a breath-easy membrane and lid, were incubated with shaking at 37°C, 5% CO_2_ in a BMG Labtech Omega FLUOstar plate reader. OD600 measurements were taken every 10 minutes for 48 hours and corrected relative to BHI broth controls. Using growthcurver (R package; (2)), logistic function curves were fitted to OD600 data for calculation of doubling time (r), lag phase (time to mid-log phase, Tmid) and maximum growth (k).

**Biofilm Assay.** Formation of biofilms on pegs (NuncTM Immuno TSP Lids) were performed as reported by Harrison *et al*. (3). Briefly, meningococcal strains were diluted 1:10 from stock plates into tryptic soy broth (TSB) medium in flat-bottomed 96-well plates ( final volume, 150 μL) and incubated overnight. Overnight cultures were diluted (1:3) into fresh TSB (100 μL) in 96-peg lid plates and incubated statically for 24 hrs. Peg lids were washed twice with 200 μL distilled water, stained with 1% crystal violet (CV) for 15 minutes at room temperature followed by two further washes in distilled water (4). CV was solubilised in a 30% acetic acid solution and quantified at OD550. Percentage CV values are relative to 100% for strain B141.

**Meningococcal lactate dehydrogenase (LDH) release assay.** Multi-assay stock plates were diluted 1:10 into RPMI 1640 media, incubated overnight, diluted 1:10 into fresh RPMI 1640 and incubated to mid-exponential phase (3-3.5 hr). Released LDH activity was measured in 50 μL aliquots of bacterial suspensions by mixing with 50 μL colourometric solution of a cytotoxicity detection kit (Sigma-Aldrich). Total LDH activity was quantified by treating bacterial suspensions with Triton X-100 (1% v/v). Background LDH activity values from culture media were subtracted from released and total values. Released LDH activity was reported as a % of total LDH activity.

**Adhesion.** Immortalised adenocarcinoma A594 (ATCC number: CCL185TM) cells were grown in RPMI media supplemented with 10% fetal bovine serum (FBS) (Gibco, USA) in 75 cm^2^ flasks (Greiner Bio-one, Austria) at 37^o^C, 5% CO_2_ to 70% confluency. A549 cells, recovered by 0.05% trypsin-EDTA (Gibco, USA) treatment for 5 minutes at 37^o^C, were seeded at 1x10^5^ cells/well into 48-well plates (Corning, USA) and incubated overnight at 37oC, 5% CO_2_. A549 cells were infected with exponentially-growing bacterial cells, prepared as for the LDH assay, at a multiplicity of infection (MOI) of 30 in 0.5 mL of RPMI-1640/10% FBS for 1 hour at 37°C, 5% CO_2_. Non-adherent bacteria were removed by two gentle washes with PBS followed by addition of 0.5 mL of RPMI-1640/10% FBS. Plates were incubated for 18 hours at 37°C, 5% CO_2_. After overnight incubation, non-adherent bacteria were removed by washing three times with PBS. Adherent bacterial cells were quantified by lysing infected monolayers with 0.1% saponin (Sigma, USA) for 20 minutes followed by plating aliquots (10 μL) of serial dilutions, prepared in PBS, in triplicate onto BHI agar plates for enumeration of colony forming units (CFU).

**Serum survival assays.** Serum bactericidal antibody-depleted assays (SBAD) were performed with IgG/IgM-depleted human sera (Pel-Freeze 34010 -5 HU Comp IgG/IgM Depleted Pooled) to minimise variation due to differing sensitivities to meningococcal-specific intrinsic antibodies. Pre-testing detected showed significant levels of killing with a 30% dilution of this serum. An antibody-depleted heat-inactivated (AD-HI) serum was prepared by incubation at 56^o^C for 30 min. Bacterial inocula, prepared as described for the LDH assay, were diluted in 1X Hanks' balanced salt solution (HBSS; Catalogue number: 14175137) to an OD600 1.0. For all isolates, aliquots of bacterial suspensions, containing 10^4^ CFU, were mixed with either active or HI AD-human sera in 1X HBSS buffer (total volume, 100 μL). Samples were incubated for 1 hr (T1) at 37^o^C, 5% CO_2_. Serial dilutions, prepared as above, for T0 (inoculum) and T1 samples were plated for CFU enumeration. SBAD activity was measured as the reduction in CFU counts in active versus HI AD-serum as follows:- % of relative reduction = 100 – (CFU at T1 in active AD-sera/average CFU at T1 in AD-HI serum)*100. Survival in AD-HI serum was calculated as follows:- % of AD-HI serum survival = [CFU at T1/CFU at T0 in inactivated serum]*100.

References

1. Farzand R, Croix MS, Dave N, Bayliss CD. 2023. Development of a robust and quantitative high-throughput screening method for assessing phenotypic variation in large Neisseria meningitidis isolate collections. MethodsX 10:102091.

2. Sprouffske K, Wagner A. 2016. Growthcurver: an R package for obtaining interpretable metrics from microbial growth curves. BMC Bioinformatics 17:172.

3. Harrison JJ, Stremick CA, Turner RJ, Allan ND, Olson ME, Ceri H. 2010. Microtiter susceptibility testing of microbes growing on peg lids: a miniaturized biofilm model for high-throughput screening. Nat Protoc 5:1236-54.

4. O'Toole GA. 2011. Microtiter dish biofilm formation assay. J Vis Exp doi:10.3791/2437.
