# Supplementary material for: High-throughput phenotype-to-genotype testing of meningococcal carriage and disease isolates detects genetic determinants of disease-relevant phenotypic traits
Source: mBio. 2024 Oct 30;15(12):e03059-24. doi: 10.1128/mbio.03059-24 (PMC11633189; doi:10.1128/mbio.03059-24)
Supplement: Supplemental Figures — Figures S1 to S29. [file mbio.03059-24-s0009.docx]

**List and brief description of Appendix Figures**

**Figure S1. Phylogenetic association of MenW:cc11 isolates selected for the phenotype-to-genotype study with worldwide cc11 isolates.** This figure shows the phylogenetic relationships between the UK MenW:cc11 isolates analysed in this study and cc11 of isolates with other genogroups and from other geographic regions.

**Figure S2. Flow chart of high throughput phenotypic assays and genotype analyses.** This figure depicts a flow chart for the high throughput phenotyping assays and genotyping analyses.

**Figure S3. Association of source or phylogenetic group with phenotypic variation of MenW:cc11 UK disease and carriage isolates.** This figure shows a correlation analysis for the 11 phenotypic traits.

**Figure S4. Association of phenotypic score with phylogenetic lineage of MenW cc11 isolates.** This figure shows how variance in the phenotypes correlates with the phylogenetic lineages of our isolates.

**Figure S5. Association of minor csw alleles with specific phenotypes.** This figure shows the phenotypic variance of all isolates and separately carriage isolates containing different alleles of the *csw* gene that encodes a product essential for synthesis of the MenW capsule.

**Figure S6. Position of variation in csw gene sequence and distribution of indel mutations across the phylogenetic lineages of the MenW cc11 strains.** This figure shows the positions and variants in the *csw* gene sequences for all alleles present in this gene and how indels and minor variants in the *csw* gene are distributed across the phylogenetic tree of the isolates utilised in this study.

**Figure S7. Association between unitigs and phenotypes (beta).** This figure shows examples of the data values obtained for isolates that contain or lack a specific unitig found to be significant in the GWAS analysis for a particular phenotype.

**Figure S8. Unitig-isolate distributions on the phylogeny tree.** This figure shows how unitigs are distributed across the phylogenetic tree.

**Figure S9. Distribution of isolates among different phase variable (PV) gene expression states and phasotypes.** This graph shows how the different PV states and phasotypes are distributed among the three isolate groups (i.e. Original_invasive, 2013_invasive, carriage).

The next 10 graphs show association between individual gene expression states and phenotype values for PV genes subject to either ON/OFF or High/Int/Low phase variation.

**Figure S10. Association between hmbR PV states and phenotypic variation in MenW cc11 isolates.**

**Figure S11. Association between porA PV states and phenotypic variation in MenW cc11 isolates.**

**Figure S12. Association between fetA PV states and phenotypic variation in MenW cc11 isolates.**

**Figure S13. Association between nadA PV states and phenotypic variation in MenW cc11 isolates.**

**Figure S14. Association between hpuA PV states and phenotypic variation in MenW cc11 isolates.**

**Figure S15. Association between mspA PV states and phenotypic variation in MenW cc11 isolates.**

**Figure S16. Association between nalP PV states and phenotypic variation in MenW cc11 isolates.**

**Figure S17. Association between pilC2 PV states and phenotypic variation in MenW cc11 isolates.**

**Figure S18. Association between modA PV states and phenotypic variation in MenW cc11 isolates.**

**Figure S19. Association between modB PV states and phenotypic variation in MenW cc11 isolates.**

**Figure S20. Distribution and correlation of PV genes with group in MenW cc11 isolates.** This graph shows a statistical analysis of the association between PV state and group.

The next 5 graphs show association between phasotypes (combinatorial PV expression states) and phenotype values.

**Figure S21. Association between PILIN phasotype and phenotypic variation in MenW cc11 isolates.**

**Figure S22. Association between AUTO phasotype and phenotypic variation in MenW cc11 isolates.**

**Figure S23. Association between MOD phasotype and phenotypic variation in MenW cc11 isolates.**

**Figure S24. Association between IRON phasotype and phenotypic variation in MenW cc11 isolates.**

**Figure S25. Association between ADHESIN phasotype and phenotypic variation in MenW cc11 isolates.**

**Figure S26. Distribution and correlation of phasotypes with group in MenW cc11 isolates.** This figure show a statistical test for the distribution of different phasotypes between the three isolate groups.

**Figure S27. Correlation matrix for phasotypes, all phase-variable genes and phenotypes.** This figure shows correlations between phasotypes, individual PV genes and phenotypes.

**Figure S28. Genes and phasotypes identified in predicting biofilm formation.** This figure shows a random forest model for predicting biofilm formation from PV genes and phasotypes.

**Figure S29. Genes and phasotypes identified in predicting RPMI_k.** This figure shows a random forest model for predicting maximum growth in RPMI media from PV gene and phasotypes.

**
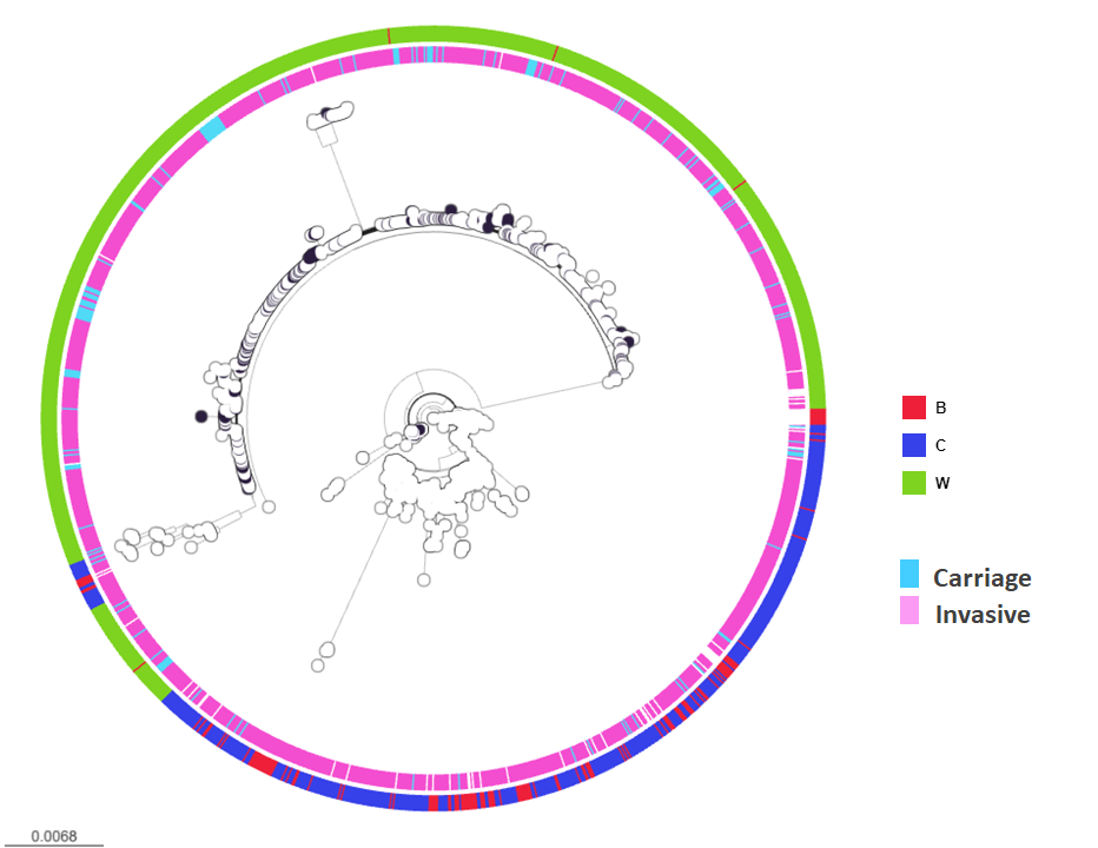
**

**Figure S1. Phylogenetic association of MenW:cc11 isolates selected for the phenotype-to-genotype study with worldwide cc11 isolates.** Phylogenetic tree representing the worldwide distribution and capsular serogroups of the cc11 lineage. This tree was reconstructed using the ML algorithm in RAxML, based on a gene-by-gene sequence alignment of 2,003 genes present in >90% of all isolates. A total of 1,492 genomes were utilised (128, MenB; 467, MenC; 897, MenW). Isolates utilised in the current study are indicated by black dots. Inner ring, invasive (pink)/carriage (light blue) designations. Outer ring, serogroup (MenB, red; MenC, dark blue; MenW, light green.

**
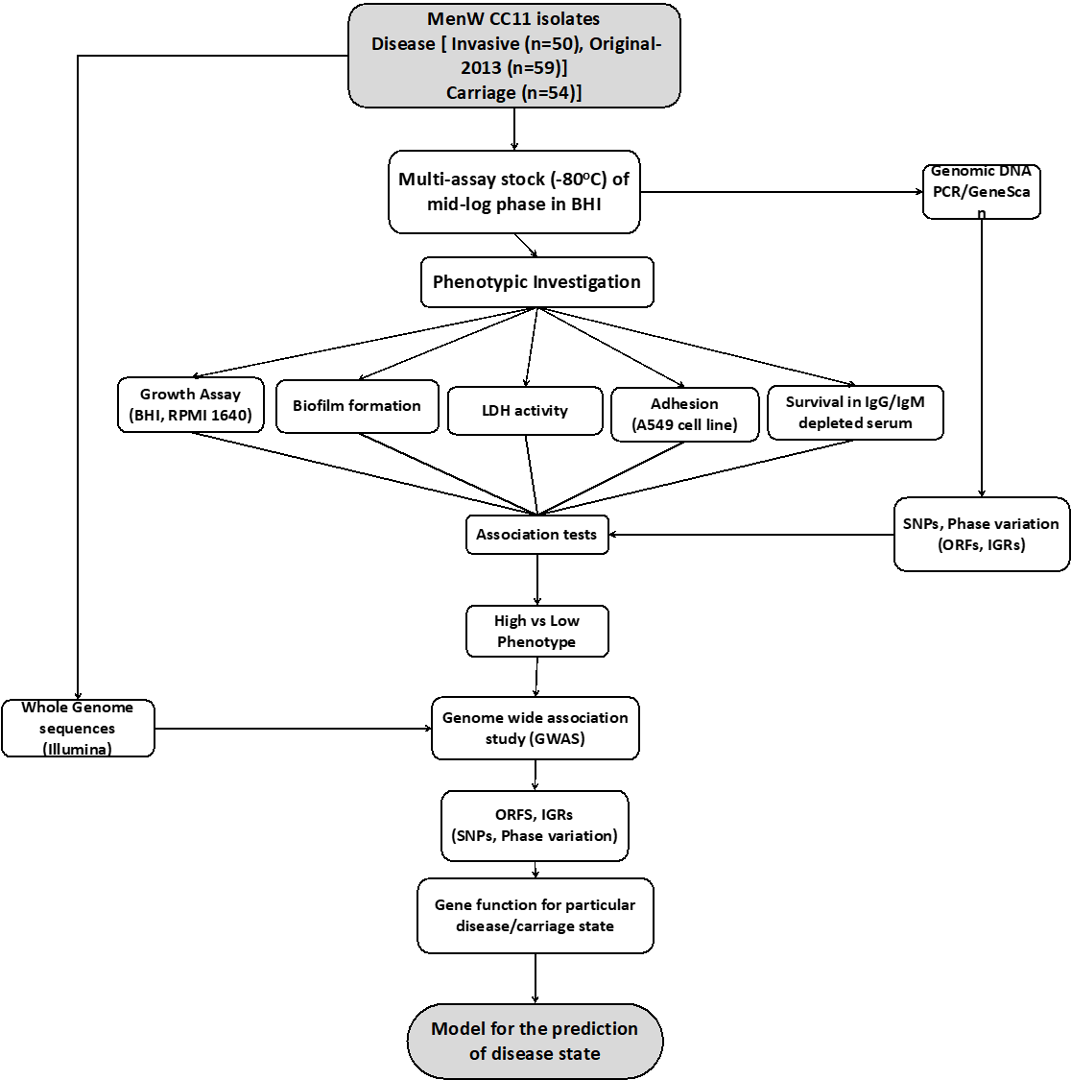
**

**Figure S2. Flow chart of high throughput phenotypic assays and genotype analyses.** This diagram depicts the series of steps for performing the phenotypic screening and genetic associations. The first step involved generation of multi-assay stock plates from multiple individual frozen stocks. PV states of the most variable PV genes were determined by PCR and GeneScan analysis with DNA extracts from these plates. Five phenotypic assays were performed utilising the stock plates leading to generation of data sets for eleven phenotype traits. Association tests were performed with these data sets for isolate type (invasive original, invasive 2013, carriage), PV state and phasotype. GWAS tests were performed with whole genome sequence data and continuous phenotypic data for each trait for the whole isolate collection.


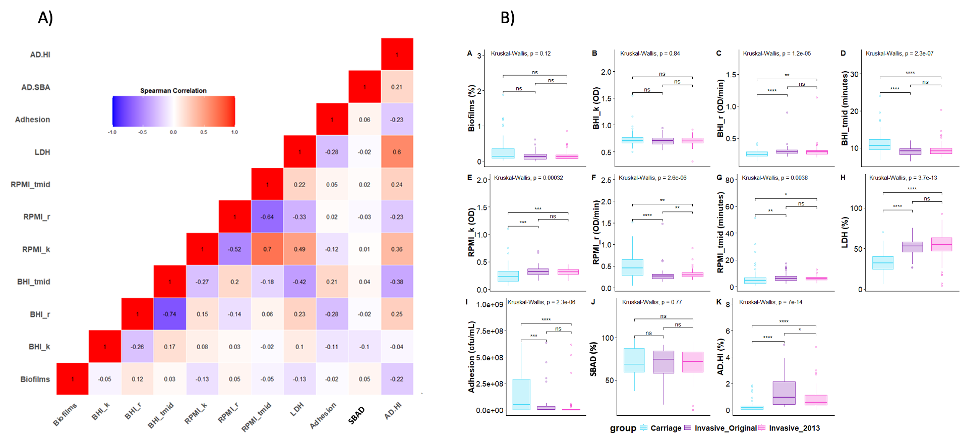


**Figure S3. Analysis for correlations between phenotypic variation of 163 MenW:cc11 UK disease and carriage isolates.** Data were obtained for 11 phenotypic traits from six assays for 163 isolates of MenW:cc11 lineage. These data were tested for positive and negative correlations between traits. Phenotypic assays and traits:-Biofilms, biofilm formation measured as % of crystal violet staining relative to isolate B141; growth assays in BHI or RPMI media with three traits - k, OD for maximum growth plateau, r, replication rate in OD/min, T-mid, minutes to mid-log phase); LDH, released lactate dehydrogenase activity as % of total LDH activity from lysed control cells; Adhesion, adhesion to A549 cells (cfu/mL); SBAD, serum resistance measured as a % of CFU counts following incubation in antibody-depleted serum versus heat-inactivated antibody-depleted serum; AD-HI, sensitivity to heat-inactivated serum measured as % of CFU counts following incubation in heat-inactivated antibody-depleted serum versus inoculum). Correlations were assessed using a Spearman’s Rho correlation test. The correlation coefficient is interpreted as: positive (+); negative (-); perfect (1); very strong (0.8 -0.9); moderate (0.6 - 0.7); fair (0.3 - 0.5);poor (0.1 - 0.2); none (0).

**
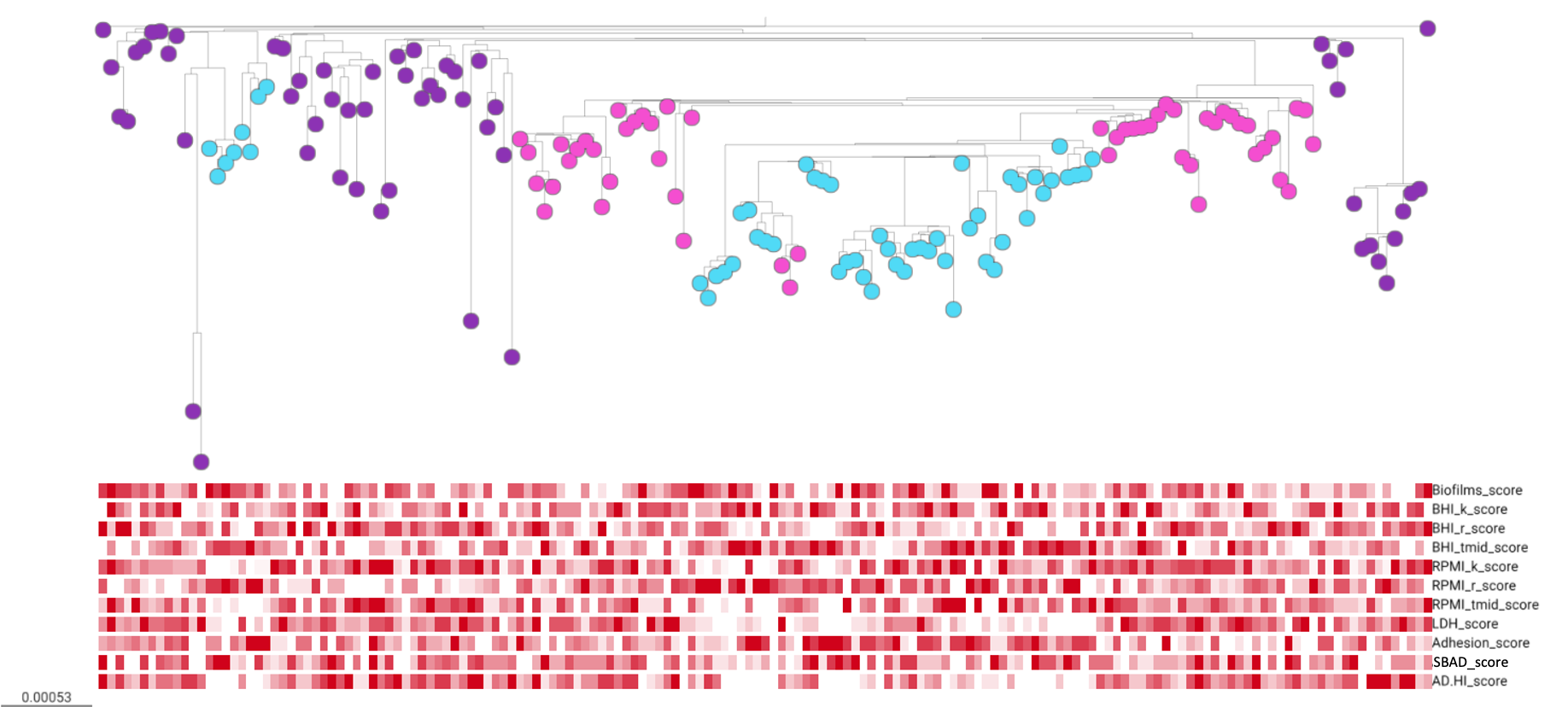
**

**Figure S4. Mapping of phenotypic score with phylogenetic lineage of MenW cc11 isolates.** Phylogenetic tree for the 163 MenW:cc11 isolates (purple dots, invasive, original isolates; pink dots, invasive, 2013 isolates; light blue dots, carriage isolates) utilised in the current study with mapped scores for individual phenotypes. Colour intensity from white to red indicate low to high percentage scores respectively.


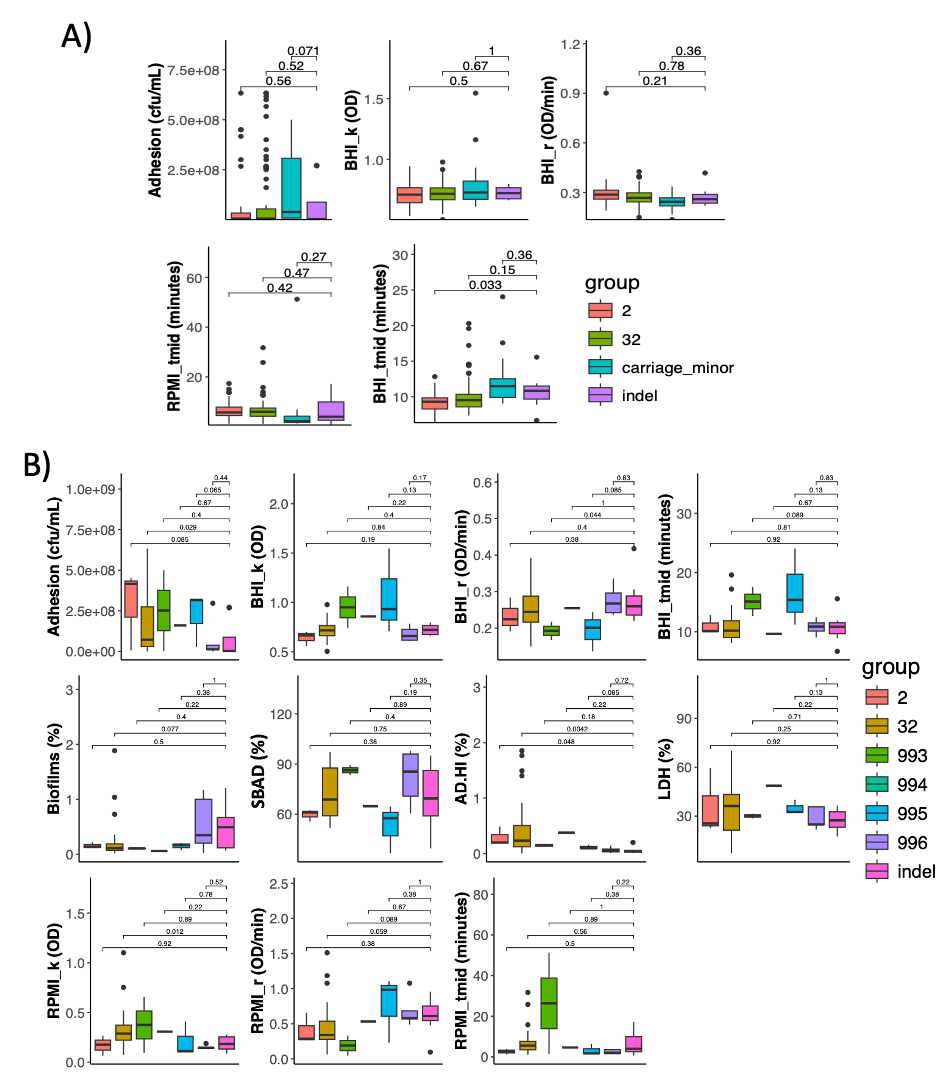


**Figure S5. Association of minor csw alleles of all isolates and carriage isolates with specific phenotypes.** Allele types for the csw gene were obtained for the disease and carriage isolates from the Neisseria PubMLST database. The majority of carriage isolates contained allele 32 (n=28), allele 2 (n=3) or other novel minor alleles with either single nucleotide differences (n=11) or indel mutations (n=8). A one-way ANOVA was applied across each of these seven groupings for all phenotypes; p values for comparison of the indel group to all other groups are provided. Panel A shows the associations of five phenotypes for all isolates with csw allele groups (note that the other six phenotypes are in Figure 3 of the main text). These isolates were separated according to the csw alleles into four groups:- major allele 2; major allele 32; minor non-indel alleles (carriage minor); and indel alleles. Panel B shows the associations for carriage isolates only and separates the minor alleles into four specific alleles (i.e. 993, 994, 995 and 996).


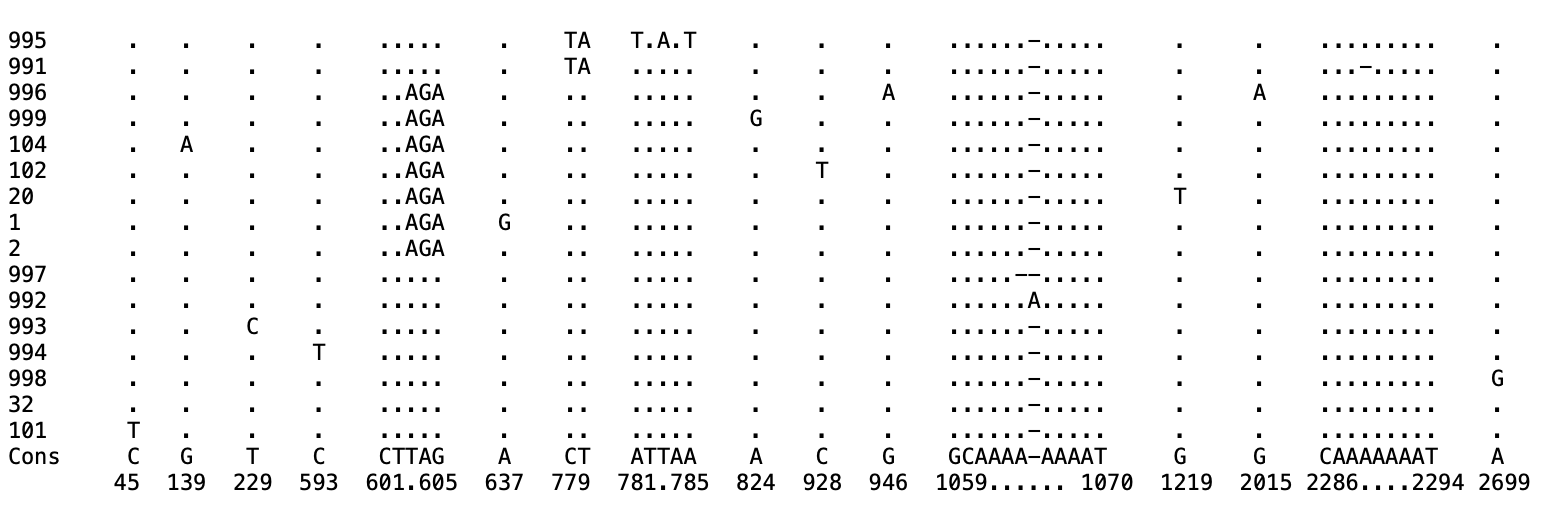


**Figure S6. Position of variation in csw gene sequence and distribution of indel mutations across the phylogenetic lineages of the MenW cc11 strains.** The top panel shows the positions of the variation in *csw* gene sequence. This gene has a total length of 3,114 bps and positions are relative to this sequence. The sequences surrounding the indels are provided. Allele 991 and 997 have 1 nt deletions and allele 992 has 1 nt insertion with all these indels located in A tracts of 7 or 8 nucleotides. Cons, consensus sequence. The bottom panel shows a phylogenetic tree that was made using the PubMLST iTOL plugin with the ‘All loci’ scheme. 0, isolates where no allele at the csw locus was detected; 1, isolates with non-indel alleles; 991, 992, 997, the three indel alleles.


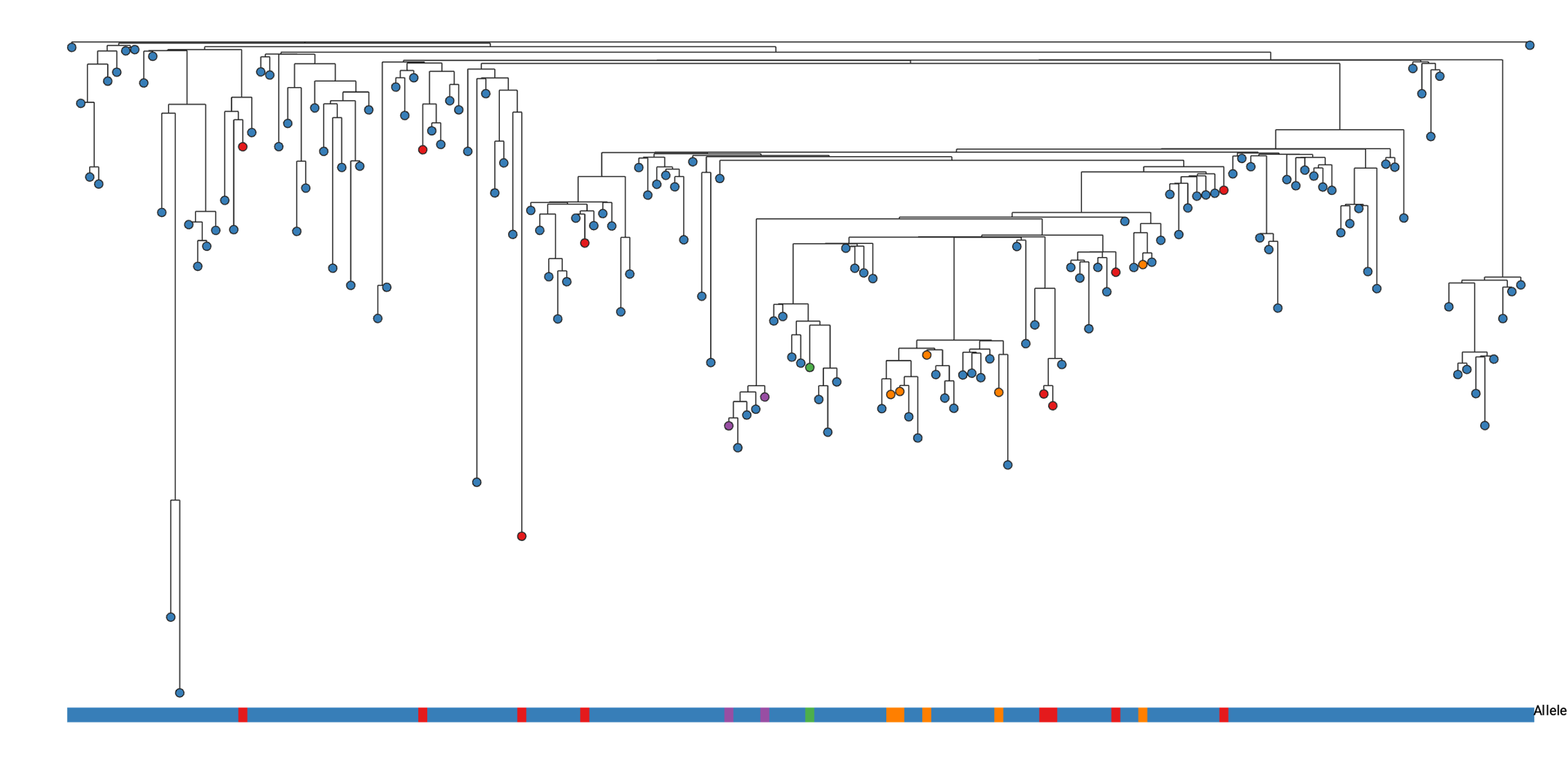

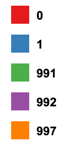


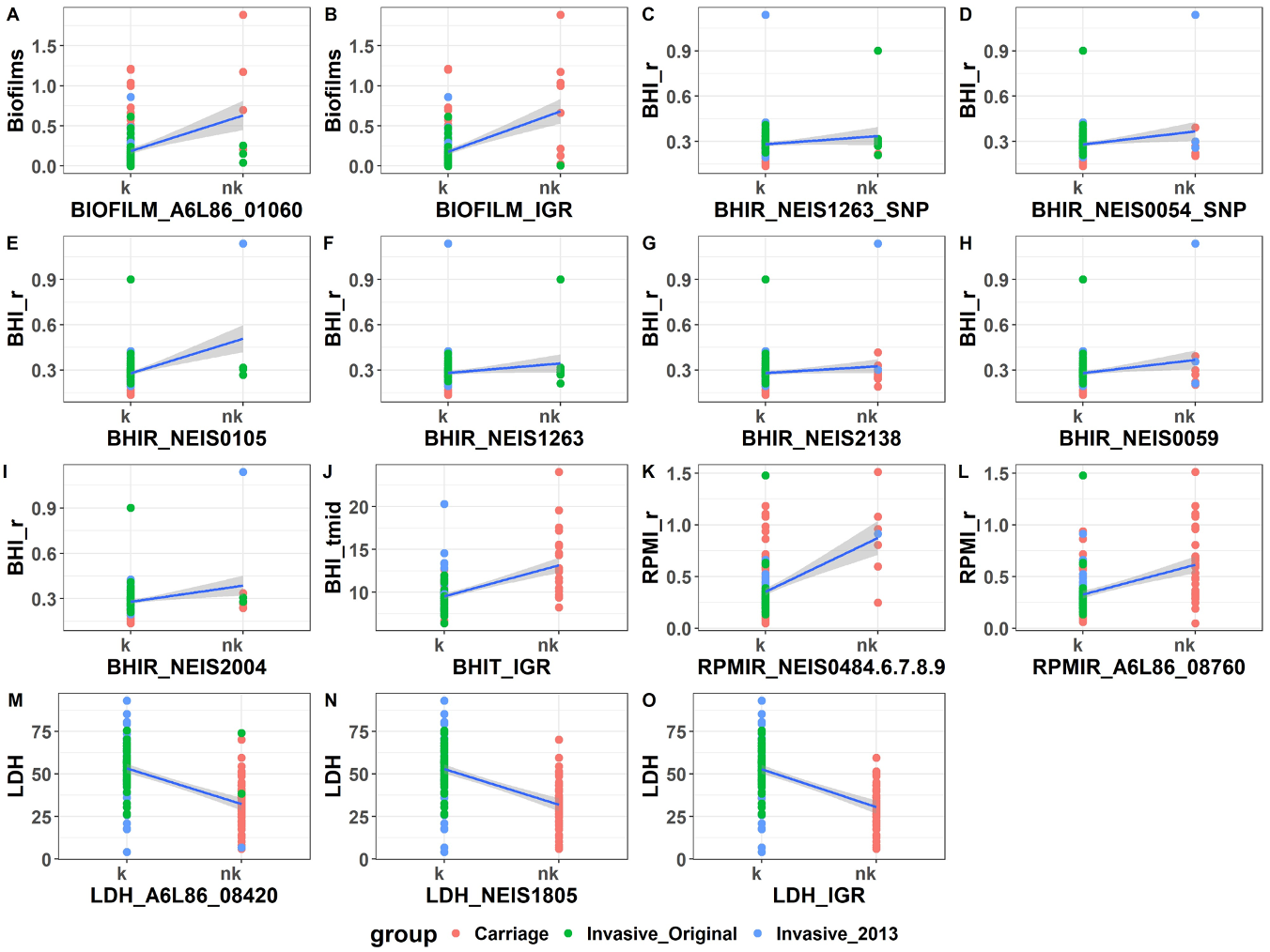


**Figure S7. Association between unitigs and phenotypes (beta).** Visualization of the association (beta) between unitig and phenotype as output from GWAS. Unitigs plotted were chosen from the lowest p-value (LP) and unitigs that had a non-synonymous substitution in the amino acid for respective phenotypes. The selected unitigs must have more than two isolates having the alternate unitig.


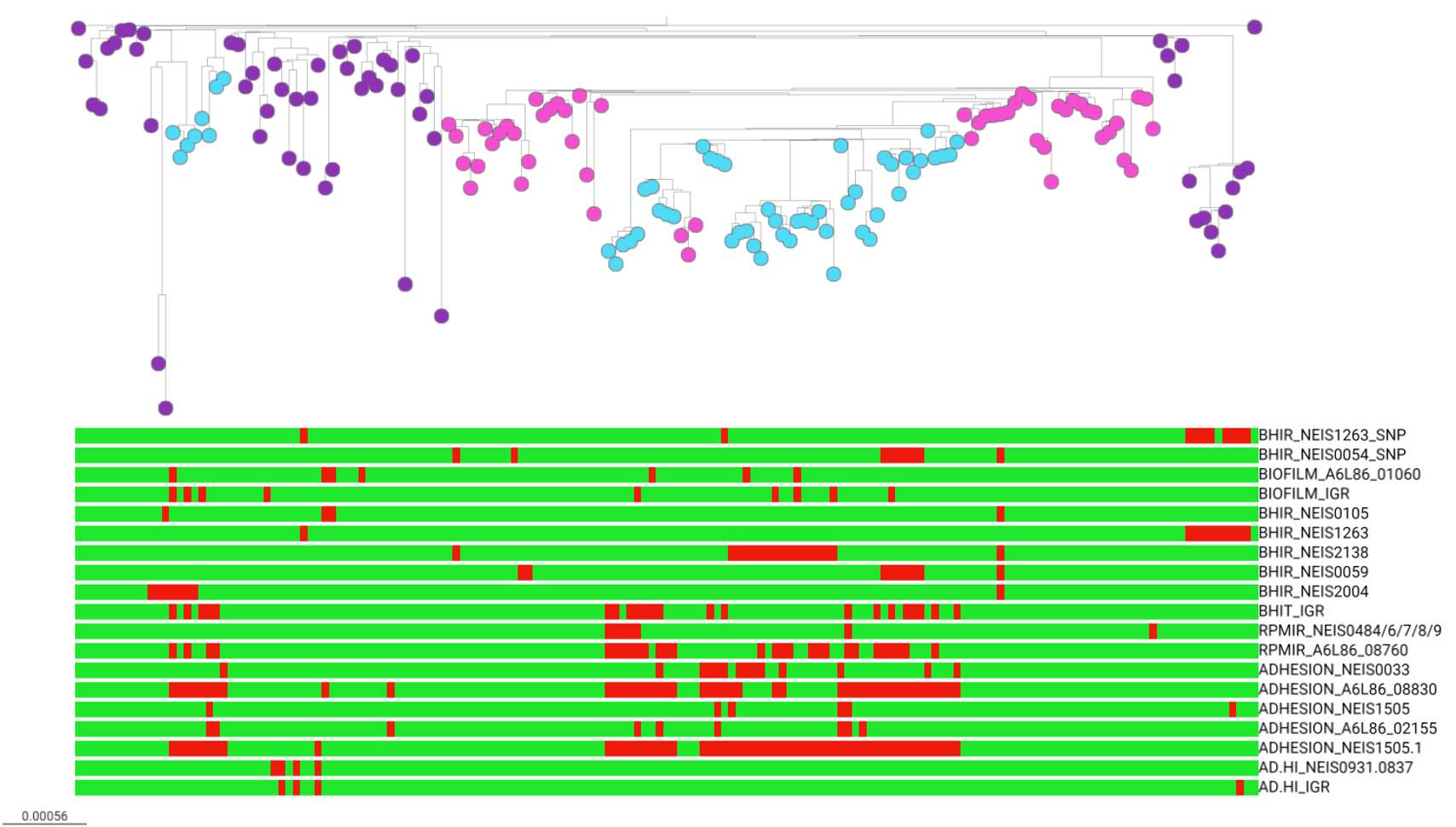


**Figure S8. Unitig-isolate distributions on the phylogeny tree.** Phylogenetic tree for the 163 MenW:cc11 isolates (purple dots, invasive, original isolates; pink dots, invasive, 2013 isolates; light blue dots, carriage isolates) utilised in the current study with selected GWAS unitigs mapped for some phenotypes. Green represents isolates that have the GWAS unitig (k) while red represent isolates that have an alternative unitig (nk).


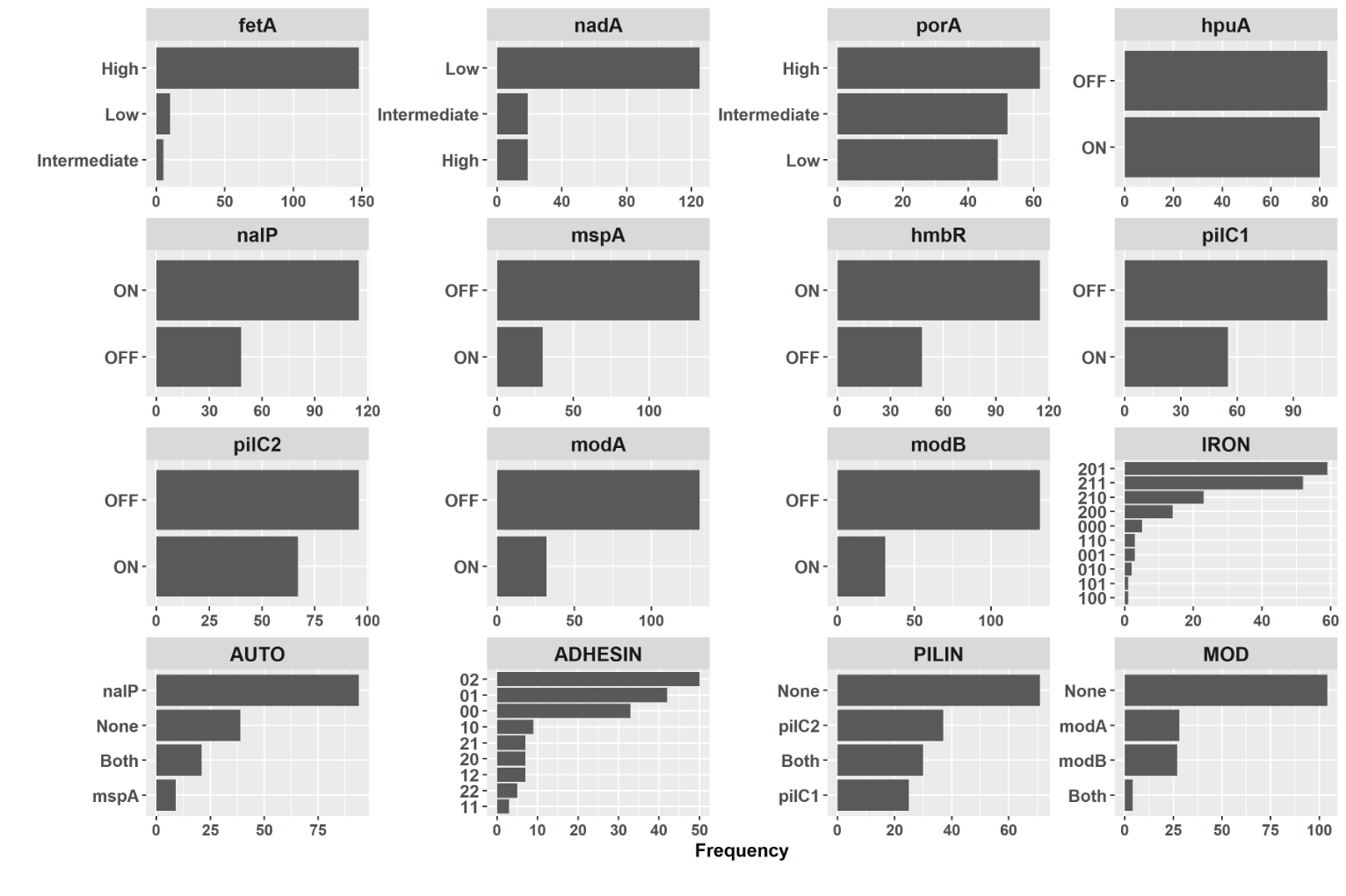


**Figure S9. Distribution of isolates among different phase variable (PV) gene expression states and phasotypes.** Three genes contain repeat tracts within the promoter and were allocated to high, intermediate or low expression states based on published correlations between repeat number and expression level. All other genes had repeat tracts within the reading frame and gene expression was predicated as ON or OFF depending on translation of the gene sequence. For the phasotypes, the expression states were coded as follows:- high, 2; intermediateor ON, 1; low of OFF, 0. The phasotypes are comprised of the following genes:- IRON, fetA, hpuA, and hmbR; AUTO, nalP and mspA; ADHESIN, nadA and porA; PILIN, pilC1 and pilC2; MOD, modA and modB.


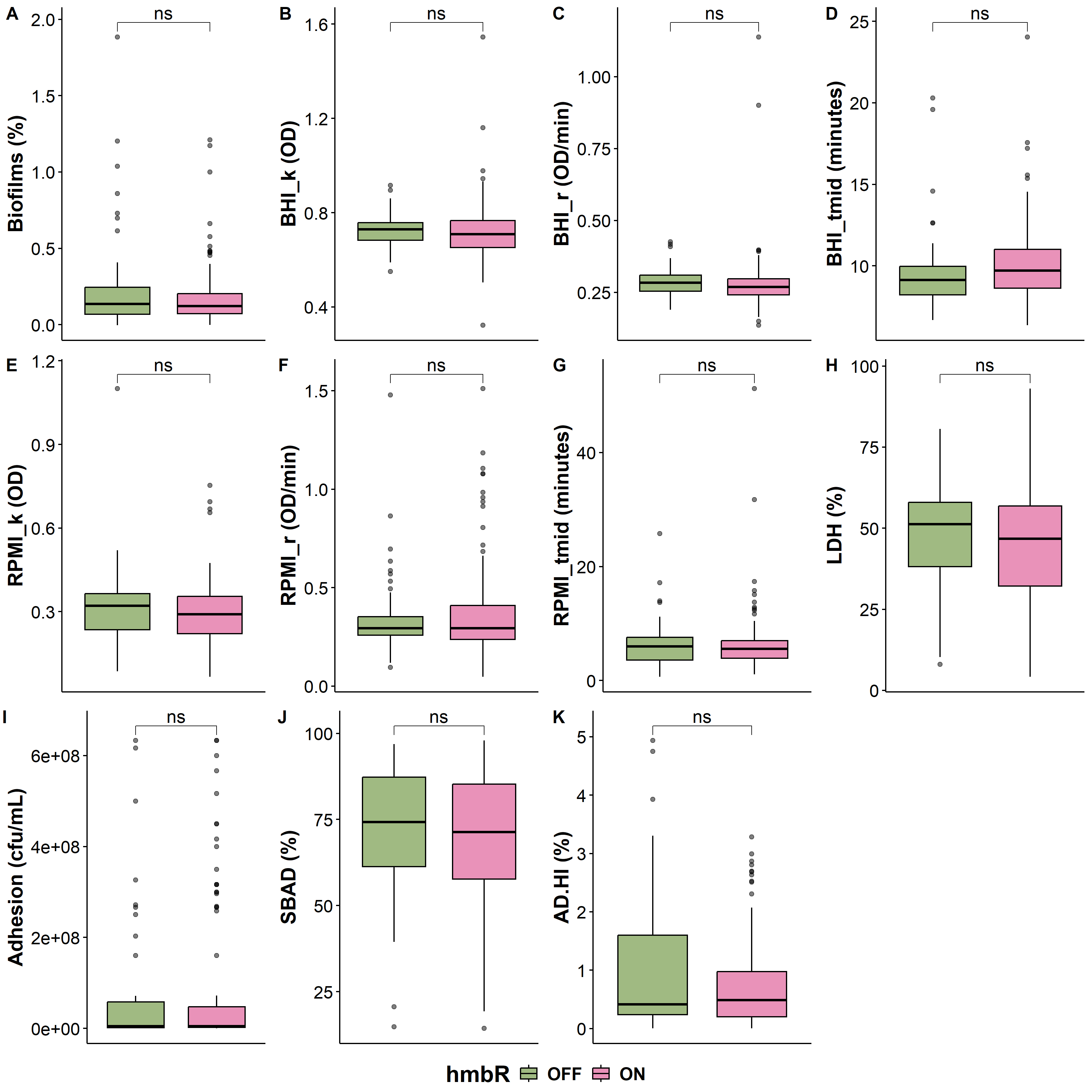


**Figure S10. Association between hmbR PV states and phenotypic variation in MenW cc11 isolates.** The expression states of this PV gene were determined for the 163 isolates by a combination of GeneScan and genomic analyses of the repeat tracts. The repeat tracts are located in the reading frame of this gene resulting in ON/OFF expression states. Phenotypic values were compared for isolates grouped by expression state using a Kruskal Wallis and Wilcoxon Rank-Sum test. No significance was detected in the Wicoxon test for any of the phenotypes at p<0.05. Plots: Bar, median; box, interquartile range; line, minimum and maximum; dots, outliers. P values: *, P<0.05; **, P<0.01; ***, P<0.001; ns, not significant.


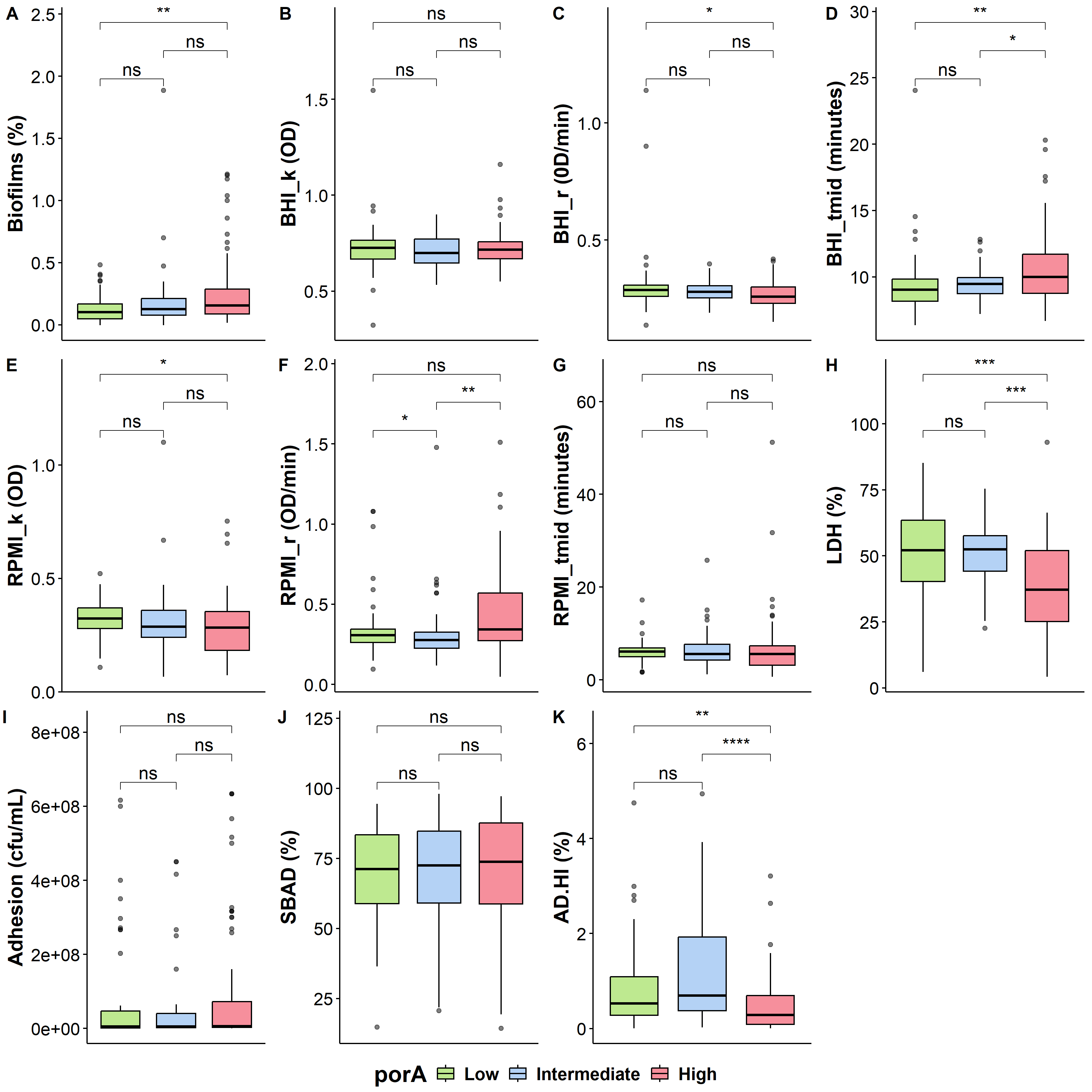


**Figure S11. Association between porA PV states and phenotypic variation in MenW cc11 isolates.** The expression states of this PV gene were determined for the 163 isolates by a combination of GeneScan and genomic analyses of the repeat tracts. The repeat tracts are located in the promoter of this gene resulting in High, Intermediate, or Low expression states. Phenotypic values were compared for isolates grouped by expression state using a Kruskal Wallis and Wilcoxon Rank-Sum test. The Wilcoxon test detected significance for biofilms (p=0.017), BHI_tmid (p=0.04), RPMI_r (p=0.0042), LDH (p=8x10^-5^), and AD-HI (p=1x10^-4^). Plots: Bar, median; box, interquartile range; line, minimum and maximum; dots, outliers. P values: *, P<0.05; **, P<0.01; ***, P<0.001; ns, not significant.


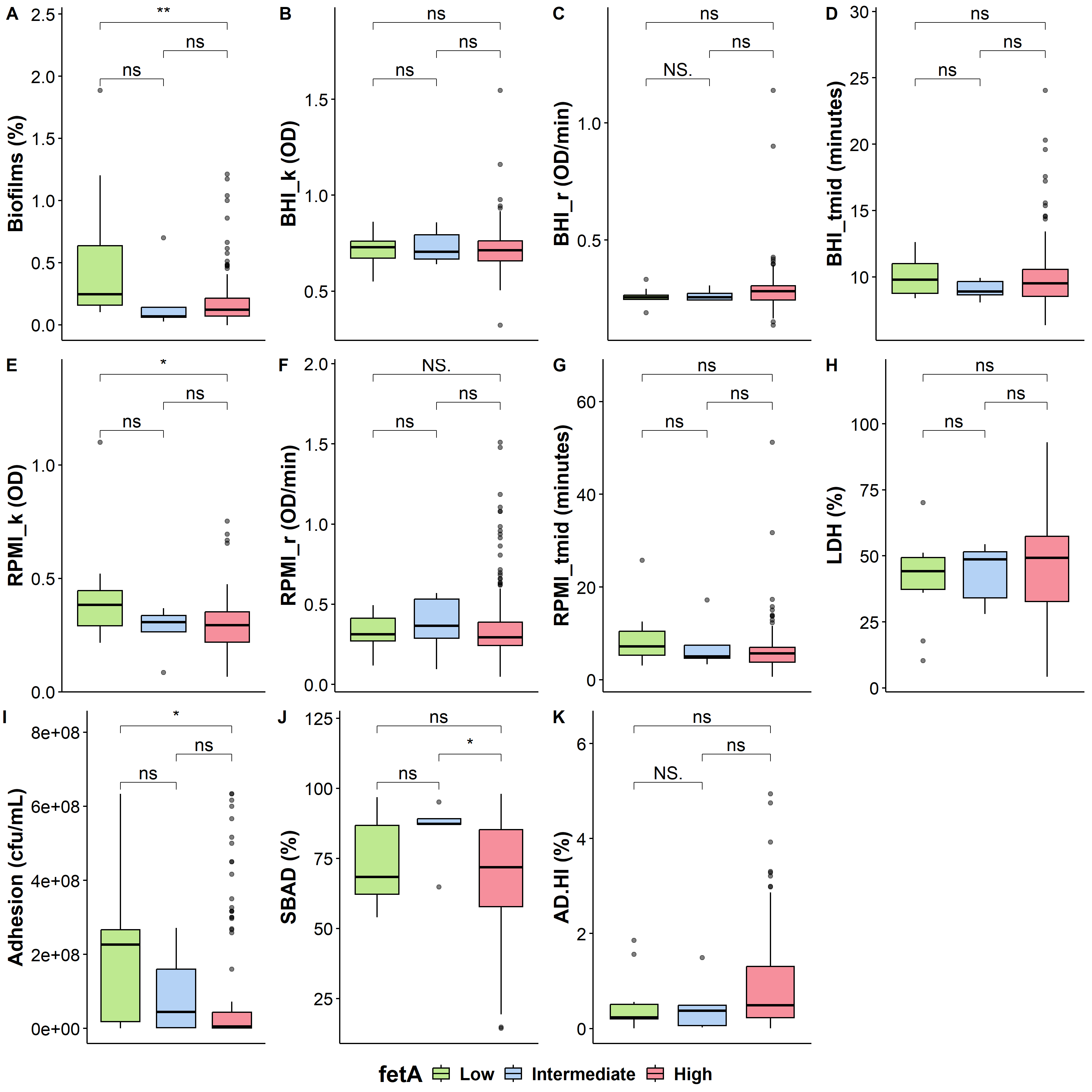


**Figure S12. Association between fetA PV states and phenotypic variation in MenW cc11 isolates.** The expression states of this PV gene were determined for the 163 isolates by a combination of GeneScan and genomic analyses of the repeat tracts. The repeat tracts are located in the promoter of this gene resulting in High, Intermediate, or Low expression states. Phenotypic values were compared for isolates grouped by expression state using a Kruskal Wallis and Wilcoxon Rank-Sum test. The Wilcoxon test detected significance for the biofilm trait (p=0.02) only. Plots: Bar, median; box, interquartile range; line, minimum and maximum; dots, outliers. P values: *, P<0.05; **, P<0.01; ***, P<0.001; ns, not significant.


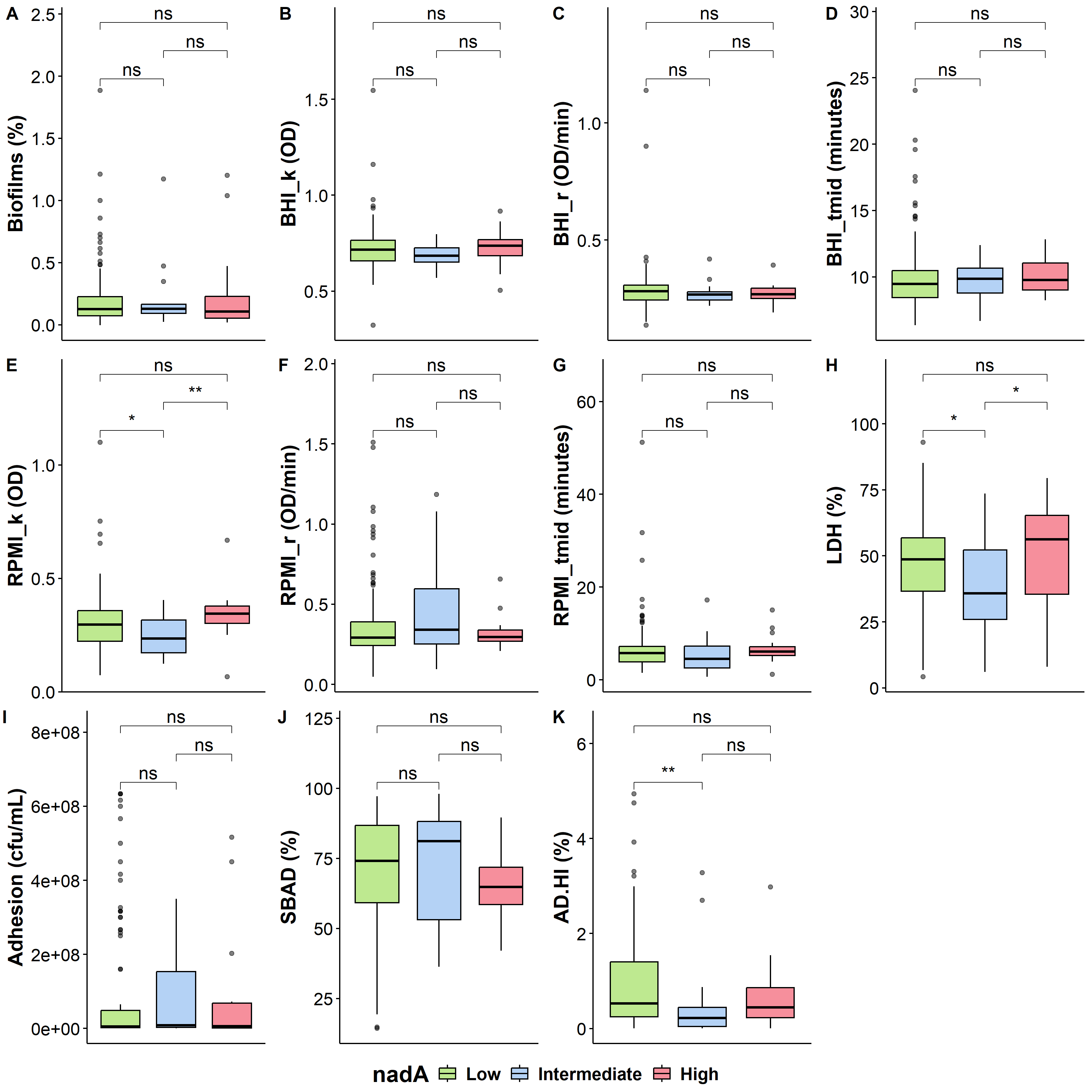


**Figure S13. Association between nadA PV states and phenotypic variation in MenW cc11 isolates.** The expression states of this PV gene were determined for the 163 isolates by a combination of GeneScan and genomic analyses of the repeat tracts. The repeat tracts are located in the promoter of this gene resulting in High, Intermediate, or Low expression states. Phenotypic values were compared for isolates grouped by expression state using a Kruskal Wallis and Wilcoxon Rank-Sum test. The Wilcoxon test detected significance for RPMI_k (p=0.014), and AD-HI (p=0.012). Plots: Bar, median; box, interquartile range; line, minimum and maximum; dots, outliers. P values: *, P<0.05; **, P<0.01; ***, P<0.001; ns, not significant.


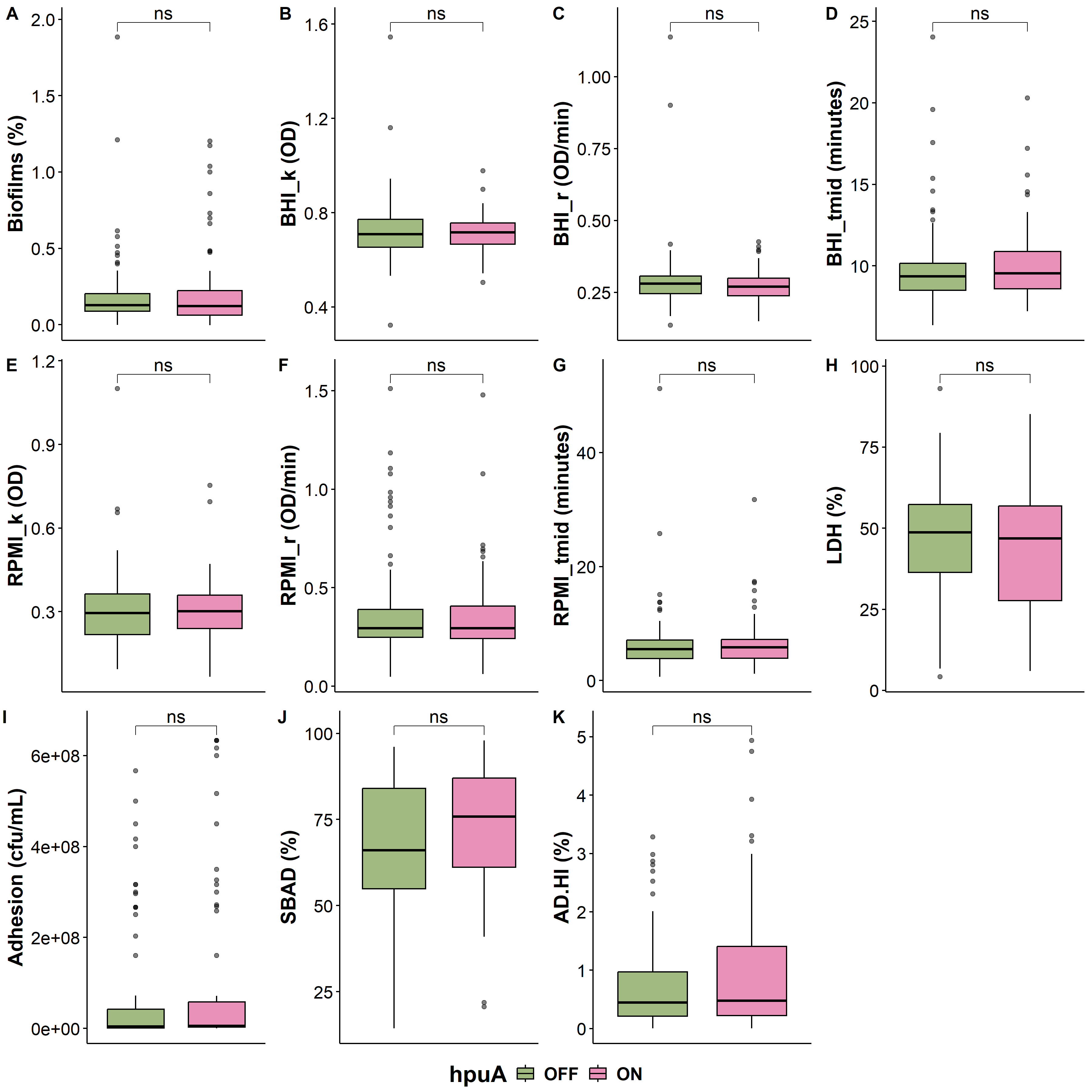


**Figure S14. Association between hpuA PV states and phenotypic variation in MenW cc11 isolates.** The expression states of this PV gene were determined for the 163 isolates by a combination of GeneScan and genomic analyses of the repeat tracts. The repeat tracts are located in the reading frame of this gene resulting in ON/OFF expression states. Phenotypic values were compared for isolates grouped by expression state using a Kruskal Wallis and Wilcoxon Rank-Sum test. The Wilcoxon test did not detect significance for any of the phenotypes at p<0.05. Plots: Bar, median; box, interquartile range; line, minimum and maximum; dots, outliers. P values: *, P<0.05; **, P<0.01; ***, P<0.001; ns, not significant.


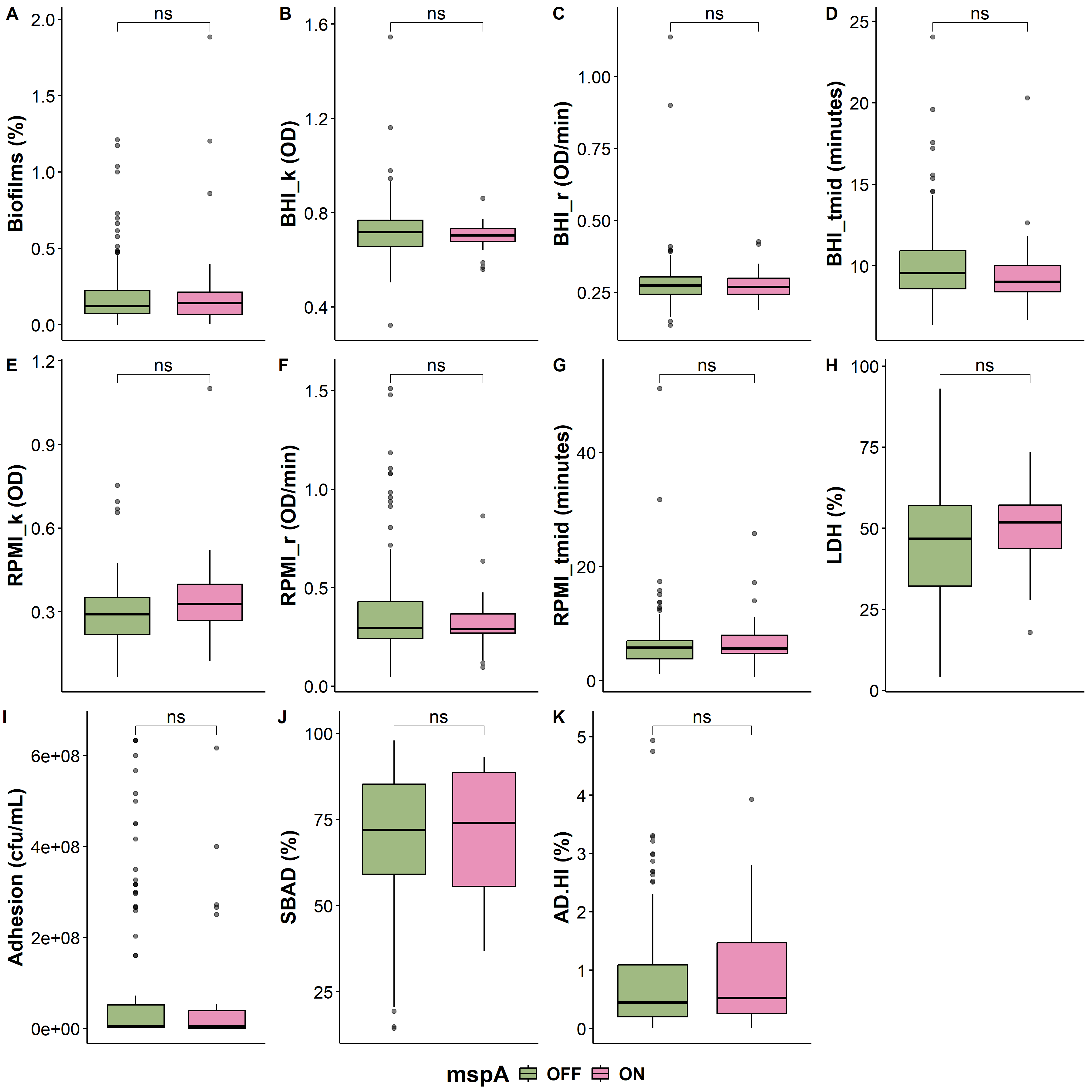


**Figure S15. Association between mspA PV states and phenotypic variation in MenW cc11 isolates.** The expression states of this PV gene were determined for the 163 isolates by a combination of GeneScan and genomic analyses of the repeat tracts. The repeat tracts are located in the reading frame of this gene resulting in ON/OFF expression states. Phenotypic values were compared for isolates grouped by expression state using a Kruskal Wallis and Wilcoxon Rank-Sum test. The Wilcoxon test did not detect significance for any of the phenotypes at p<0.05. Plots: Bar, median; box, interquartile range; line, minimum and maximum; dots, outliers. P values: *, P<0.05; **, P<0.01; ***, P<0.001; ns, not significant.


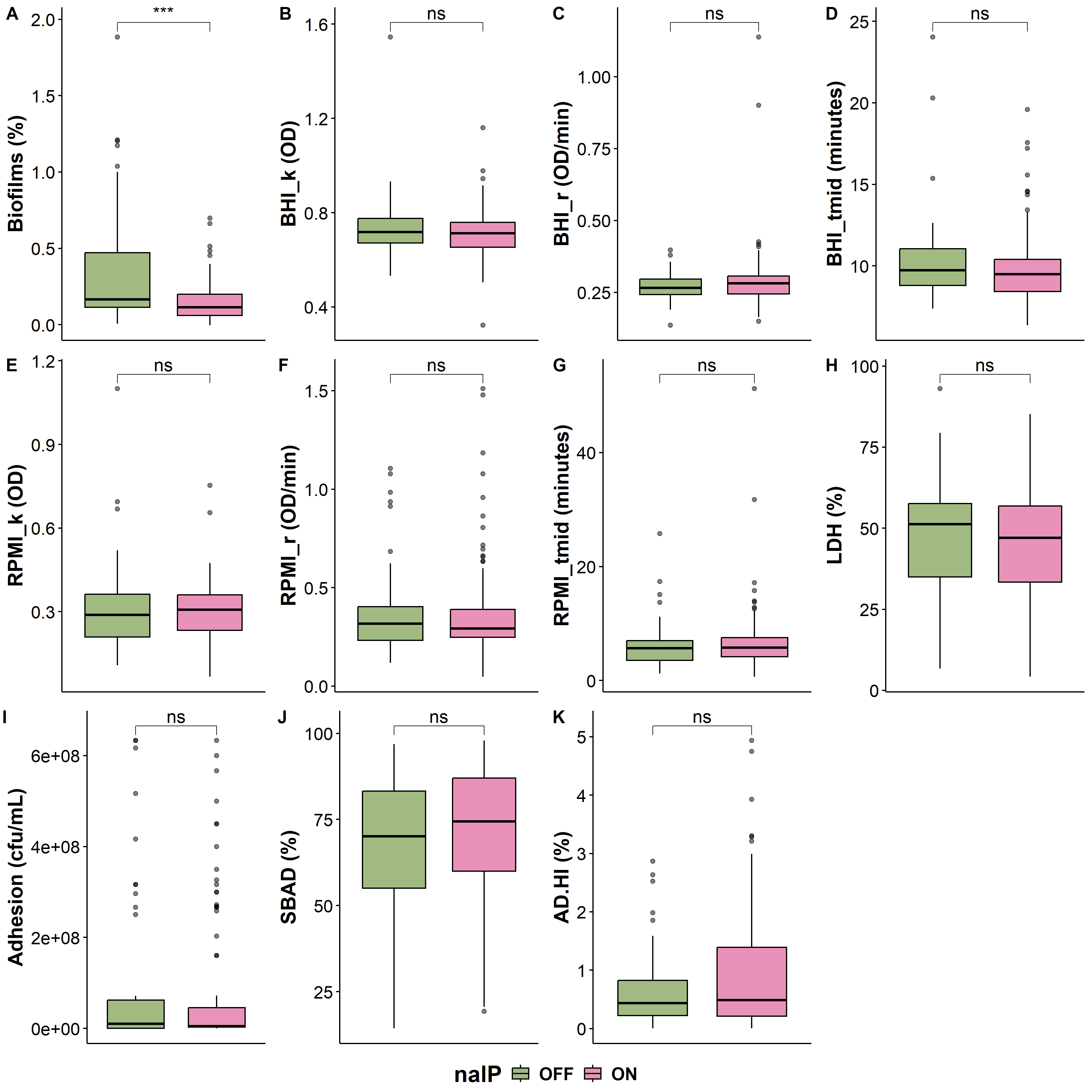


**Figure S16. Association between nalP PV states and phenotypic variation in MenW cc11 isolates.** The expression states of this PV gene were determined for the 163 isolates by a combination of GeneScan and genomic analyses of the repeat tracts. The repeat tracts are located in the reading frame of this gene resulting in ON/OFF expression states. Phenotypic values were compared for isolates grouped by expression state using a Kruskal Wallis and Wilcoxon Rank-Sum test. The Wilcoxon test detected significance for the biofilm trait (p=0.00073) only. Plots: Bar, median; box, interquartile range; line, minimum and maximum; dots, outliers. P values: *, P<0.05; **, P<0.01; ***, P<0.001; ns, not significant.


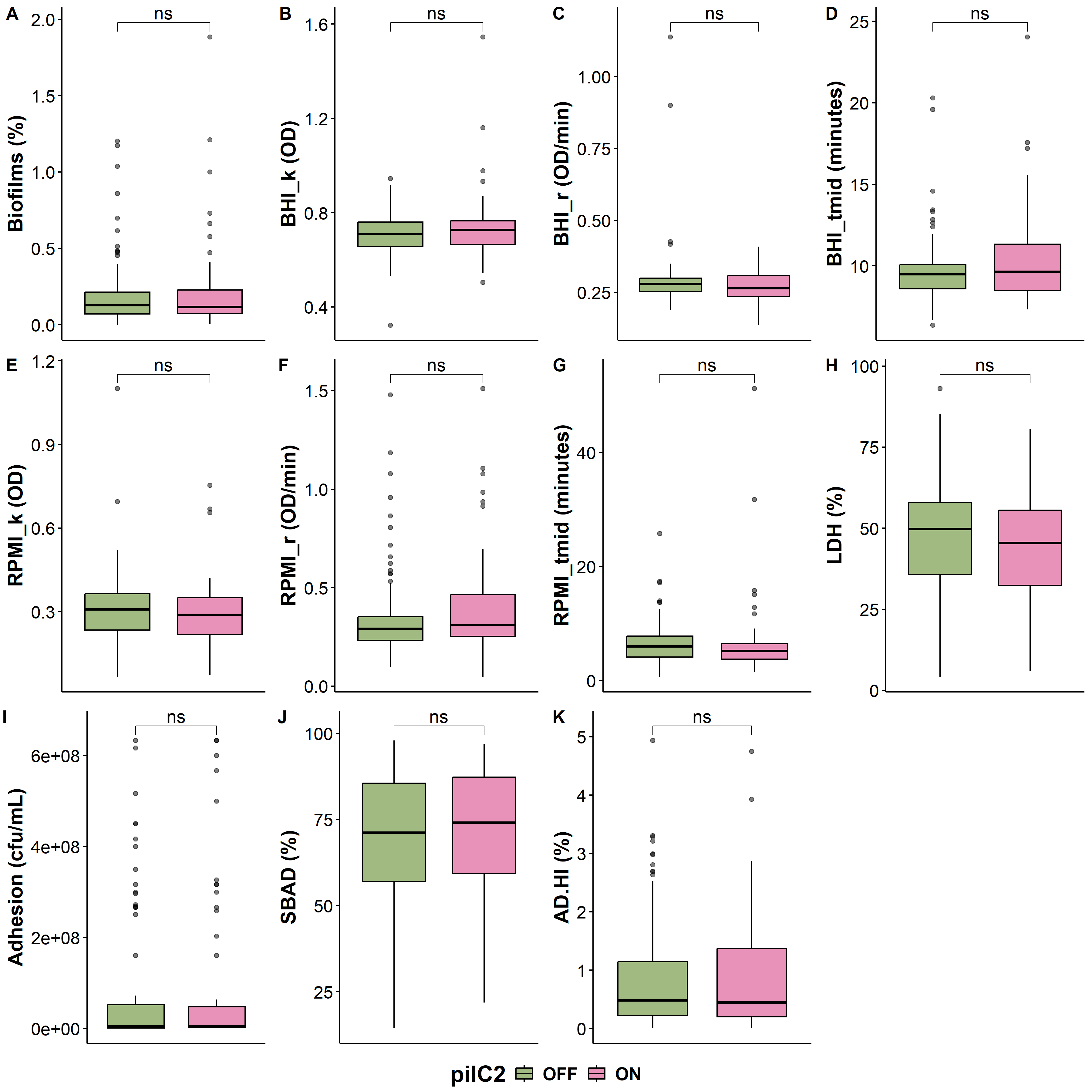


**Figure S17. Association between pilC2 PV states and phenotypic variation in MenW cc11 isolates.** The expression states of this PV gene were determined for the 163 isolates by a combination of GeneScan and genomic analyses of the repeat tracts. The repeat tracts are located in the reading frame of this gene resulting in ON/OFF expression states. Phenotypic values were compared for isolates grouped by expression state using a Kruskal Wallis and Wilcoxon Rank-Sum test. The Wilcoxon test did not detect significance for any of the phenotypes at p<0.05. Plots: Bar, median; box, interquartile range; line, minimum and maximum; dots, outliers. P values: *, P<0.05; **, P<0.01; ***, P<0.001; ns, not significant.


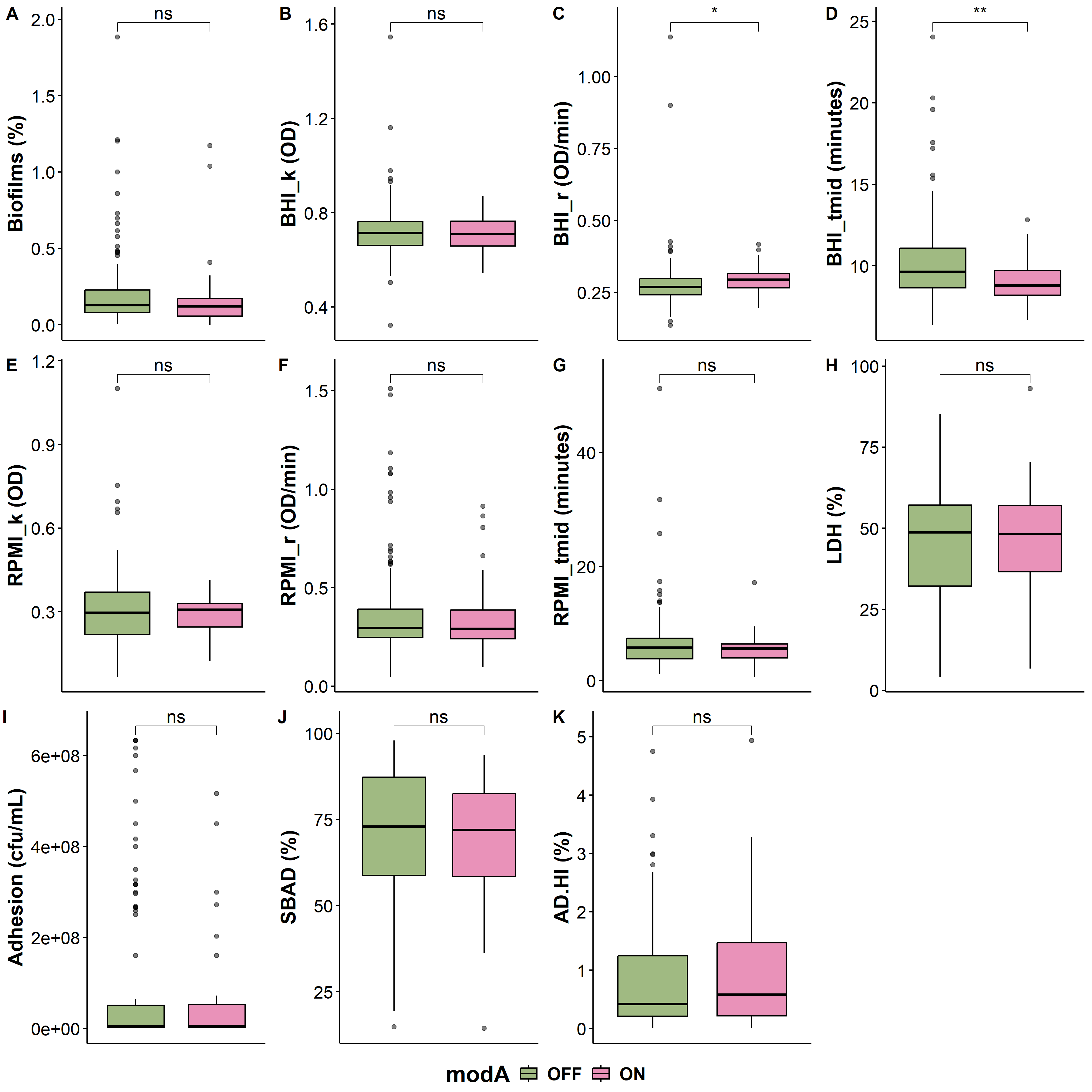


**Figure S18. Association between modA PV states and phenotypic variation in MenW cc11 isolates.** The expression states of this PV gene were determined for the 163 isolates by a combination of GeneScan and genomic analyses of the repeat tracts. The repeat tracts are located in the reading frame of this gene resulting in ON/OFF expression states. Phenotypic values were compared for isolates grouped by expression state using a Kruskal Wallis and Wilcoxon Rank-Sum test. The Wilcoxon test detected significance for the BHI_r (p=0.014) and BHI_tmid (p=0.0054) traits only. Plots: Bar, median; box, interquartile range; line, minimum and maximum; dots, outliers. P values: *, P<0.05; **, P<0.01; ***, P<0.001; ns, not significant.


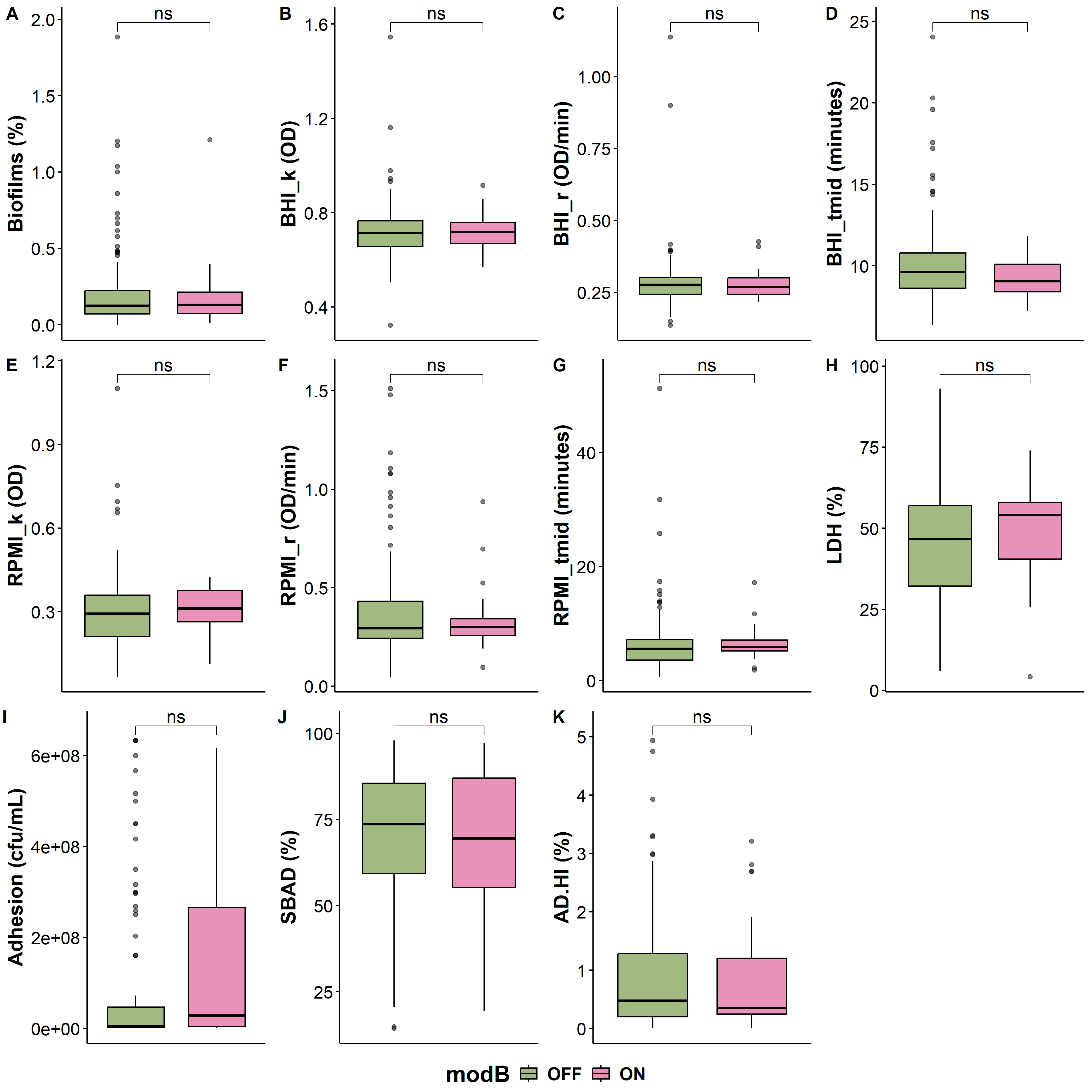


**Figure S19. Association between modB PV states and phenotypic variation in MenW cc11 isolates.** The expression states of this PV gene were determined for the 163 isolates by a combination of GeneScan and genomic analyses of the repeat tracts. The repeat tracts are located in the reading frame of this gene resulting in ON/OFF expression states. Phenotypic values were compared for isolates grouped by expression state using a Kruskal Wallis and Wilcoxon Rank-Sum test. The Wilcoxon test did not detect significance for any of the phenotypes at p<0.05. Plots: Bar, median; box, interquartile range; line, minimum and maximum; dots, outliers. P values: *, P<0.05; **, P<0.01; ***, P<0.001; ns, not significant.


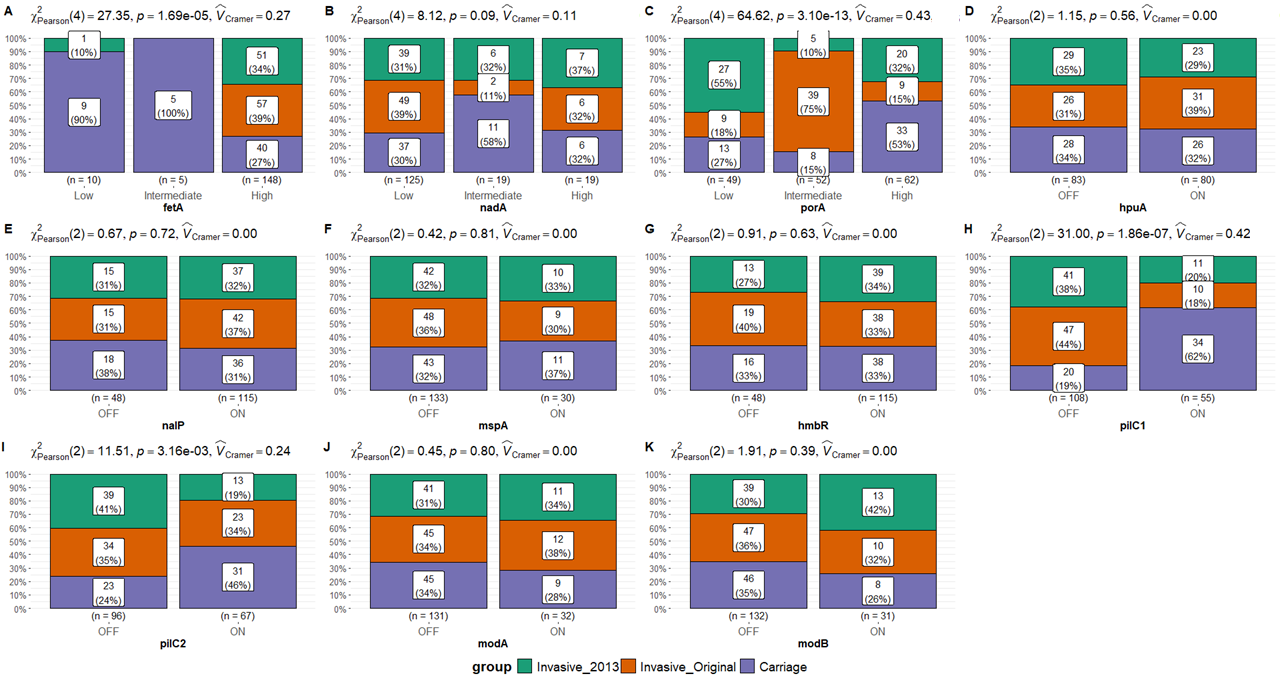


**Figure S20. Distribution and correlation of PV genes with group in MenW cc11 isolates.** We use the Chi-square Test of Independence (χ^2^_Pearson_) and Cramer’s V ( $\hat{V}$_Cramer_) to study the correlation between the PV genes and group. The value for Cramer’s V ranges from 0 to 1, with 0 indicating no association between the variables and 1 indicating a strong association between the variables. Strong associations were observed between group and porA gene while moderate associations were observed between the group and pilC1 gene. Weak associations were observed between the groups and the rest of the PV genes.


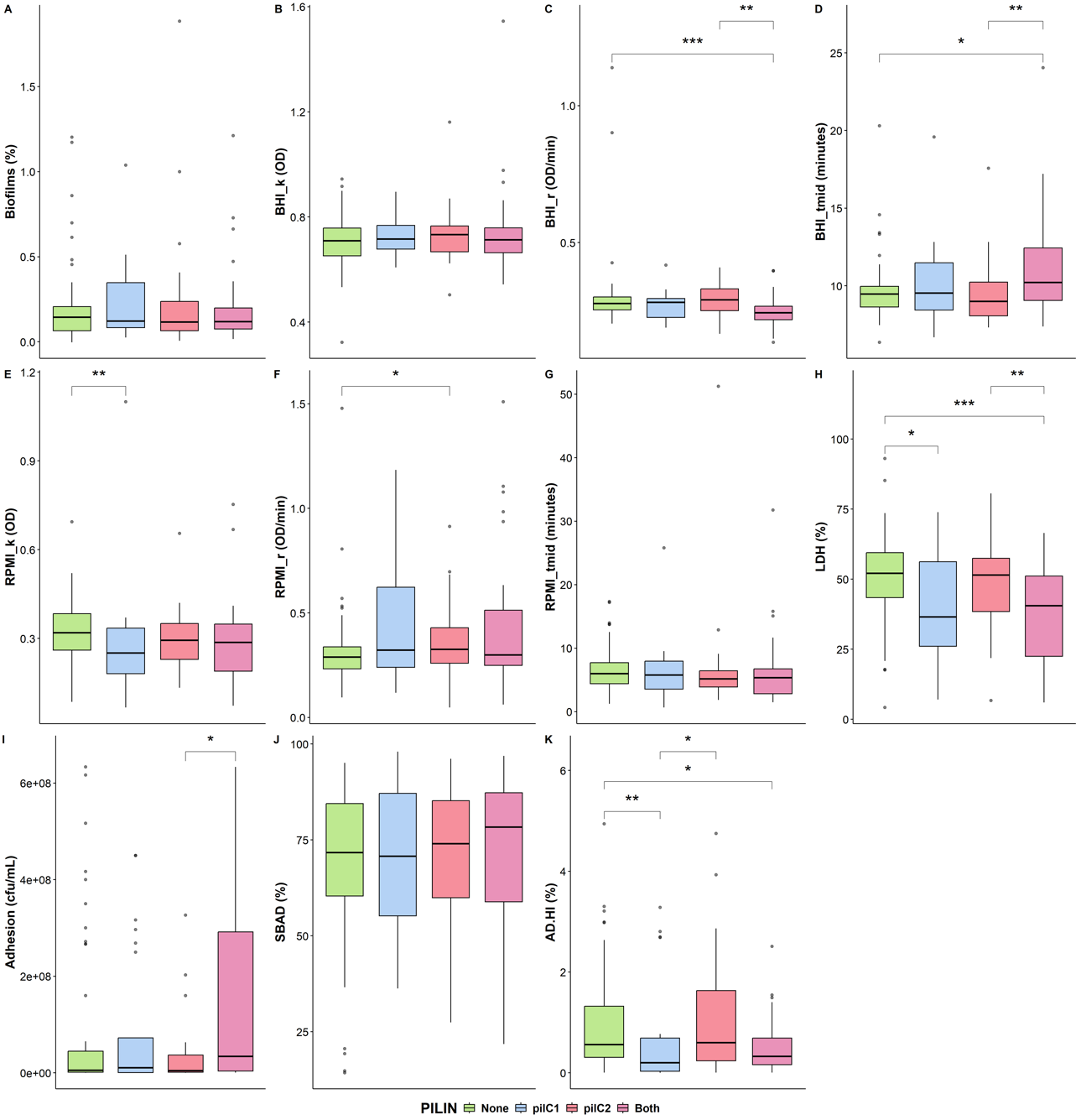


**Figure S21. Association between PILIN phasotype and phenotypic variation in MenW cc11 isolates.** This phasotype is a combination of pilC1 and pilC2 PV gene expressions. The repeat tracts for the individual genes are located in the reading frame of these genes resulting in ON/OFF expression states. Groups are based on a combination of these expression states: None – the two genes are OFF; pilC1 – pilC1 is ON while pilC2 is OFF; pilC2 – pilC1 is OFF while pilC2 is ON; Both – the two genes are ON. Phenotypic values were compared for isolates grouped by the phasotype expression using a Kruskal Wallis and Wilcoxon Rank-Sum test. The Wilcoxon test detected significance for the BHI_r (p=0.0019), BHI_tmid (p=0.035), RPMI_k (p=0.046), LDH (p=0.0017) and AD-HI (p=0.0079) traits only. For Kruskal Wallis test, only significant values (p<0.05) are shown. Plots: Bar, median; box, interquartile range; line, minimum and maximum; dots, outliers. P values: *, P<0.05; **, P<0.01; ***, P<0.001; ns, not significant.


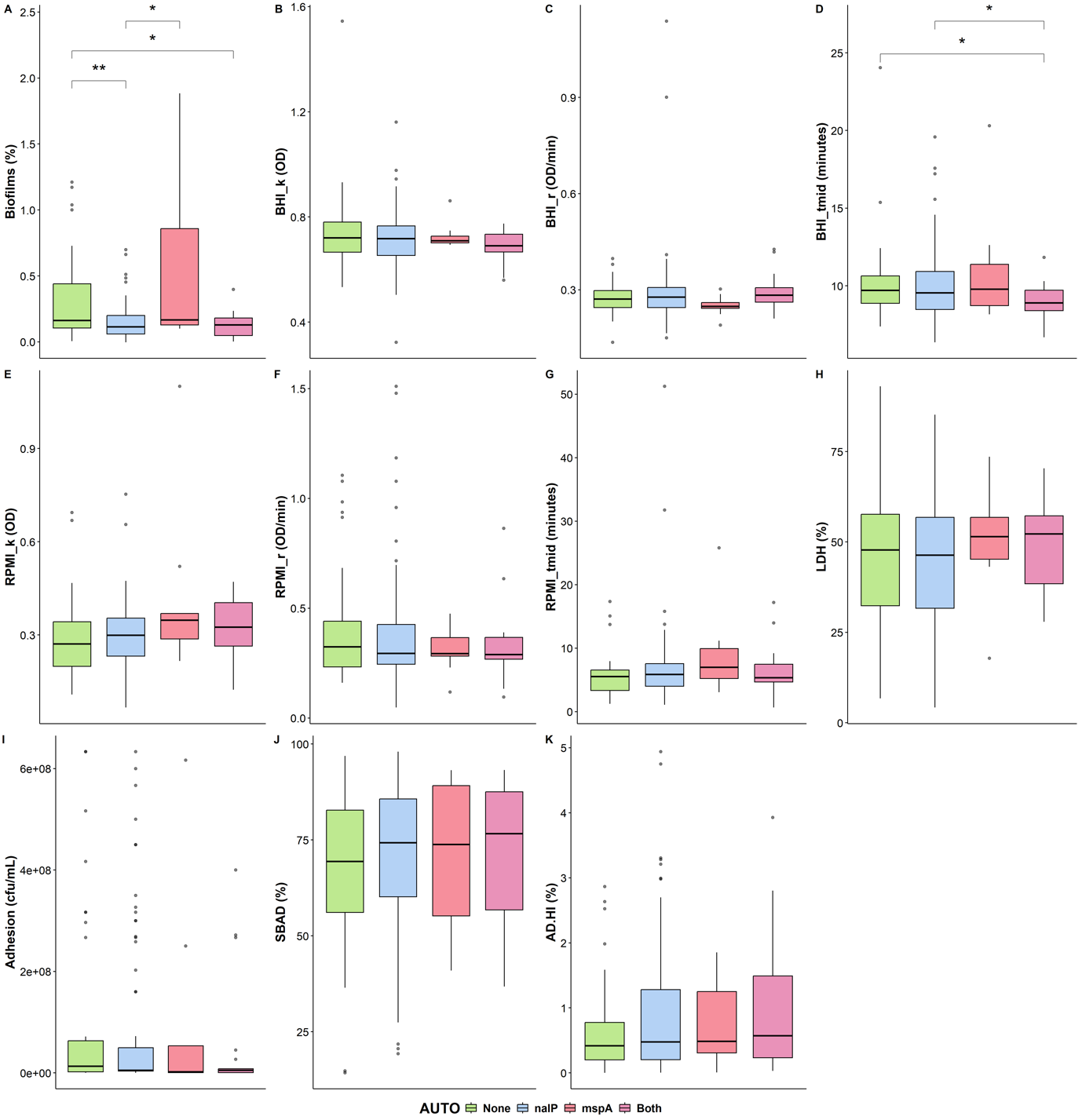


**Figure S22. Association between AUTO phasotype and phenotypic variation in MenW cc11 isolates.** This phasotype is a combination of nalP and mspA PV gene expressions. The repeat tracts for the individual genes are located in the reading frame of these genes resulting in ON/OFF expression states. Groups are based on a combination of these expression states: None – the two genes are OFF; nalP – nalP is ON while mspA is OFF; mspA – nalP is OFF while mspA is ON; Both – the two genes are ON. Phenotypic values were compared for isolates grouped by the phasotype expression using a Kruskal Wallis and Wilcoxon Rank-Sum test. The Wilcoxon test detected significance for the biofilm trait (p=0.0067) only. For the Kruskal Wallis test, only significant values (p<0.05) are shown. Plots: Bar, median; box, interquartile range; line, minimum and maximum; dots, outliers. P values: *, P<0.05; **, P<0.01; ***, P<0.001; ns, not significant.


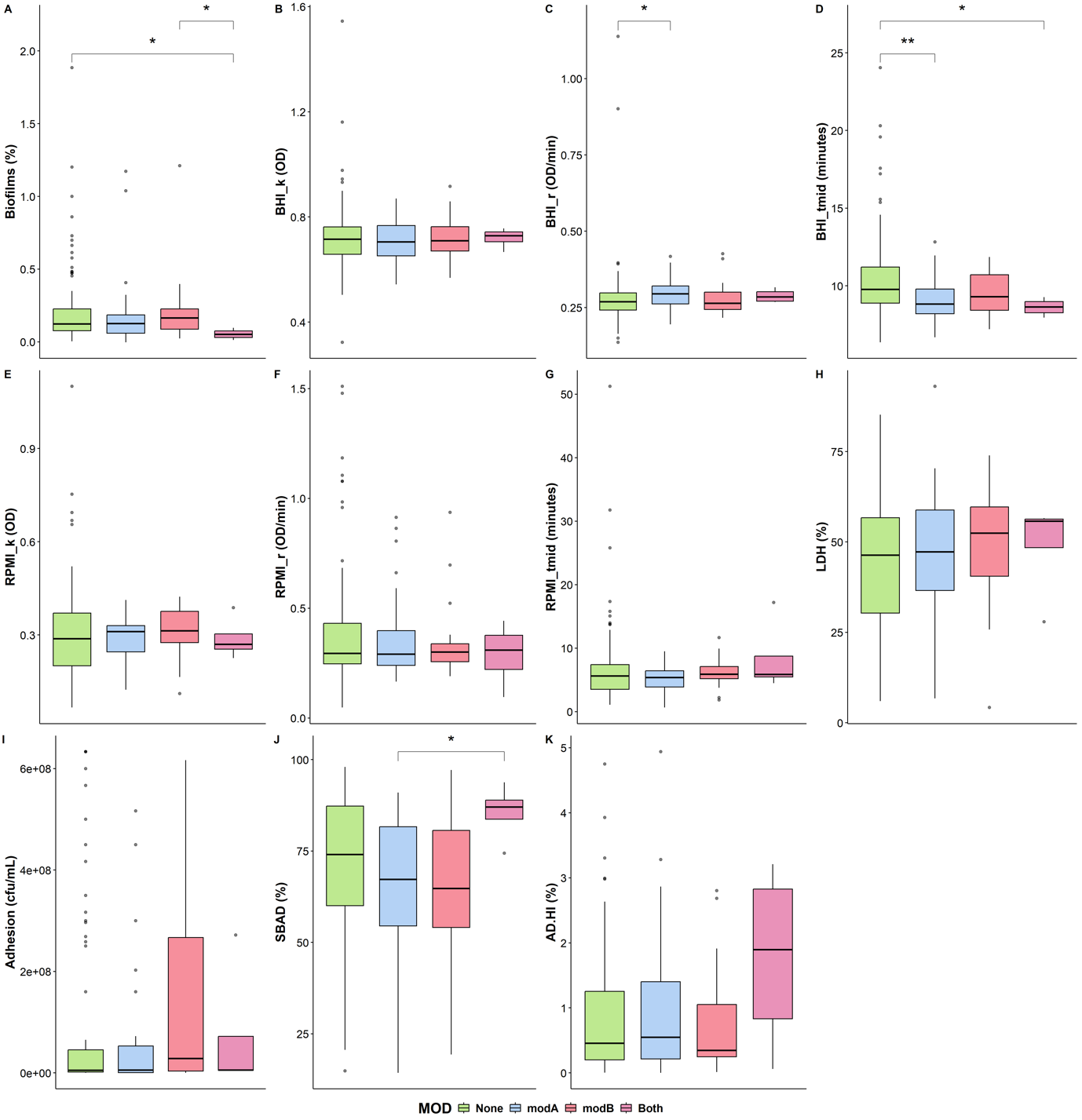


**Figure S23. Association between MOD phasotype and phenotypic variation in MenW cc11 isolates.** This phasotype is a combination of modA and modB PV gene expressions. The repeat tracts for the individual genes are located in the reading frame of these genes resulting in ON/OFF expression states. Groups are based on a combination of these expression states: None – the two genes are OFF; modA – modA is ON while modB is OFF; modB – modA is OFF while modB is ON; Both – the two genes are ON. Phenotypic values were compared for isolates grouped by the phasotype expression using a Kruskal Wallis and Wilcoxon Rank-Sum test. The Wilcoxon test detected significance for the BHI_tmid (p=0.0069) trait only. For the Kruskal Wallis test, only significant values (p<0.05) are shown Plots: Bar, median; box, interquartile range; line, minimum and maximum; dots, outliers. P values: *, P<0.05; **, P<0.01; ***, P<0.001; ns, not significant.


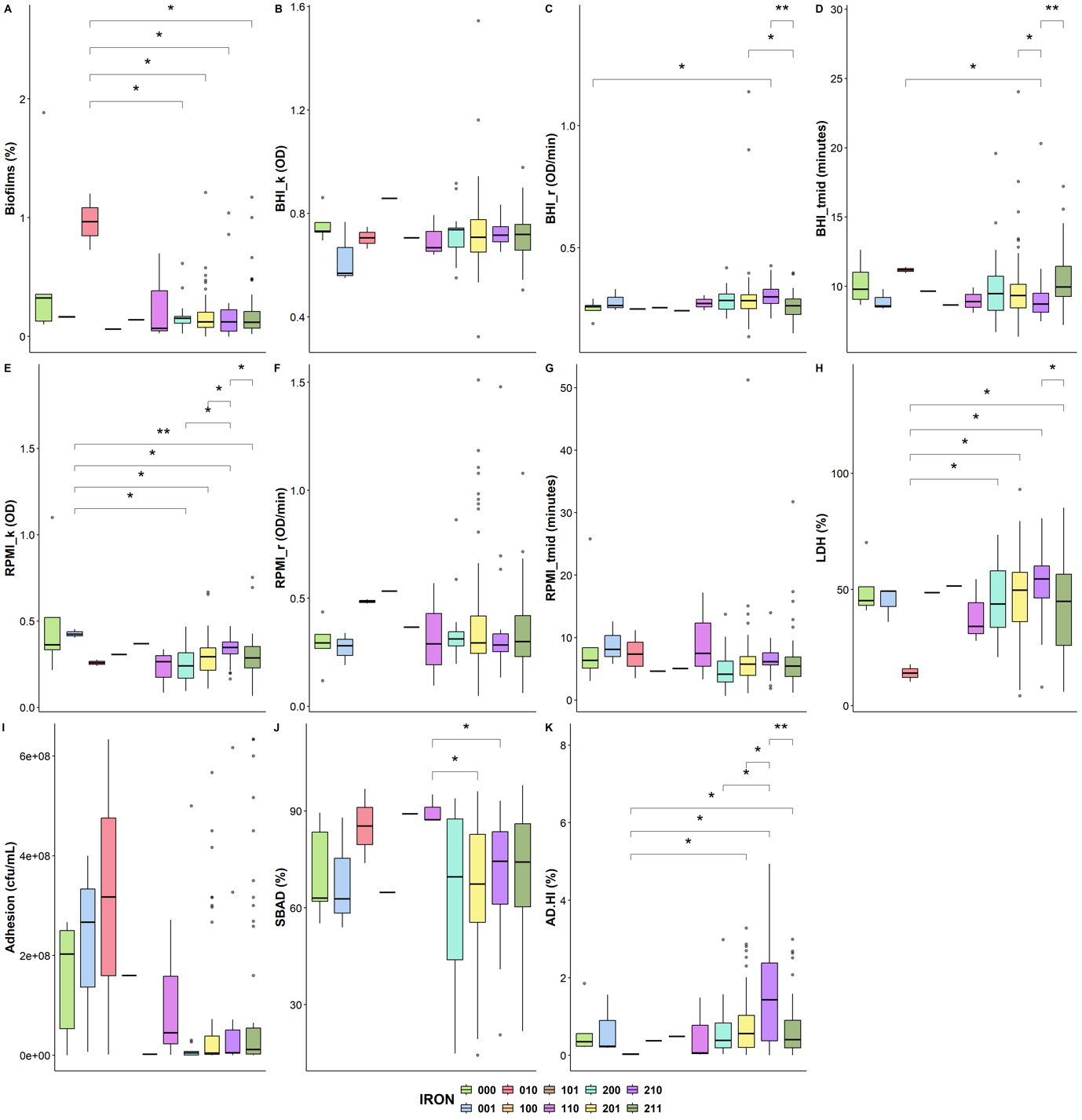


**Figure S24. Association between IRON phasotype and phenotypic variation in MenW cc11 isolates.** This phasotype is an ordered combination of fetA, hpuA and hmbR PV gene expressions. The repeat tract for the fetA gene is located in the promoter resulting in Low (0), Intermediate (1), or High (2) expression states while the repeat tracts for hpuA and hmbR are located in the reading frame of these genes resulting in ON (1) or OFF (0) expression states. Groups are based on the ordered combination of these expression states: 1^st^ digit – expression of fetA; 2^nd^ digit – expression of hpuA; 3^rd^ digit – expression of hmbR. Phenotypic values were compared for isolates grouped by the phasotype expression using a Kruskal Wallis and Wilcoxon Rank-Sum test. The Wilcoxon test detected significance for the RPMI_k (p=0.031) trait only. For the Kruskal Wallis test, only significant values (p<0.05) are shown. Plots: Bar, median; box, interquartile range; line, minimum and maximum; dots, outliers. P values: *, P<0.05; **, P<0.01; ***, P<0.001; ns, not significant.


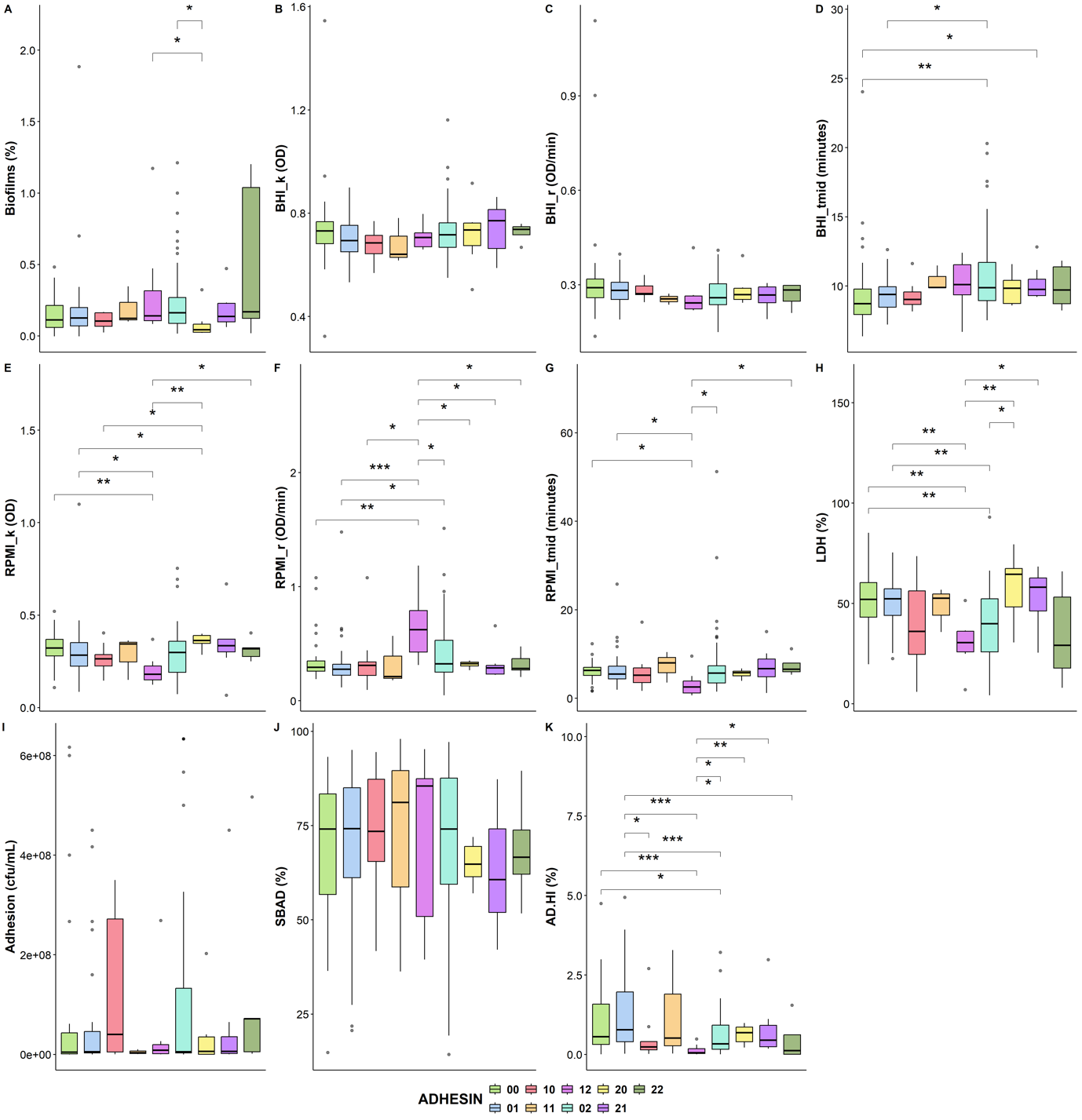


**Figure S25. Association between ADHESIN phasotype and phenotypic variation in MenW cc11 isolates.** This phasotype is an ordered combination of nadA and porA PV gene expressions. The repeat tracts for these genes are located in the promoter resulting in Low (0), Intermediate (1), or High (2) expression states. Groups are based on the ordered combination of these expression states: 1^st^ digit – expression of nadA; 2^nd^ digit – expression of porA. Phenotypic values were compared for isolates grouped by the phasotype expression using a Kruskal Wallis and Wilcoxon Rank-Sum test. The Wilcoxon test detected significance for the BHI_tmid (p=0.038), RMPI_r (p=0.024), LDH (p=0.0012) and AD_HI (p=0.00048) traits only. For the Kruskal Wallis test, only significant values (p<0.05) are shown Plots: Bar, median; box, interquartile range; line, minimum and maximum; dots, outliers. P values: *, P<0.05; **, P<0.01; ***, P<0.001; ns, not significant.


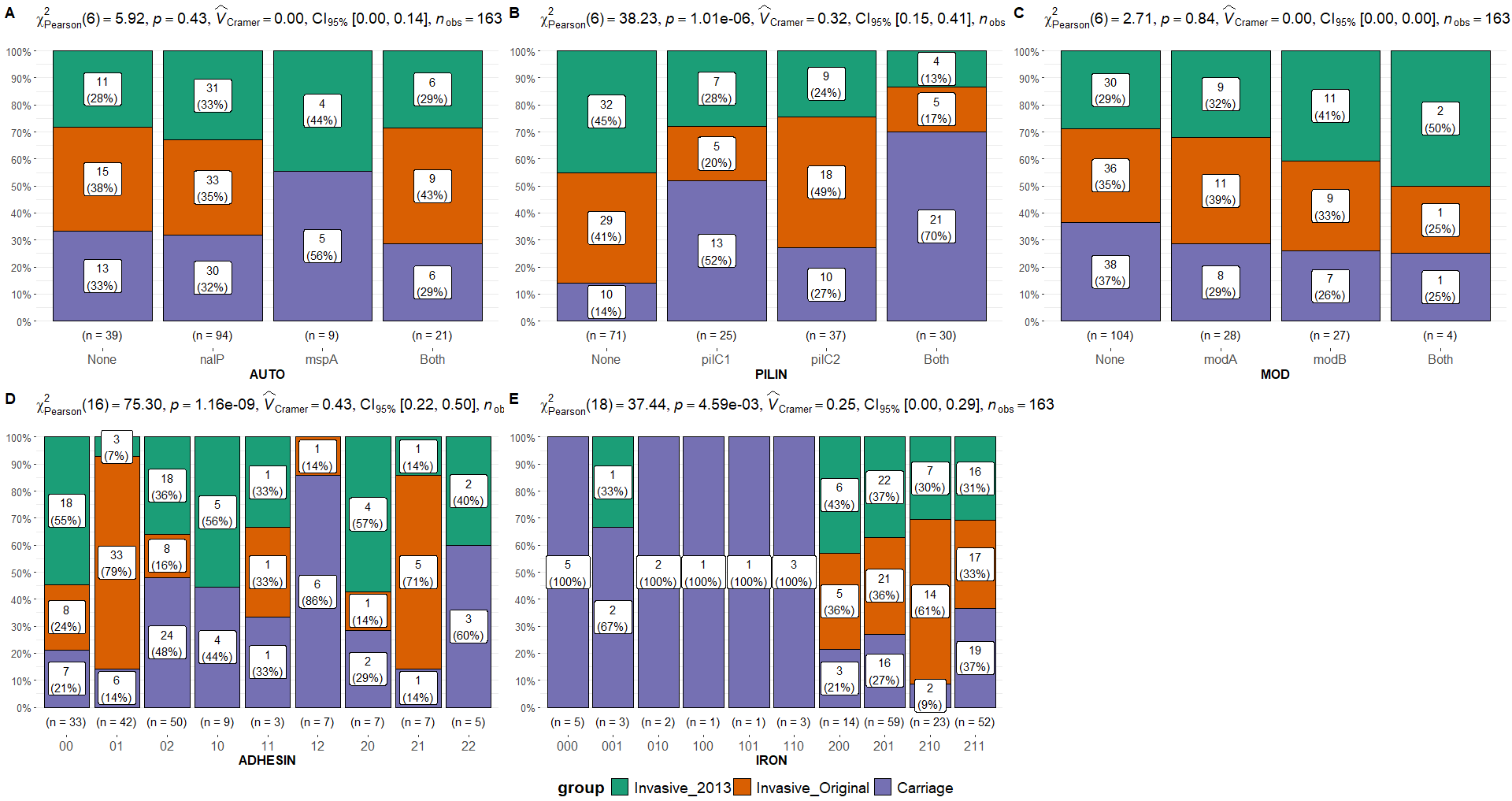


**Figure S26. Distribution and correlation of phasotypes with group in MenW cc11 isolates.** We use the Chi-square Test of Independence (χ^2^_Pearson_) and Cramer’s V ( $\hat{V}$_Cramer_) to study the correlation between the phasotypes and group. The value for Cramer’s V ranges from 0 to 1, with 0 indicating no association between the variables and 1 indicating a strong association between the variables. Strong associations were observed between group and ADHESIN phasotype while moderate associations were observed between the group and both IRON and PILIN phasotypes. Weak associations were observed between the groups and the both AUTO and MOD phasotypes.


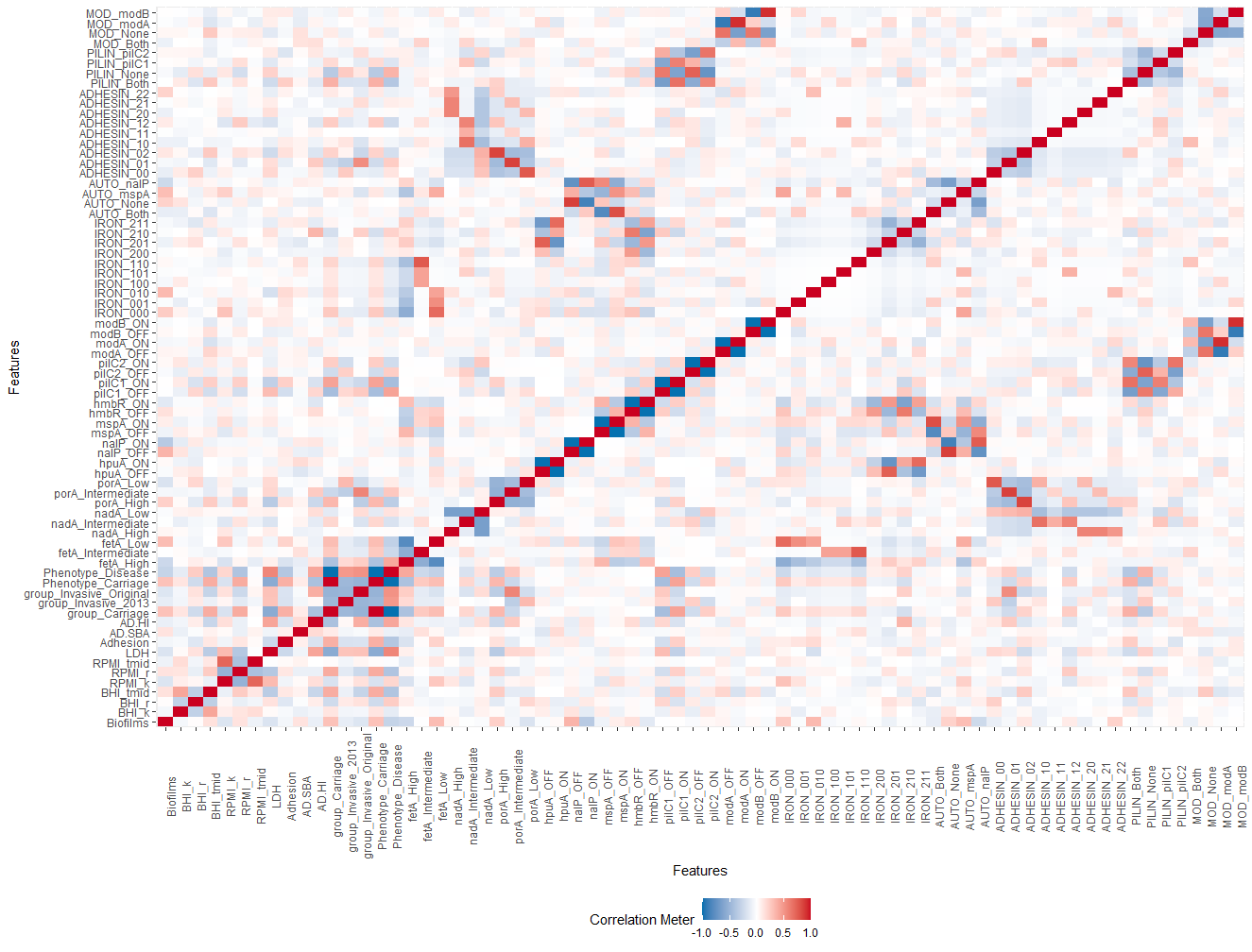


**Figure S27. Correlation matrix for phasotypes, all phase-variable genes and phenotypes.** Dark blue represents an inverse correlation, dark red represent positive correlation while white represents no correlation**.**


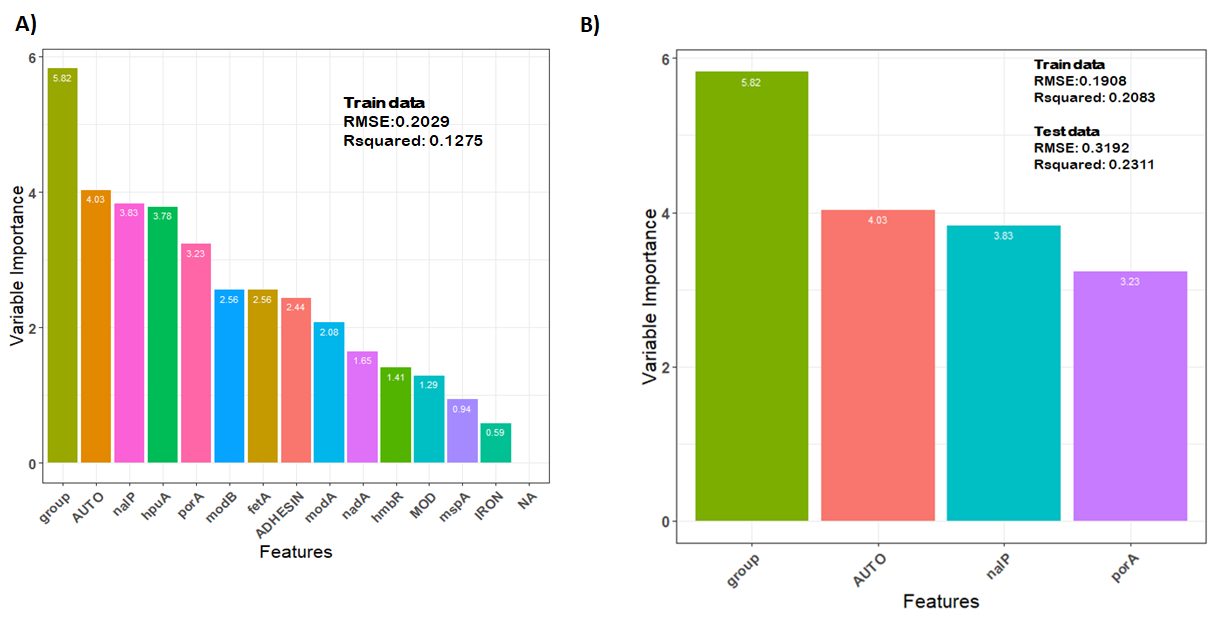


**Figure S28. Genes and phasotypes identified in predicting biofilm formation.** A Recursive Feature Elimination (RFE) with random forest analysis was utilised to predict biofilm formation based on PV states. The PV states were inputted as both the expression states of individual genes or as phasotypes – combinatorial expression states. Panel A, all important phasotypes/PV genes in predicting biofilms. Panel B, phasotypes/PV genes in the best model for predicting biofilms. The y-axis indicates the relative contribution of each determinant to separation of isolates into these groups. Root mean squared error (RMSE), is a model evaluation metrics for continuous models, the lower the RMSE the better the model.


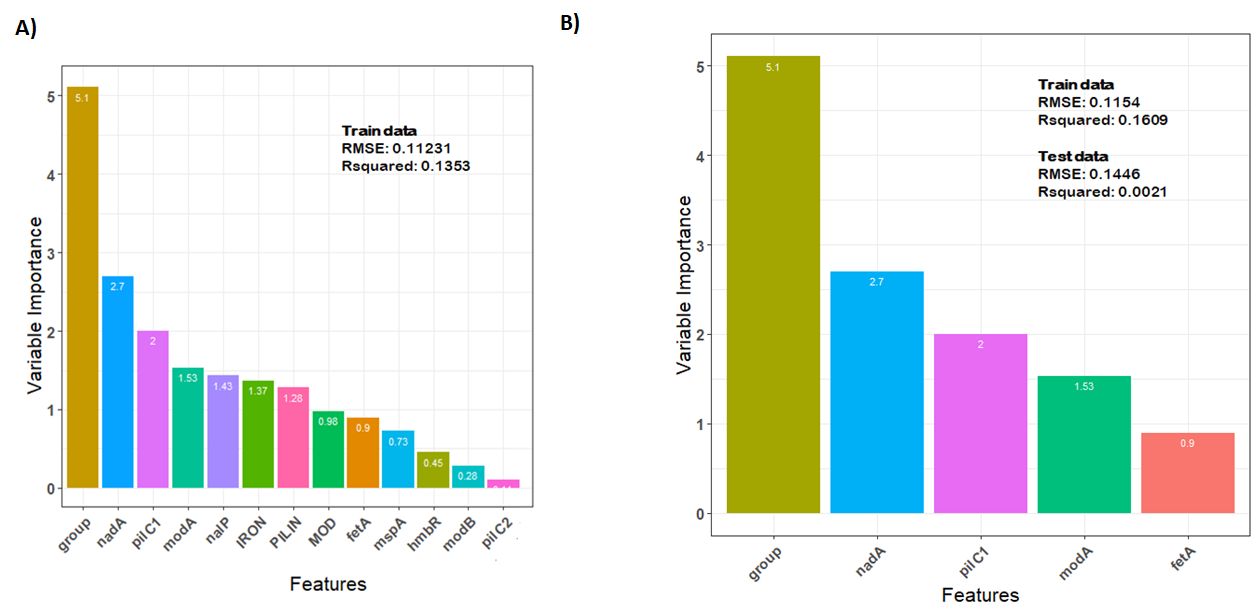


**Figure S29. Genes and phasotypes identified in predicting RPMI_k.** A Recursive Feature Elimination (RFE) with random forest analysis was utilised to predict the carrying capacity in RPMI media (RPMI_k) based on PV states. The PV states were inputted as both the expression states of individual genes or as phasotypes – combinatorial expression states. Panel A, all important phasotypes/PV genes in predicting RPMI_k. Panel B, phasotypes/PV genes in the best model for predicting RPMI_k. The y-axis indicates the relative contribution of each determinant to separation of isolates into these groups. RMSE, is the root mean squared error for each of the model, the lower the RMSE the better the model.
